# Systematic Analysis of a Novel Human Renal Glomerulus-Enriched Gene Expression Dataset

**DOI:** 10.1371/journal.pone.0011545

**Published:** 2010-07-12

**Authors:** Maja T. Lindenmeyer, Felix Eichinger, Kontheari Sen, Hans-Joachim Anders, Ilka Edenhofer, Deborah Mattinzoli, Matthias Kretzler, Maria P. Rastaldi, Clemens D. Cohen

**Affiliations:** 1 Division of Nephrology, University Hospital Zurich, Zurich, Switzerland; 2 Institute of Physiology with Zurich Center of Integrative Human Physiology, University of Zurich, Zurich, Switzerland; 3 Department of Medicine, University of Michigan, Ann Arbor, Michigan, United States of America; 4 Medizinische Poliklinik, University of Munich, Munich, Germany; 5 Renal Research Laboratory, Fondazione IRCCS Policlinico & Fondazione D'Amico per la Ricerca sulle Malattie Renali, Milan, Italy; University of Calgary, Canada

## Abstract

Glomerular diseases account for the majority of cases with chronic renal failure. Several genes have been identified with key relevance for glomerular function. Quite a few of these genes show a specific or preferential mRNA expression in the renal glomerulus. To identify additional candidate genes involved in glomerular function in humans we generated a human renal glomerulus-enriched gene expression dataset (REGGED) by comparing gene expression profiles from human glomeruli and tubulointerstitium obtained from six transplant living donors using Affymetrix HG-U133A arrays. This analysis resulted in 677 genes with prominent overrepresentation in the glomerulus. Genes with ‘*a priori*’ known prominent glomerular expression served for validation and were all found in the novel dataset (e.g. CDKN1, DAG1, DDN, EHD3, MYH9, NES, NPHS1, NPHS2, PDPN, PLA2R1, PLCE1, PODXL, PTPRO, SYNPO, TCF21, TJP1, WT1). The mRNA expression of several novel glomerulus-enriched genes in REGGED was validated by qRT-PCR. Gene ontology and pathway analysis identified biological processes previously not reported to be of relevance in glomeruli of healthy human adult kidneys including among others axon guidance. This finding was further validated by assessing the expression of the axon guidance molecules neuritin (NRN1) and roundabout receptor ROBO1 and -2. In diabetic nephropathy, a prevalent glomerulopathy, differential regulation of glomerular ROBO2 mRNA was found.

In summary, novel transcripts with predominant expression in the human glomerulus could be identified using a comparative strategy on microdissected nephrons. A systematic analysis of this glomerulus-specifc gene expression dataset allows the detection of target molecules and biological processes involved in glomerular biology and renal disease.

## Introduction

The majority of renal diseases leading to end-stage renal diseases (ESRD) are initiated by glomerular alterations [1]. Hereditary, immune-mediated and metabolic disorders can cause such glomerulopathies, but the understanding of the pathomechanism of the most common glomerular diseases is still limited.

The renal glomerulus is capable of filtering large volumes of plasma while efficiently retaining most proteins within the circulation [2]. The development and maintenance of normal glomerular structure and function requires successful signaling and coordination between all glomerular cells, as shown by the critical requirement for vascular endothelial growth factor-A (VEGFA) production by podocytes for normal endothelial and mesangial cell development and function [3], [4].

Podocytes are a unique cell type in the glomerulus [5]. Several genetic studies clearly demonstrated that mutations of proteins, which are preferentially or specifically expressed in this cell type, can cause renal disease leading to the disruption of the filtration barrier, rearrangement of the actin cytoskeleton and ultimately glomerular failure (e. g. α-actinin-4, nephrin, podocin) [6]. Additional studies revealed that proteins regulating the plasticity of the podocyte actin cytoskeleton, such as podocalyxin [7], FAT1 [8], Nck1 and Nck2 [9], synaptopodin [10] and Cathepsin L [11], are also crucial for the function of the glomerular filtration barrier. But also signaling mechanisms in the podocyte including signals from the slit diaphragm (SD) or from the glomerular basal membrane (GBM) are important (e.g. PLCE1, TRPC6, PTPRO, ILK, uPAR [12], [13], [14]). Transcriptional regulation of genes expressed in the glomerulus plays a key role for functional integrity. Transcription factors that have been associated with glomerular disorders include WT1, FOXC2, LMX1B, TCF21, PAX2 and others [15]. Furthermore the composition and charge of the GBM (e.g. LAMB2, COL4A3, −4, −5), the endothelial cells with their unique fenestration (e.g. EHD3) [6] and the mesangial cells play a crucial role. Studies could outline important species-dependent differences in glomerular gene expression, e.g. megalin was shown to be the target autoantigen in experimental Heyman nephritis is absent from the human glomerulus, whereas PLA2R seems to be a target autoantigen in human idiopathic membranous nephropathy [16]. As the glomerular transcriptome of rodents and humans show significant differences, a reliable and comprehensive human data set is required.

Identification of additional human glomerular-enriched genes and proteins as well as molecular mechanisms and gene networks represent a promising approach to the understanding of development and function of the glomerulus and its derangement in glomerular diseases.

In the present study, gene expression profiles from human glomeruli and tubulointerstitium obtained from transplant living donors were compared to each other in order to generate a human renal glomerulus-enriched gene expression dataset (REGGED). REGGED aims to facilitate the identification of gene products and mechanisms important in regulation of renal glomerular structure and function. The mRNA expression for several novel glomerular-enriched genes was verified by qRT-PCR. Gene ontology analysis by Database for Annotation, Visualization and Integrated Discovery (DAVID), as well as the Kyoto Encyclopedia of Genes and Genomes (KEGG) database identified pathways and gene categories well-known to be associated with glomerular biology but additionally pathways not previously reported to be involved in glomeruli of healthy human adult kidneys. Axon guidance, identified by this means as a biological process in human mature glomeruli, was used to validate our approach and the expression of the axon guidance molecules neuritin (NRN1) and roundabout receptor ROBO1 and -2.

In summary, REGGED represents a resource for the glomerular research community to interrogate genes identified by *in vitro* systems, rodent models, or human genetic screens for their enrichment in the glomerular compartment. It further will help to prioritize biological processes in functional studies on glomerular biology.

## Results

### Generation of a human renal glomerulus-enriched gene expression dataset (REGGED) and comparison with published data sets

**Figure 1:**
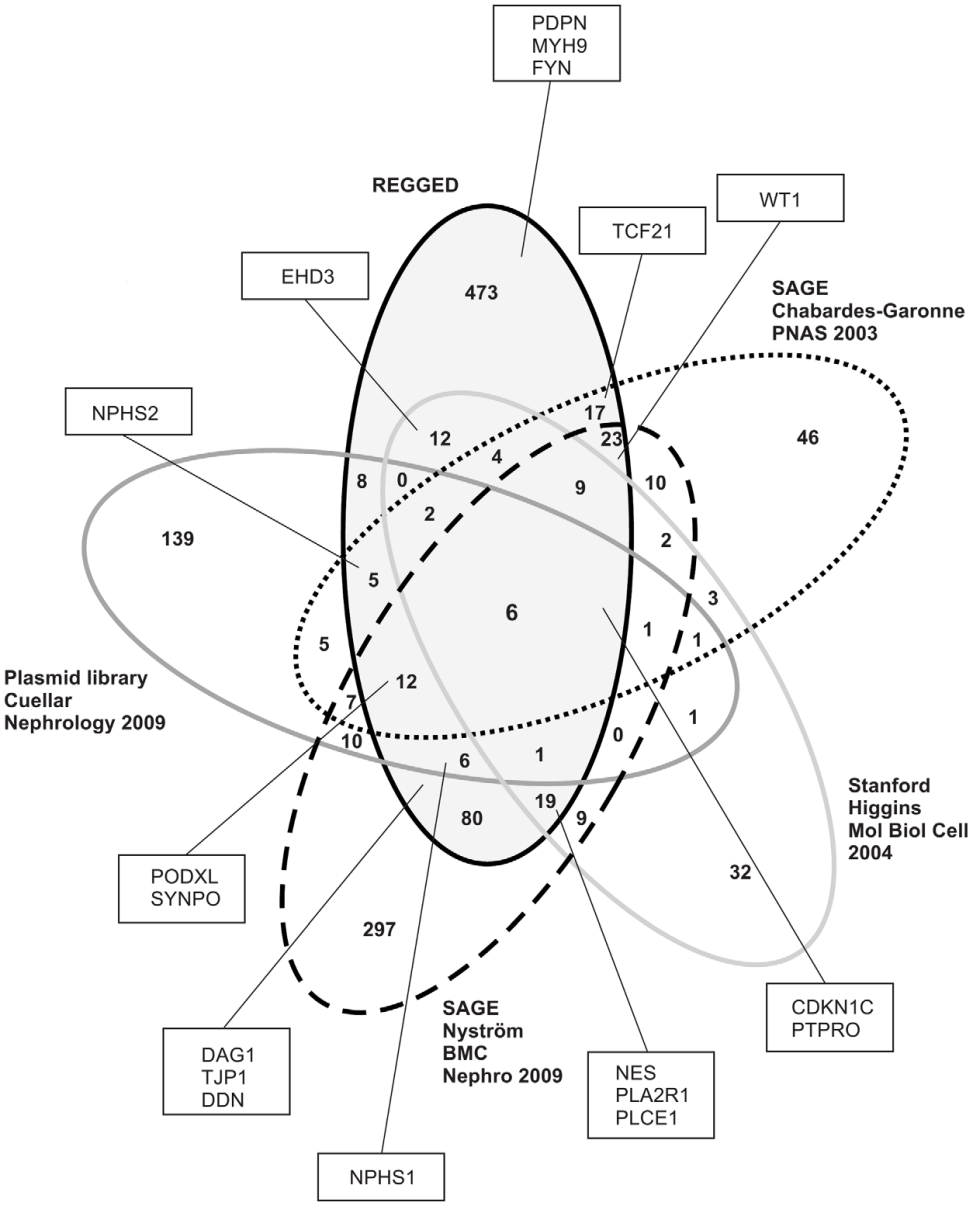
Venn diagram for five human glomerular data set reports. Established glomerular genes are shown in squares. REGGED is the only data set covering all such preselected glomerular gene products. The overlap among the five glomerulus-enriched gene lists is limited (see Table 1).

To identify genes restricted to or enriched in the glomerulus, we compared gene expression profiles of isolated human glomeruli with the tubulointerstitial compartment from biopsies of living donors using Affymetrix HG-U133A arrays. By application of the algorithms given in the Methods section a total of 817 probesets were identified as being glomerular-enriched. After removing unannotated probesets and redundant probesets a list of 677 glomerular-overrepresented genes remained (Supplementary Table S1). For validation of the dataset an arbitrary list of known genes with specific or pronounced expression in the renal glomerulus of different species was generated and compared with REGGED (Supplementary Table S2). Known prominent glomerular transcripts such as CDKN1, DAG1, DDN, EHD3, MYH9, NES, NPHS1, NPHS2, PDPN, PLA2R1, PLCE1, PODXL, PTPRO, SYNPO, TCF21, TJP1, WT1 were all found in the novel expression dataset REGGED (Figure 1) [15], [17], [18], [19], [20], [21], [22], [23], [24], [25]. In a next step, a comparative analysis of different expression platforms was performed. To this end we focused on human data sets and used data published by Chabardes-Garonne et al [26], Higgins et al. [27], Cuellar et al [28], and Nyström et al. [29]. The two SAGE profiling analyses by Chabardes-Garonne and Nyström identified 153 [26] and 492 genes [29], respectively, as being predominantly expressed in the glomerulus compared with other parts of the nephron. The Stanford cDNA microarray profiling by Higgins et al. resulted in 102 [27] glomerular markers, while the plasmid library by Cuellar identified 205 [28] glomerular-enriched genes. Table 1 summarizes the characteristics of the 5 analyses including the present study. The comparison of the 5 different approaches is illustrated in Figure 1 and Supplementary Table S3. REGGED contains a number of genes with established function in glomerular biology, which were not previously found in human data sets (e.g. FYN, MYH9, PDPN) [13], [20], [30], [31]. Similar to He et al [32], who compared rodent and human data sets, only 6 genes were identified in all studies, namely the podocyte-expressed genes CDKN1C, PTPRO, SPARC, and PLAT, the endothelial marker EMCN, and the mesangial-expressed IGFBP5.

**Table 1:** Summary of the characteristics of the five methods.

Investigator	Method	Species	Total Features on the platform	Glomerulus- enriched genes reported	Selection criteria	Reference
Chabardes-Garonne et al	SAGE profiling	Human	50 000 tags	153	P<0.01, seven-fold or more difference with at least three nephron libraries	[26]
Higgins et al	Stanford cDNA microarray profiling	Human	41.859 probes	102	Cluster analysis, genes predominantly expressed in glomeruli than others	[27]
Cuellar et al	cDNA library (Plasmid cloning)	Human	5000 clones	205	Sequence analysis and comparison with UniGene database	[28]
Nystrom et al	SAGE profiling	Human	22907 tags	492	Comparison to pooled SAGE libraries for non-glomerular tissues and cells	[29]
Lindenmeyer et al	Affymetrix HG-U133A	Human	22283 probesets	677	Comparison of isolated glomeruli with tubulointerstitial compartment from biopsies of living donors	

### Gene Ontology and Pathway Analysis

**Figure 2:**
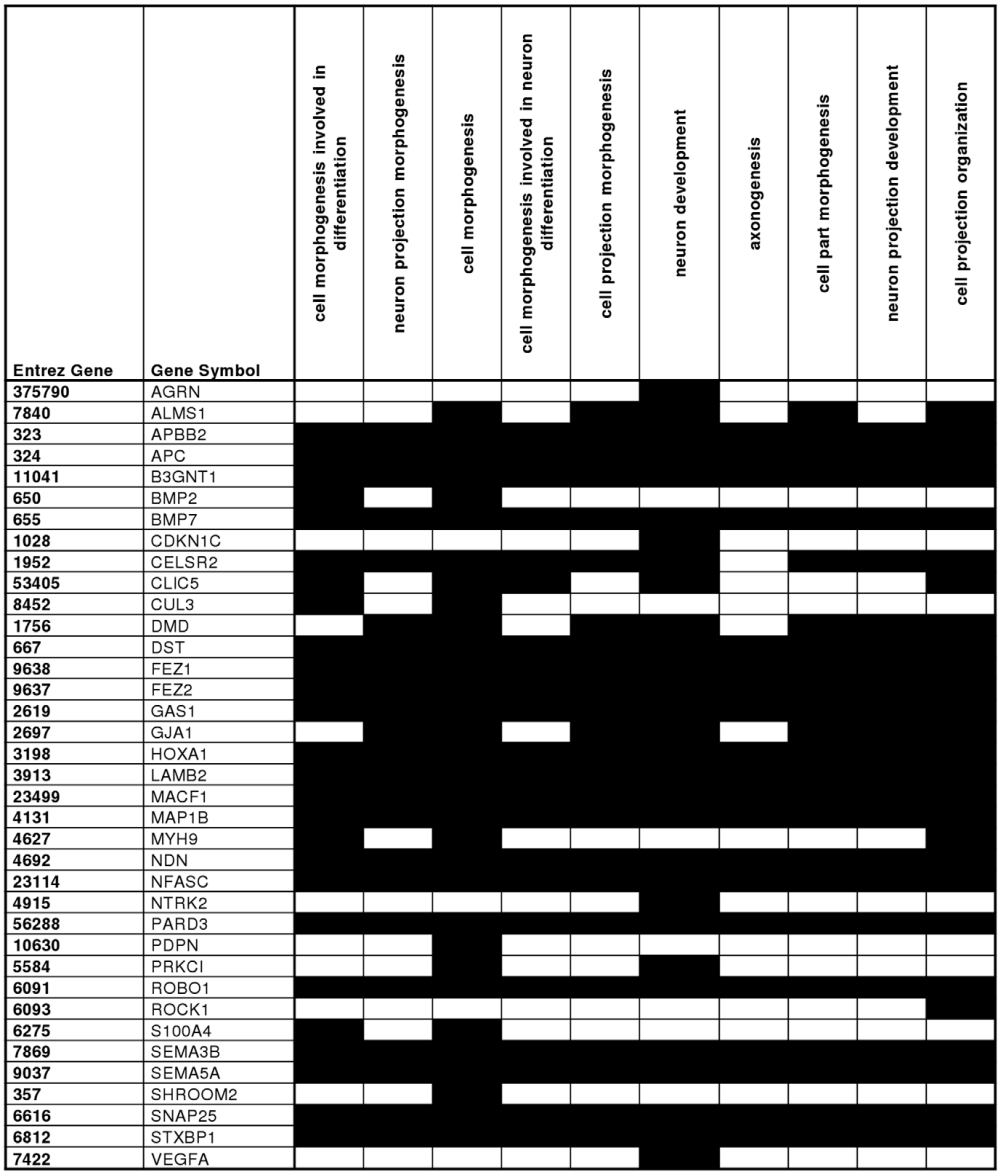
DAVID Functional Cluster Analysis – genes involved in functional cluster 2. Genes which are involved in the respective biological GO-term are shown in black.

As gene lists per se need to be integrated in a functional context, we mapped the REGGED genes into different biological categories according to gene ontology (GO). A relative ranking of the association of the various GO-categories with respect to the gene list was carried out employing DAVID, a web-based tool developed for GO-ranking. Although GO enrichment analysis has some limitations [33], it is an efficient means to extract the biological meaning behind large gene list. DAVID analysis of all 677 glomerular-enriched genes yielded 197 GO-categories (Supplementary Table S4; cut-off p-value 0.05, Fold enrichment >1.5). To receive a more comprehensive and structured view of the annotation terms a DAVID clustering analysis under high stringency conditions was performed resulting in ten annotation clusters matching the statistical criteria [(*p*<0.05), fold enrichment >1.5 and an enrichment score of at least 2.0]. Each of these clusters was composed of at least 3 annotation terms, with an enrichment score of 2.01–7.34 (Supplementary Table S5 and Supplementary Figure S1). As an example functional cluster 2 is shown in more detail in Figure 2; it is composed of ten GO terms that apply to identical sets of genes.

Subsequently, the aforementioned DAVID annotation tool was used for identification of putative KEGG pathways associated with glomerular biology. The glomerular-enriched genes could be mapped to 105 pathways, 12 of which were significantly enriched with glomerular-associated genes (p<0.05, Fold enrichment >1.5) (Supplementary Table S6). For instance, we found the KEGG terms “regulation of actin cytoskeleton”, “focal adhesion” and “tight junction” to be significantly enriched pathways, which are known to be of relevance for glomerular biology. Interestingly, “axon guidance”, one of the GO terms associated with glomerular-enriched genes, was also pinpointed in the KEGG pathway analysis.

### Comparison with neuronal related genes and genes mainly expressed in smooth muscle cells as well as in muscle and heart

Several studies have shown that glomerular podocytes and neurons share some specific characteristics. For instance, both cells are highly arborized, have a common cytoskeletal organization and signaling processes, and share several expression-restricted proteins, such as NPHS1, LRRC7, PTPRO, KHDRBS3, or SYNPO [34], [35]. We therefore conducted a digital differential display analysis to compare normal adult brain cDNA libraries (total of 27,891 sequences) with other tissue libraries except for testis (143,877 sequences). Testis libraries were excluded because of the significant similarity of its transcriptome to the one of brain [36]. DDD takes advantage of the UniGene database by comparing the number of times that sequences from different libraries are assigned to a particular UniGene cluster. This analysis produced 186 UniGene clusters that were potentially specific to the brain pool. Combining this list of UniGene clusters with a literature-derived list of neuronal related molecules [37], we generated a list of 414 neuronal/brain-associated genes (Supplementary Table S7) and compared it with REGGED. This comparison revealed that 38 of the 414 neuron/brain-associated genes were present in the glomerular dataset (Supplementary Table S8).

More and more studies have demonstrated that myosin or smooth muscle-related molecules participate in glomerular biology and development of renal disease [20], [30], [38], [39]. We therefore conducted two further DDD analyses comparing a coronary artery smooth muscle cell cDNA library (total of 7,220 sequences) or normal adult muscle and heart cDNA libraries (total of 12,861 sequences) with other tissue libraries (176,036 and 163,171 sequences, respectively). These analyses resulted in 90 and 161 UniGene clusters, respectively, which were potentially enriched in smooth muscle cells or muscle and heart. Comparison of the smooth muscle cell dataset with REGGED revealed an overlap of 9 genes (Supplementary Table S9). For the comparison of the muscle and heart dataset with REGGED an overlap of 17 genes was found (Supplementary table S10) including genes previously reported to be expressed in the glomerulus such as GSN, NEBL or TNNC1 [40], [41]. As expected, MYH9, for which genetic variants were found to be associated with non-diabetic end-stage renal disease [20], [30], is missing in these lists as it encodes a non-muscle myosin chain.

### Validation by real-time RT-PCR

**Figure 3:**
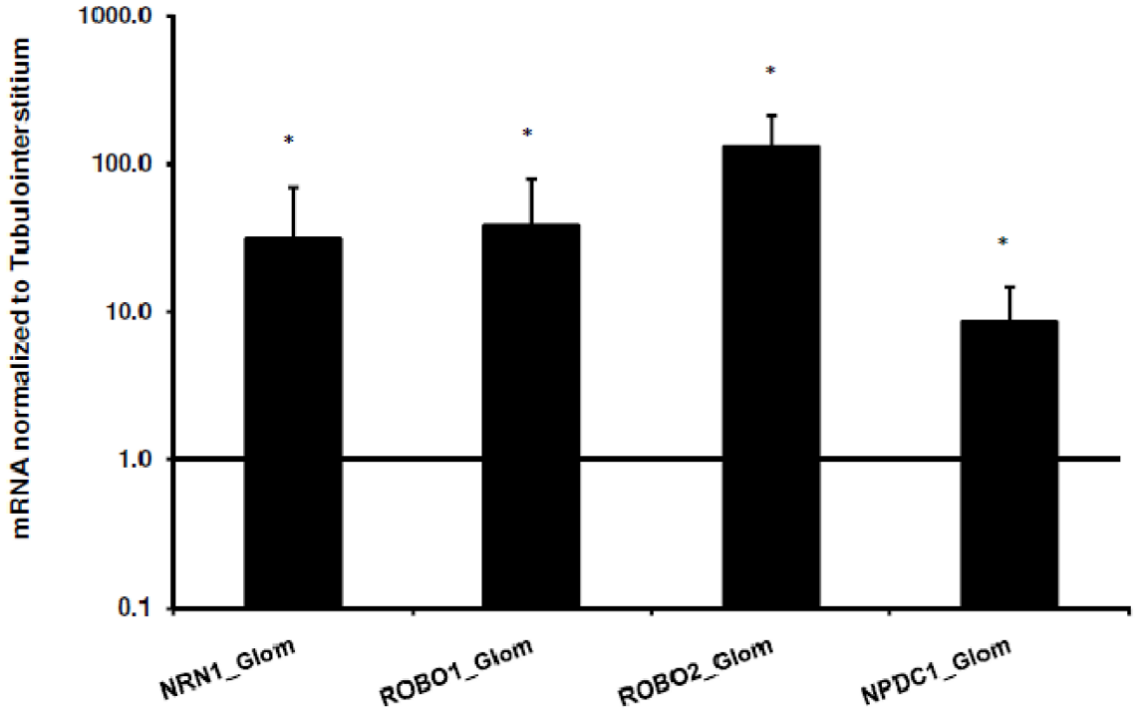
Evaluation of neuron-associated genes by real-time RT-PCR. Expression of NRN1, ROBO1, ROBO2, and NPDC1 mRNA in an independent cohort of microdissected samples of allograft donors normalized to the tubulointerstitial expression (n = 10). * p<0.05; ** p<0.01. The data shown are normalized to the mean of the two reference genes, GAPDH and 18S rRNA.

DDD as well as GO and KEGG analyses revealed axon guidance-related genes to be overrepresented in the renal glomerulus. We decided to focus on 4 selected neuronal associated genes in closer detail: neural proliferation differentiation and control 1 (NPDC1), neuritin (NRN1), roundabout receptor 1 and 2 (ROBO1, ROBO2, the latter not on the HG-U133A array and therefore not in REGGED, but found in rodent glomeruli by [42]). For initial further validation real-time RT-PCR was performed on an independent cohort of microdissected samples of allograft donors. Figure 3 displays the results for NPDC1, NRN1, ROBO1 and ROBO2 mRNA expression. The abundance of all these genes was significantly higher in glomeruli than in the tubulointerstitium ranging from approximately 9-fold (NPDC1) to 130-fold (ROBO2).

### Protein expression of neuritin (nrn1) and robo1 in cultured podocytes and in healthy human control kidneys

**Figure 4:**
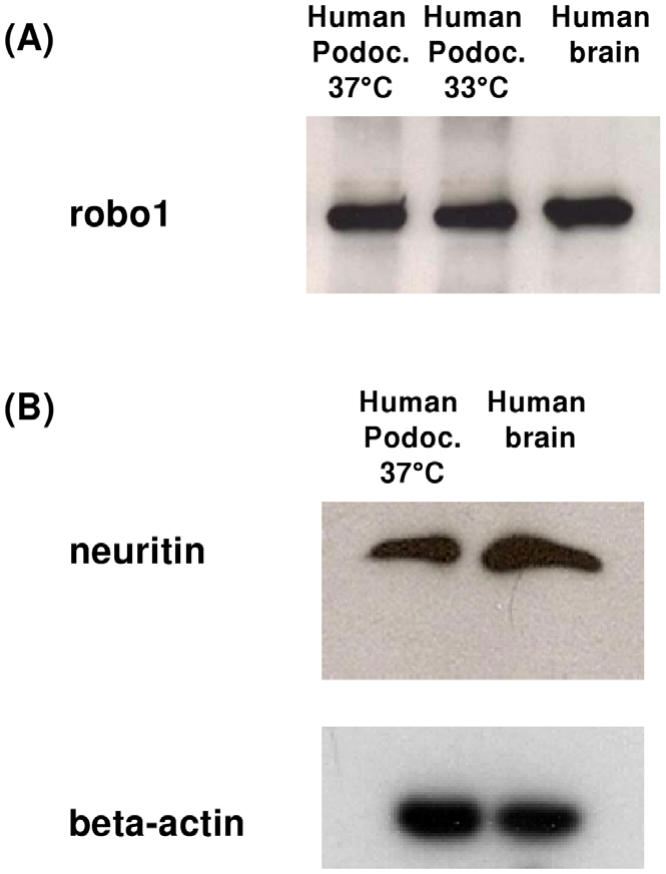
Western Blot analysis of robo1 (A) and neuritin (B) in human podocytes. For robo1 (A) and neuritin (nrn1) (B), a band of the expected size was found in a human podocyte cell line; human brain lysate served as a positive control. Beta-actin was used as an internal loading control.

The protein expression of neuritin and roundabout receptor 1, both known to be involved in axon guidance and neurite outgrowth, was assessed by Western blot in podocytes. Both proteins, robo1 and neuritin, were found to be expressed on protein level in a human podocyte cell line; human brain lysate served as a positive control (Figure 4A and B).

**Figure 5:**
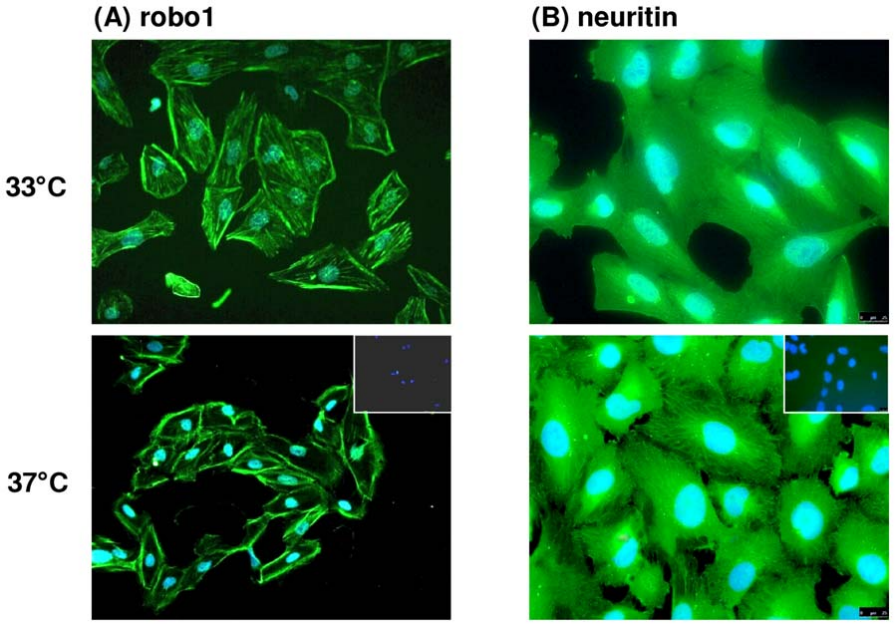
Immunofluorescence of robo1 (A) and neuritin (B) in human podocytes. In undifferentiated, immortalized podocytes (33°C) the expression of robo1 seemed to be more cytoskeletal, while in differentiated cells (37°C) a more intensive staining as well as a more pronounced staining at the cell border was found. For neuritin a cytoskeletal staining pattern could be observed in undifferentiated and differentiated cells with in the latter pronounced staining of stress fibers.

Immunofluorescence experiments were performed to analyze the cellular localization of robo1 and neuritin in podocytes (Figure 5) as both proteins were well expressed on mRNA level in these cell types. In undifferentiated immortalized podocytes (33°C), a cytoskeletal staining for robo1 was found, while in differentiated cells (37°C) a more intense staining of the cell border was observed (Figure 5A). For neuritin a cytoskeletal staining pattern could be observed in undifferentiated and differentiated cells, in the latter a more pronounced staining of stress fibers was seen (Figure 5B).

**Figure 6:**
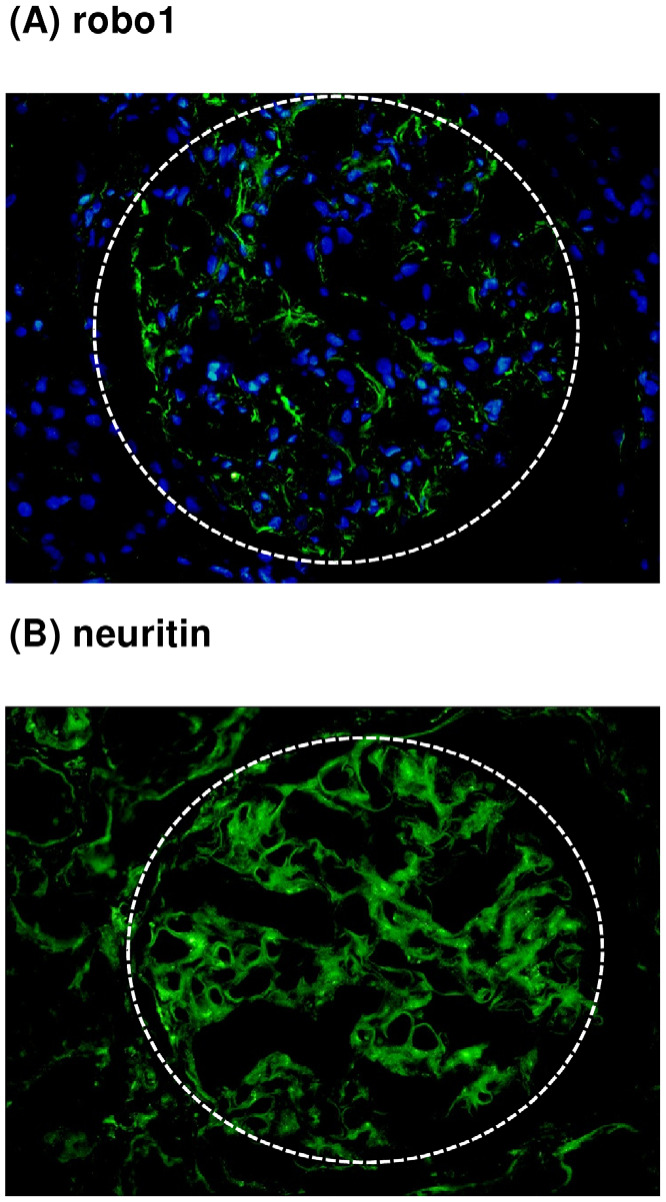
Immunofluorescence of robo1 (A) and neuritin (B) in human control kidneys. Immunofluorescence for robo1 (A) shows a constitutive expression in glomeruli, while there is no expression in the tubulointerstitium. Immunostaining for neuritin (B) shows a clear glomerular expression associated to some positivity in the tubulointerstitium. The hatched line displays the glomerular contour (indirect immunofluorescence, DAPI nuclear counterstain, 200X).

To evaluate the protein expression of both axon guidance molecules *in vivo*, immunofluorescence staining for robo1 and neuritin was performed on an independent set of healthy control kidneys. For robo1 expression in the glomerulus could be detected, while the staining was either completely absent or showed a minimal scattered positivity in the tubulointerstitial compartment. Neuritin showed a clear staining of the glomerulus, but also some positive signal in the tubulointerstitium (Figure 6A and B). This is consistent with REGGED and the qRT-PCR experiments indicating an enhanced but not specific glomerular expression of neuritin in the human adult healthy kidney.

### Regulation of mRNA for the axon guidance molecules NRN1, ROBO1 and ROBO2 in different renal diseases

**Figure 7:**
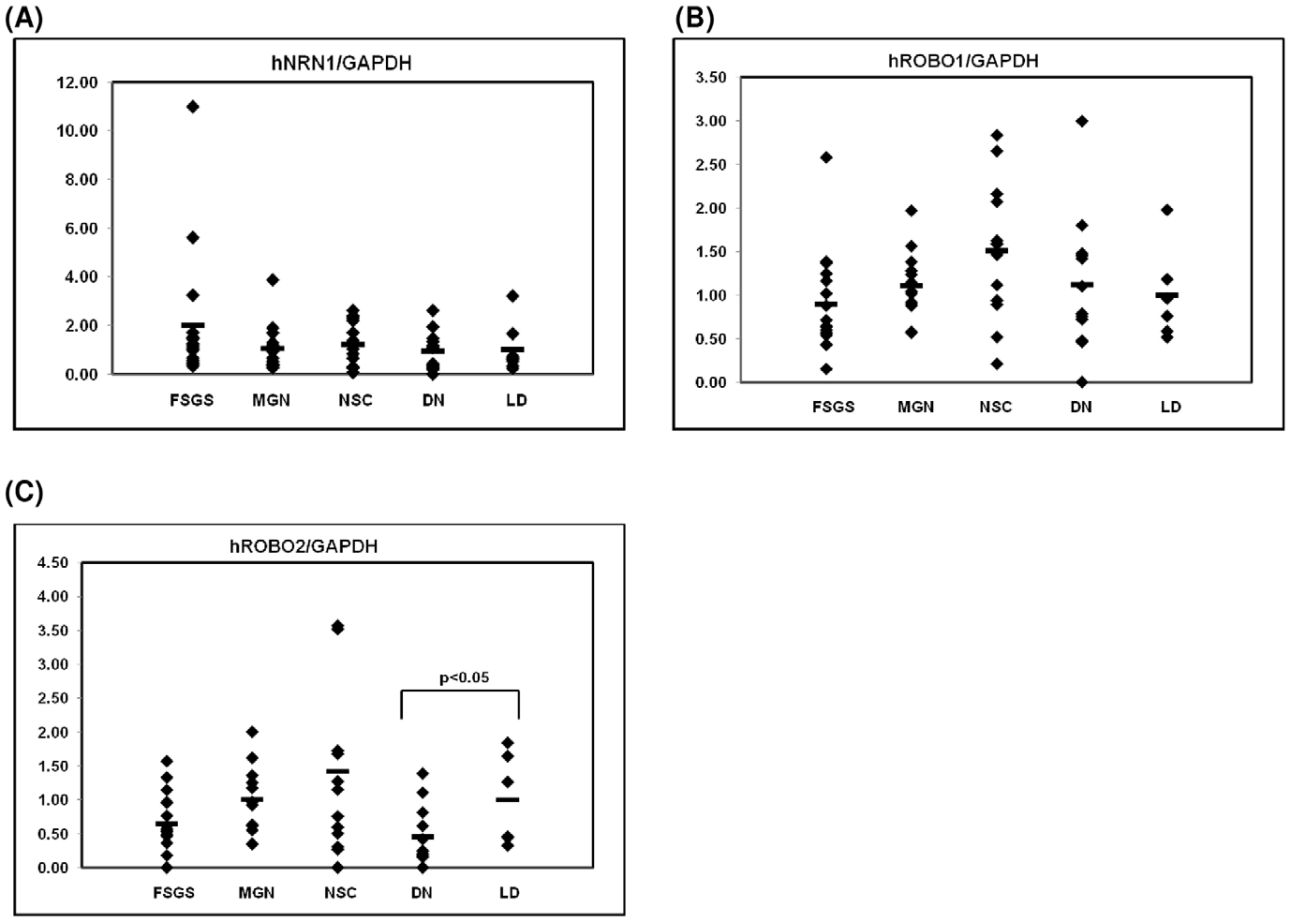
Glomerular mRNA expression of NRN1, ROBO1 and 2 mRNA in human DN, NSC, FSGS and MGN. Levels of mRNA for NRN1 (A), ROBO1 (B) and ROBO2 (C) were quantified in microdissected glomeruli from controls (n = 8), patients with established DN (DN, n = 14), NSC (n = 14), FSGS (FSGS, n = 17) and from patients with MGN (n = 17). ROBO2 was significantly down-regulated in diabetic nephropathy compared to control samples as indicated by the respective p-value, while ROBO1 and NRN1 showed no regulation. The graphs show expression ratios of each gene normalized to hGAPDH.

To investigate intrarenal disease associated regulation of NRN1, ROBO1 and ROBO2 mRNA, we assessed the renal expression on microdissected glomeruli of cohorts with diabetic nephropathy (DN), nephrosclerosis (NSC), focal-segmental glomerulosclerosis (FSGS), membranous glomerulonephropathy (MGN) and pretransplant biopsies. Compared to normal glomeruli obtained from living donors we found a significantly lower expression of ROBO2 mRNA in DN and a trend towards a decreased expression in FSGS patients. ROBO1 and NRN1 mRNA showed no significant change in the disease cohorts (Figure 7).

## Discussion

We compared microarray gene expression data from human glomeruli and tubulointerstitium obtained from transplant living donors to generate a human renal glomerulus-enriched gene expression dataset which contained 677 glomerulus-enriched or -restricted genes.

Comparison of our study with earlier reports of human glomerular-enriched databases using different techniques resulted in a limited overlap (Figure 1). This is in accordance with a meta-analysis performed by He et al [32] comparing different platforms and species. These results suggest that the differences may result from the different technical platforms, glomerular isolation protocols, normalization and processing of the raw data as well as different categorization criteria. In our study we used the Affymetrix platform which identified the highest number of glomerulus-enriched genes compared to the other techniques, again in accordance with the report of He et al. [32]. REGGED was in comparison to the other human databases clearly enriched for known podocyte- and glomerulus-enriched genes as summarized in Figure 1. Furthermore REGGED contained some genes, such as PDPN, FYN or MYH9, which are known to be of relevance for glomerular biology, but were missing in the other human databases.

To gain further information about the biology represented by this gene set we performed GO and pathway analysis using DAVID and the KEGG database. From GO-analysis, cytoskeleton-associated GO categories were identified as being significantly enriched in the glomerular dataset, which was further confirmed by the KEGG-pathways “regulation of actin cytoskeleton”, “focal adhesion”, and “tight junction”. This is in accordance with several reports indicating that the function of podocytes, one of the three intrinsic glomerular cell types, is dependent on the plasticity of its unique and complex cytoskeletal architecture [43] and that podocyte injury with disruption of their specialized functions leads to proteinuria and foot process effacement.

One characteristic of podocytes is that except in collapsing FSGS the differentiated podocytes do not proliferate. Once podocytes are mature and terminally differentiated they remain in a quiescent state and express cyclin-dependent kinase inhibitors p27 and p57, which are present in REGGED, and do not express markers of proliferation (cyclin A, cyclin D, and Ki-67) [44]. This feature of cell cycle arrest may be the cause why the GO-analysis of the current glomerular dataset revealed several GO categories associated with cell cycle, cell growth and cell death.

Several studies have shown that podocytes share some similarities with neuronal cells. Both cells possess a highly arborized morphology, share many common cytoskeletal proteins such as synaptopodin, drebrin and densin resulting in a common cytoskeletal organization, have a common machinery for process formation [45] and express proteins primarily or exclusively found in neurons and podocytes, e.g. nephrin [46], glomerular epithelial protein 1 (GLEPP1) [47], synaptic vesicle molecule Rab3A and its effector rabphilin-3A [37], the RNA processing protein Sam68-like mammalian protein 2 (SLM2) [34] and the ubiquitin C-terminal hydrolase-L1 (UCH-L1) [48]. DDD analysis showed an enrichment of neuronal associated genes, and gene ontology as well as pathway analysis confirmed an association between neurons and glomeruli by identifying processes such as “neurogenesis” and “axon guidance” as being significantly overrepresented in this glomerular dataset. We selected 4 neuronal genes, which are associated with axon guidance (NRN1, ROBO1, ROBO2) or the control of proliferation and differentiation of neural cells (NPDC1). qRT-PCR analysis on human biopsies confirmed the overrepresentation of these genes in the glomerulus. Of interest, a human glomerular enrichment was only recently reported for NRN1 and NPDC1 [29] but not for ROBO1 and -2.

As “axon guidance” was one of the processes shown to be significantly overrepresented in REGGED, we focused in the further course of the study on genes involved in axon guidance, a process that has not been previously described in the glomerulus of healthy human adult kidneys. Neuritin (NRN1) is a glycosylphosphatidylinositol-anchored protein that is induced by neuronal activity and by the neurotrophins BDNF and NT-3. It promotes neurite outgrowth and arborization as well as neuronal survival [49], [50]. In this study we found neuritin to be enriched in the glomerulus. By immunoblotting and -fluorescence we could demonstrate the expression of neuritin in cultured human podocytes and in glomeruli of healthy human kidneys. Previous studies revealed the involvement of neuritin in tumorigenesis by promoting changes in cell morphology, anchorage-independent growth and tumor formation [51] and demonstrated that its expression could be induced in endothelial cells by hypoxia, implicating a role of neuritin in vessel pathfinding and network formation [52]. Recent studies showed that neuritin expression increased following ischemia and reperfusion in rats [53] and that it mediates NGF-induced axonal regeneration and is deficient in experimental diabetic neuropathy [54].

For ROBO1 and -2 mRNA a glomerular overexpression of up to 100 fold compared to the tubulointerstitium could be observed (Figure 3). Supporting the mRNA results we found by immunoblotting and -fluorescence a clear presence of robo1 in cultured human podocytes and in glomeruli of healthy human control kidneys. Robo1 and -2, members of the roundabout receptor family, are single-transmembrane receptors that respond to secreted slit proteins and act as repellents regulating the migration of neurons and axons, but are also involved in inhibition of leukocyte chemotaxis, tumor angiogenesis and endothelial cell migration [55], [56], [57]. It is known that robo and slit genes are not only expressed in the brain, but can be found in a range of tissues. Previous rodent studies could demonstrate that the slit and robo gene families are expressed in the developing murine kidney and that disruption of the slit-robo signaling is associated with congenital anomalies of the kidney and urinary tract [58], [59], [60]. Furthermore Fan et al. just recently reported that podocyte-specific deletion of *robo2* in mice developed significant albuminuria which was associated with increased glomerular collagen deposition, mesangial matrix expansion and podocyte foot-process effacement [61]. In accordance with these results are our findings of decreased levels of ROBO2 mRNA in human diabetic nephropathy and focal segmental glomerulosclerosis. It is known that axon extension and guidance require a coordinated assembly of F-actin and microtubules. Different studies showed that the slit-robo transduction pathway acts via a specific family of GTPase-activating proteins (GAPs) named slit-robo GAPs (srGAPS). These srGAPs further transmit the signal to the actin cytoskeleton controlling Rho GTPases such as CDC42 or rac1 and thus provide a direct link between slit-robo signaling and actin cytoskeleton [62]. Studies from Kobayashi et al showed that alterations of the activity of the rho family small GTPases leads to changes in actin filament assembly and in foot process formation [45]. In this context, the finding of robo1 and -2 in podocytes indicate a possible role in the regulation of the complex cytoskeletal structure of these cells which is also strengthened by the presence of srGAPs and downstream targets of the slit-robo signaling pathways in our REGGED.

In conclusion, we successfully generated a human glomerulus-enriched gene expression dataset (REGGED) which allowed us to identify novel genes expressed predominantly in the human glomerulus. Pathways which have not previously been associated with glomerular biology were identified. A systematic analysis of this dataset allows the detection of target molecules and biological processes involved in glomerular biology and renal disease. We believe that REGGED will fuel ongoing and future research on glomerular biology and disease.

## Materials and Methods

### Renal biopsies for mRNA analysis

Human renal biopsy specimens were procured in an international multicenter study, the European Renal cDNA Bank-Kroener-Fresenius biopsy bank (ERCB-KFB, see appendix for participating centers [63]). Renal biopsies were obtained after written consent and approval of the ethics committee and in the frame of the European Renal cDNA Bank approved by the specialized subcommittee for internal medicine of the cantonal ethics committee of Zurich. All kidney donors had normal renal function, no proteinuria and no arterial hypertension. Glomeruli and the tubulointerstitial specimen were microdissected as described previously [63]. The data discussed in this publication have been deposited in NCBI's Gene Expression Omnibus [64] and are accessible through GEO Series accession number GSE21785 (http://www.ncbi.nlm.nih.gov/geo/query/acc.cgi?acc=GSE21785) and will also be made available online at http://www.nephromine.org.

For validation of the microarray data, qRT-PCR on biopsies from an independent cohort of living donors (LD, n = 10) was performed. Furthermore, cohorts of patients with diabetic nephropathy (DN, n = 14), focal segmental glomerulosclerosis (FSGS; n = 17), membranous nephropathy (MGN; n = 17), nephrosclerosis (NSC; n = 14) and controls (living donors (LD) n = 8) were used for gene expression analysis by qRT-PCR (Supplementary Table S11).

### Target preparation

RNA was isolated as described previously [63]. Total RNA was reverse-transcribed (RT) and linearly amplified according to a protocol previously reported for tubulointerstitial specimen [65] and glomeruli [66], respectively. The fragmentation, hybridization, staining and imaging were performed according the Affymetrix Expression Analysis Technical Manual.

### Microarray Data Analysis

#### Normalization

To compare the respective glomerular and tubulointerstitial gene expression profiles we performed background adjustment, quantile normalization and probeset summarization using Robust Multichip Analysis (RMA) using RMAexpress version 0.3 [67] with settings from previous studies [65].

#### Comparison

The comparison of tubulointerstitial and glomerular expression relies on the assumption that only a small subset of genes shows compartment specific expression and as a corollary almost all show equal expression. However, as glomerular and tubulointerstitial specimen were hybridized to the microarrays separately, we had to consider the possibility of systematic error leading to pairwise different expression values of the genes. For example, a positive shift of the glomerular data would make genes with truly similar expression appear to have higher expression in glomeruli. Therefore an adjustment of the data prior to any analysis is crucial to avoid the introduction of false positives and false negatives. Standard normalization methods are designed to remove rather minor technical variation evenly distributed across samples. As we have experienced a more pronounced effect on the two blocks of data we decided to normalize the sets separately and subsequently adjust the data as follows using a linear function.

While the underlying causes of this error type can be complex and hard to discern, a linear function provides a sensible starting point. In detail, this means to find an additive factor (difference of base expression) and a multiplicative one (difference in dynamic range) and apply this function to one of the datasets. By subsequent subtraction of the tubulointerstitial from the glomerular dataset we expected to find many values close to 0, the ones with positive result being our candidates for preferential glomerular expression.

To determine the factors, we calculated the mean of each probeset across all the samples in each condition, resulting in an aggregate expression profile for glomeruli and tubulointerstitium. After sorting both by ascending expression level of tubulointerstitium, we generated a line of best fit, its slope (multiplicative factor) and intercept (additive factor) for both datasets (glomeruli: y = 0.1641x+2.8035, tubulointerstitium: y = 0.1771x+1.5913). To adjust tubulointerstitium to glomeruli we multiplied each value by (0.1641/0.1771) = 0.9265951 and added (2.8035−1.5913) = 1.3022. To minimize effects of sorting, we repeated the same procedure after sorting by the glomeruli data and used the means of slope and intercept for the final adjustment function (y = 0.9392425x + 1.17905). Next we subtracted the corrected tubulointerstitial from glomerular expression values and calculated the standard deviation of this difference. The selection criterion for preferential glomerular expression was set as an expression difference exceeding twice this standard deviation (mean + 2*SD = 1.76) resulting in a dataset of 817 glomerular-enriched probesets. After removing unannotated probesets and redundant probesets a list of 677 glomerular-overrepresentated genes remained (Table S1).

### Digital Differential Display (DDD)

DDD, a bioinformatic tool available at the National Center for Biotechnology Information (www.ncbi.nlm.nih.gov/UniGene/info_ddd.html), analyzes the frequencies of cDNA and expressed sequence tag (EST) in expression libraries [34]. The DDD tool was used to compare human cDNA libraries of indicated organs or cells (i. e. normal adult brain, coronary artery smooth muscle cells, muscle and heart) with other organ cDNA libraries except testis as described previously [34].

### Analysis of biological processes and pathways

GOs and pathways were identified using a combination DAVID Bioinformatics database from the NIAID, NIH (Version 2010) (http://david.abcc.ncifcrf.gov/) [68], [69] and the KEGG database from the University of Kyoto (http://www.genome.jp/kegg/kegg2.html) [70], [71].

To obtain a more structured biological picture the functional annotation cluster analysis tool of DAVID was applied on REGGED. This analysis tool measures relationships among the annotation terms by using *Kappa*-statistics and is able to organize and cluster redundant and heterogeneous annotation terms into functional annotation groups which can provide a better understanding of the biological meaning for the given dataset [33].

For all DAVID analyses a p-value (Ease score) of 0.05 and fold enrichment of at least 1.5 was used as standard cut-off level. As gene reference background the Affymetrix HT human genome U133A available in DAVID was used [33], [69].

### Quantitative real-time RT-PCR

Reverse transcription and qRT-PCR was performed as reported earlier [63]. Pre-developed TaqMan reagents were used for human NRN1 (Hs00213192_m1), NPDC1 (Hs00209870_m1), ROBO1 (Hs00268049_m1) as well as the housekeeper genes (Applied Biosystems Europe, Rotkreuz, Switzerland). For the human ROBO2 (NM_002942), the following oligonucleotide primers (300 nmol/L) and probe (100 nmol/L) were used: sense primer 5′- ATTGAGGCTTTCAGCCAATCA-3′, antisense primer 5′- TGATCGCTCTGACCATGAATAAGT-3′; fluorescence labeled probe (FAM) 5′- TGAGCAACAGCTGGCAGACCGTG-3′. The expression of candidate genes was normalized by two reference genes, 18S rRNA and GAPDH, giving comparable results. The mRNA expression was analyzed by the delta delta Ct method for renal compartment analysis or standard curve quantification for disease-specific expression analysis.

### Western Blot

Robo1: Cultured human glomerular epithelial cells [72] were harvested with lysis buffer A (2% Triton, 150 mM NaCl, 100 mM HEPES, 2 mM EGTA, 2 mM Na_3_VO_4_, and Complete Protease Inhibitor Cocktail [Roche, Mannheim, Germany]). Extracted proteins were boiled in loading buffer for 5 min, resolved by 6% SDS-PAGE under reducing conditions, and transferred to an Immobilon-P membrane (Millipore, Eschborn, Germany).

Neuritin: Cultured human podocytes [72] were lysed with buffer B (150 mM NaCl, 1% Nonidet P-40, 0.5% DOC, 0.1% SDS, 50 mM Tris, pH 8.0, 100 mM DTT, 6M Urea and Complete Protease Inhibitor Cocktail [Roche, Mannheim, Germany]. Extracted proteins were heated for 15 min at 37°C, resolved by 16% Tricine-SDS-PAGE, containing 6M Urea [73] under reducing conditions, and transferred to an Immobilon-P membrane (Millipore, Eschborn, Germany).

Equal loading and transfer efficiency were verified by staining with 2% Ponceau S. Membranes were blocked overnight with Tris-buffered saline (TBS)/3% fat-free skim milk and then incubated with either a polyclonal rabbit anti-robo1 (Abcam, Cambridge, UK) diluted 1∶500 overnight at 4°C, or polyclonal rabbit anti-neuritin (Lifespan Biosciences, Seattle, WA, USA) diluted 1∶500 overnight at 4°C and rinsed with TBS that contained 0.1% Tween 20. For detection, a donkey anti-rabbit IgG ECL antibody, HRP conjugated (1∶10000, 1 hour at room temperature; GE Healthcare, Chalfont St. Giles, UK) and enhanced chemiluminescence substrate (PerkinElmer, Waltham, MA, USA) were used. Membranes were also probed with anti-beta-actin antibody (A 5316, 1∶5000, Sigma-Aldrich, Germany) as internal loading control.

### Immunofluorescence staining of human podocytes

Cells were fixed with 2% paraformaldehyde and 4% sucrose at room temperature for 10 min. The cells were then washed once with PBS, permeabilized with 0.3% Triton X-100 for 10 min and incubated with blocking solution (2% FCS, 2% BSA, 0.2% fish gelatin) for 30 min, before further incubation with a polyclonal rabbit anti-robo1 (Abcam, Cambridge, UK) or a polyclonal rabbit anti-neuritin antibody (Lifespan Biosciences, Seattle, WA, USA) for 1 h. For immunofluorescence detection, Alexa Fluor 488 goat anti-rabbit IgG secondary antibody (Invitrogen, Molecular Probes, Paisley, UK) were used at a dilution of 1∶200. For nuclear counterstaining, tissue sections were mounted with Vectashield with DAPI (Vector Laboratories, Burlinghame, CA, USA).

### Immunofluorescence staining of human control kidneys

Kidney tissue was obtained from the healthy pole of kidneys removed because of small and localized tumors. For immunofluorescence, the unfixed renal tissue was embedded in OCT (optimum cutting temperature cryoembedding matrix) (Tissue-Tek, Electron Microscopy Sciences, Società Italiana Chimici, Roma, Italy), snap-frozen in a mixture of isopentane and dry ice, and stored at −80°C. Indirect immunofluorescence was performed on 5-µm-thick tissue cryosections fixed in cold acetone with the primary antibodies rabbit anti-robo1 and rabbit anti-neuritin (both from Novus Biologicals, DBA Italia, Milan, Italy). Sections were incubated for 30 min with a fluorescent-labelled goat anti-rabbit secondary antibody (Alexafluor 488; Invitrogen, Milan, Italy) and nuclei counterstained by DAPI. Specificity of antibody labelling was demonstrated by the lack of staining after substituting proper control immunoglobulins (Rabbit primary antibody isotype control, Invitrogen) for the primary antibodies. Slides were mounted with Fluorsave aqueous mounting medium (Calbiochem, VWR International, Milan, Italy). Images were acquired by a Zeiss Axioscope 40FL microscope, equipped with AxioCam MRc5 digital videocamera and immunofluorescence apparatus (Carl Zeiss SpA, Arese, Mi, Italy), and recorded using AxioVision software 4.3.

### Statistics

Experimental data are given as mean ± SD. Statistical analysis was performed using Kruskall-Wallis and Mann-Whitney U tests (SPSS 17.0, SPSS Inc., Chicago, IL). P-values less than 0.05 were considered to indicate statistically significant differences.

## Supporting Information

Figure S1 DAVID Functional Annotation Cluster Analysis - genes involved in functional clusters. The terms involved in the respective functional annotation clusters are described in Table S5. Genes which are involved in the respective functional annotation cluster are shown in black.
(0.06 MB PDF)

Table S1 Renal glomerulus-enriched gene expression dataset (REGGED). 677 renal genes were identified to be enriched in the human glomerulus.
(0.72 MB DOC)

Table S2 List of known podocyte-, mesangial- and endothelial-specific markers as well as validated glomerular gene and protein expression data.
(0.35 MB DOC)

Table S3 Comparison of the 5 different approaches. 1 indicates the presence of the gene, 0 indicates the absence of the gene in the respective dataset. Sum: indicates in how many of the databases the respective gene is found.
(0.24 MB XLS)

Table S4 Prominent biological aspects found by DAVID analysis
(0.36 MB DOC)

Table S5 DAVID Functional Annotation Cluster Analysis
(0.13 MB DOC)

Table S6 KEGG pathways
(0.04 MB DOC)

Table S7 Neuron/brain-associated gene list
(0.40 MB DOC)

Table S8 Neuron/brain-associated genes present in the REGGED
(0.07 MB DOC)

Table S9 Smooth muscle cell associated gene list. Genes marked in bold font are present in REGGED.
(0.10 MB DOC)

Table S10 Muscle- and heart associated gene list. Genes marked in bold font are present in REGGED.
(0.16 MB DOC)

Table S11 Clinical and histological characteristics. Clinical and histological characteristics of patients and biopsies, respectively, with established diabetic nephropathy, focal segmental sclerosis and living donors analyzed by real-time RT-PCR (P) and oligonucleotide array based gene expression profiling (A) (for living donor). *  =  blood pressure before biopsy [mmHg].
(0.15 MB DOC)
