# Supplementary material for: Systematic Analysis of a Novel Human Renal Glomerulus-Enriched Gene Expression Dataset
Source: PLoS One. 2010 Jul 12;5(7):e11545. doi: 10.1371/journal.pone.0011545 (PMC2902524; doi:10.1371/journal.pone.0011545)
Supplement: Figure S1 — DAVID Functional Annotation Cluster Analysis - genes involved in functional clusters. The terms involved in the respective functional annotation clusters are described in Table S5. Genes which are involved in the respective functional annotation cluster are shown in black. (0.06 MB PDF) [file pone.0011545.s001.pdf]

Figure S1

Entrez Gene	Gene Symbol	AC1	AC2	AC3	AC4	AC5	AC6	AC7	AC8	AC9	AC10
29	ABR			■							
134	ADORA1			■	■		■			■	
375790	AGRN		■			■					
7840	ALMS1		■			■					
347902	AMIGO2			■						■	
301	ANXA1			■						■	
302	ANXA2	■									
304	ANXA2P2	■									
323	APBB2		■	■		■				■	■
324	APC		■	■	■	■			■	■	
23365	ARHGEF12			■							
50650	ARHGEF3			■							
440	ASNS			■						■	
472	ATM			■						■	
11041	B3GNT1		■			■					
633	BGN	■									
650	BMP2		■			■	■	■			
655	BMP7		■	■		■		■			
663	BNIP2			■						■	
2186	BPTF						■				
9044	BTAF1						■				
716	C1S					■					
857	CAV1	■					■		■		
972	CD74			■						■	
1027	CDKN1B			■	■		■		■		
1028	CDKN1C		■			■	■				
1952	CELSR2		■			■					
53405	CLIC5		■			■					
1281	COL3A1	■									
1285	COL4A3			■							
1490	CTGF	■									
8452	CUL3		■	■							
1627	DBN1					■					
10202	DHRS2			■						■	
9231	DLG5			■							
1756	DMD		■			■					
10049	DNAJB6			■			■				
667	DST		■			■			■		
1909	EDNRA	■					■	■			
1948	EFNB2	■									
9451	EIF2AK3			■			■				
2004	ELK3	■					■				

51705	EMCN	■									
51752	ERAP1	■									
2066	ERBB4				■						
2149	F2R			■	■					■	
2152	F3			■	■					■	
10116	FEM1B			■							
9638	FEZ1		■			■					
9637	FEZ2		■			■					
2246	FGF1	■									
2339	FNTA			■			■			■	
2296	FOXC1	■		■						■	
2534	FYN					■					
2619	GAS1		■	■		■					■
2690	GHR			■							
2697	GJA1		■			■					
2701	GJA4	■									
2898	GRIK2			■						■	
2934	GSN					■					
1839	HBEGF				■		■				
3087	HHEX						■				
3198	HOXA1		■			■					
3399	ID3			■		■	■				
3400	ID4					■	■				
3428	IFI16			■							
3479	IGF1			■	■	■		■		■	
3488	IGFBP5				■		■				
3551	IKBKB			■						■	
51447	IP6K2			■							
3685	ITGAV	■					■				
3690	ITGB3				■		■				
3720	JARID2						■				
3791	KDR	■			■			■			
3913	LAMB2		■			■					
3953	LEPR	■									
3956	LGALS1			■							
4005	LMO2	■									
4015	LOX	■									
1130	LYST			■							
23499	MACF1		■			■			■		
4131	MAP1B		■			■			■		
4134	MAP4								■		
4208	MEF2C			■			■			■	
4212	MEIS2						■				
4627	MYH9	■	■								
4643	MYO1E	■									
4664	NAB1					■	■				

138151	NACC2						■				
4692	NDN		■			■					
23114	NFASC		■			■					
11188	NISCH				■						
22861	NLRP1			■							
4853	NOTCH2			■						■	
4864	NPC1						■				
1728	NQO1			■							
83714	NRIP2						■				
4915	NTRK2	■	■			■					
4916	NTRK3					■					
56288	PARD3		■		■	■					
5159	PDGFRB				■						
10630	PDPN	■	■		■			■			
8682	PEA15			■						■	
5327	PLAT	■									
23129	PLXND1	■						■			
5584	PRKCI		■	■		■				■	
5699	PSMB10						■				
5696	PSMB8						■				
5698	PSMB9						■				
9444	QKI	■									
5925	RB1						■				■
23543	RBM9						■				
8434	RECK	■									
6091	ROBO1	■	■			■					
6093	ROCK1		■	■						■	
6237	RRAS				■						
6275	S100A4		■								
1901	S1PR1	■			■	■					
7869	SEMA3B		■			■					
10512	SEMA3C	■									
9037	SEMA5A	■	■			■		■			
10955	SERINC3			■							
5272	SERPINB9			■						■	
710	SERPING1						■				
51100	SH3GLB1			■						■	
357	SHROOM2		■								
150094	SIK1						■				
4091	SMAD6			■			■			■	■
6597	SMARCA4	■				■	■				■
6616	SNAP25		■			■					
6622	SNCA			■	■		■			■	
58533	SNX6						■				
6272	SORT1			■						■	
6672	SP100				■		■				

6750	SST			■	■						
6772	STAT1			■							
6812	STXBP1		■			■					
10579	TACC2					■			■		
6926	TBX3	■		■			■	■		■	
6943	TCF21	■					■	■			
6925	TCF4						■				
83439	TCF7L1						■				
7010	TEK				■						
7048	TGFBR2	■						■			
7072	TIA1			■			■				
7074	TIAM1			■		■					
7076	TIMP1						■				
7078	TIMP3			■			■				
25976	TIPARP	■									
7088	TLE1						■				
83941	TM2D1			■							
7187	TRAF3			■							
7188	TRAF5			■							
10100	TSPAN2					■					
64061	TSPYL2						■				
7329	UBE2I						■				
22846	VASH1				■						
7422	VEGFA	■	■	■	■	■		■		■	
7466	WFS1			■						■	
7490	WT1	■					■				
11059	WWP1						■				
51776	ZAK			■							
23318	ZCCHC11						■				
6935	ZEB1						■				
9839	ZEB2						■	■			
23414	ZFPM2	■					■				
7730	ZNF177						■				
23090	ZNF423						■				
