# Supplementary material for: Systematic Analysis of a Novel Human Renal Glomerulus-Enriched Gene Expression Dataset
Source: PLoS One. 2010 Jul 12;5(7):e11545. doi: 10.1371/journal.pone.0011545 (PMC2902524; doi:10.1371/journal.pone.0011545)
Supplement: Table S1 — Renal glomerulus-enriched gene expression dataset (REGGED). 677 renal genes were identified to be enriched in the human glomerulus. (0.72 MB DOC) [file pone.0011545.s002.doc]

**Table S1**

| **Probe Set ID** | **Entrez Gene** | **Gene Symbol** | **Gene Title** | **Mean G-Mean T** |
| --- | --- | --- | --- | --- |
| 202804_at | 4363 | ABCC1 | ATP-binding cassette, sub-family C (CFTR/MRP), member 1 | 1.84 |
| 212895_s_at | 29 | ABR | active BCR-related gene | 1.96 |
| 205481_at | 134 | ADORA1 | adenosine A1 receptor | 3.02 |
| 212285_s_at, 217419_x_at | 375790 | AGRN | agrin | 2.68, 2.82 |
| 205357_s_at | 185 | AGTR1 | angiotensin II receptor, type 1 | 2.07 |
| 221569_at | 54806 | AHI1 | Abelson helper integration site 1 | 1.92 |
| 211986_at | 79026 | AHNAK | AHNAK nucleoprotein | 2.85 |
| 209901_x_at, 213095_x_at, 215051_x_at | 199 | AIF1 | allograft inflammatory factor 1 | 3.35, 3.19, 3.48 |
| 212609_s_at | 10000 | AKT3 | Clones 23920 and 23921 mRNA sequence | 3.09 |
| 205583_s_at | 79868 | ALG13 | asparagine-linked glycosylation 13 homolog (S. cerevisiae) | 2.14 |
| 214221_at | 7840 | ALMS1 | Alstrom syndrome 1 | 1.93 |
| 222108_at | 347902 | AMIGO2 | adhesion molecule with Ig-like domain 2 | 4.61 |
| 201012_at | 301 | ANXA1 | annexin A1 | 3.25 |
| 201590_x_at, 210427_x_at, 213503_x_at | 302 | ANXA2 | annexin A2 | 2.98, 2.83, 3.16 |
| 208816_x_at | 304 | ANXA2P2 | annexin A2 pseudogene 2 | 2.63 |
| 212970_at, 212985_at, 40148_at | 323 | APBB2 | amyloid beta (A4) precursor protein-binding, family B, member 2 | 2.60, 3.56, 2.38 |
| 203525_s_at | 324 | APC | adenomatous polyposis coli | 2.66 |
| 213592_at | 187 | APLNR | apelin receptor | 2.07 |
| 201525_at | 347 | APOD | apolipoprotein D | 4.01 |
| 221087_s_at | 80833 | APOL3 | apolipoprotein L, 3 | 2.41 |
| 213618_at, 214102_at | 116984 | ARAP2 | ArfGAP with RhoGAP domain, ankyrin repeat and PH domain 2 | 2.47, 2.12 |
| 212738_at, 37577_at | 84986 | ARHGAP19 | Rho GTPase activating protein 19 | 2.85, 4.66 |
| 220382_s_at | 79822 | ARHGAP28 | Rho GTPase activating protein 28 | 2.07 |
| 201288_at | 397 | ARHGDIB | Rho GDP dissociation inhibitor (GDI) beta | 1.82 |
| 216620_s_at | 9639 | ARHGEF10 | Rho guanine nucleotide exchange factor (GEF) 10 | 2.49 |
| 201335_s_at | 23365 | ARHGEF12 | Rho guanine nucleotide exchange factor (GEF) 12 | 2.93 |
| 218501_at | 50650 | ARHGEF3 | Rho guanine nucleotide exchange factor (GEF) 3 | 3.3 |
| 218694_at | 51309 | ARMCX1 | armadillo repeat containing, X-linked 1 | 1.78 |
| 205047_s_at | 440 | ASNS | asparagine synthetase | 2.16 |
| 209135_at | 444 | ASPH | aspartate beta-hydroxylase | 2.15 |
| 212672_at | 472 | ATM | ataxia telangiectasia mutated | 1.94 |
| 214255_at | 57194 | ATP10A | ATPase, class V, type 10A | 3.48 |
| 212536_at | 23200 | ATP11B | ATPase, class VI, type 11B | 2.07 |
| 212297_at | 79572 | ATP13A3 | ATPase type 13A3 | 1.84 |
| 203296_s_at | 477 | ATP1A2 | ATPase, Na+/K+ transporting, alpha 2 (+) polypeptide | 2.2 |
| 212361_s_at | 488 | ATP2A2 | ATPase, Ca++ transporting, cardiac muscle, slow twitch 2 | 2.1 |
| 213106_at | 10396 | ATP8A1 | ATPase, aminophospholipid transporter (APLT), class I, type 8A, member 1 | 2.06 |
| 212062_at | 10079 | ATP9A | ATPase, class II, type 9A | 3.23 |
| 202686_s_at | 558 | AXL | AXL receptor tyrosine kinase | 2.69 |
| 210121_at, 217452_s_at | 8707 | B3GALT2 | UDP-Gal:betaGlcNAc beta 1,3-galactosyltransferase, polypeptide 2 | 3.21, 2.73 |
| 203188_at | 11041 | B3GNT1 | UDP-GlcNAc:betaGal beta-1,3-N-acetylglucosaminyltransferase 1 | 1.96 |
| 217904_s_at | 23621 | BACE1 | beta-site APP-cleaving enzyme 1 | 2.05 |
| 221234_s_at | 60468 | BACH2 | BTB and CNC homology 1, basic leucine zipper transcription factor 2 | 1.9 |
| 203304_at | 25805 | BAMBI | BMP and activin membrane-bound inhibitor homolog (Xenopus laevis) | 2.03 |
| 219667_s_at | 55024 | BANK1 | B-cell scaffold protein with ankyrin repeats 1 | 1.77 |
| 200041_s_at | 7919 | BAT1 | HLA-B associated transcript 1 | 2.15 |
| 203080_s_at | 29994 | BAZ2B | bromodomain adjacent to zinc finger domain, 2B | 2.07 |
| 40093_at | 4059 | BCAM | basal cell adhesion molecule (Lutheran blood group) | 1.84 |
| 218332_at | 55859 | BEX1 | brain expressed, X-linked 1 | 1.96 |
| 215440_s_at | 56271 | BEX4 | brain expressed, X-linked 4 | 2.27 |
| 201261_x_at, 213905_x_at | 633 | BGN | biglycan | 2.58, 2.85 |
| 205289_at | 650 | BMP2 | bone morphogenetic protein 2 | 3.36 |
| 209590_at | 655 | BMP7 | bone morphogenetic protein 7 | 3.49 |
| 209308_s_at | 663 | BNIP2 | BCL2/adenovirus E1B 19kDa interacting protein 2 | 2.68 |
| 207186_s_at | 2186 | BPTF | bromodomain PHD finger transcription factor | 1.77 |
| 201641_at | 684 | BST2 | bone marrow stromal cell antigen 2 | 3.73 |
| 209430_at | 9044 | BTAF1 | BTAF1 RNA polymerase II, B-TFIID transcription factor-associated, 170kDa (Mot1 homolog, S. cerevisiae) | 1.8 |
| 217945_at | 53339 | BTBD1 | BTB (POZ) domain containing 1 | 1.78 |
| 202946_s_at | 22903 | BTBD3 | BTB (POZ) domain containing 3 | 2.24 |
| 218820_at | 56967 | C14orf132 | chromosome 14 open reading frame 132 | 2.76 |
| 219563_at | 79686 | C14orf139 | chromosome 14 open reading frame 139 | 2.54 |
| 217957_at | 29105 | C16orf80 | chromosome 16 open reading frame 80 | 2.05 |
| 221988_at | 79086 | C19orf42 | chromosome 19 open reading frame 42 | 1.88 |
| 221272_s_at | 81563 | C1orf21 | chromosome 1 open reading frame 21 | 2.19 |
| 203429_s_at | 51430 | C1orf9 | chromosome 1 open reading frame 9 | 1.85 |
| 212067_s_at | 715 | C1R | complement component 1, r subcomponent | 2.84 |
| 208747_s_at | 716 | C1S | complement component 1, s subcomponent | 2.99 |
| 206656_s_at | 57136 | C20orf3 | chromosome 20 open reading frame 3 | 2.1 |
| 219747_at | 79625 | C4orf31 | chromosome 4 open reading frame 31 | 4.53 |
| 218187_s_at | 65265 | C8orf33 | chromosome 8 open reading frame 33 | 2 |
| 47530_at | 51531 | C9orf156 | chromosome 9 open reading frame 156 | 1.8 |
| 220889_s_at | 56934 | CA10 | carbonic anhydrase X | 4.15 |
| 213714_at | 783 | CACNB2 | calcium channel, voltage-dependent, beta 2 subunit | 2.67 |
| 212765_at, 217196_s_at | 23271 | CAMSAP1L1 | calmodulin regulated spectrin-associated protein 1-like 1 | 2.54, 2.12 |
| 213547_at | 23066 | CAND2 | cullin-associated and neddylation-dissociated 2 (putative) | 2.64 |
| 207842_s_at | 22794 | CASC3 | cancer susceptibility candidate 3 | 1.93 |
| 212586_at | 831 | CAST | calpastatin | 1.81 |
| 212097_at | 857 | CAV1 | caveolin 1, caveolae protein, 22kDa | 2.01 |
| 209682_at | 868 | CBLB | Cas-Br-M (murine) ecotropic retroviral transforming sequence b | 3.55 |
| 212914_at | 23492 | CBX7 | chromobox homolog 7 | 2.33 |
| 213644_at | 201134 | CCDC46 | coiled-coil domain containing 46 | 2.29 |
| 218545_at | 55297 | CCDC91 | coiled-coil domain containing 91 | 3.16 |
| 213743_at | 905 | CCNT2 | cyclin T2 | 2.07 |
| 214152_at | 9236 | CCPG1 | cell cycle progression 1 | 2 |
| 209582_s_at, 209583_s_at | 4345 | CD200 | CD200 molecule | 2.11, 3.48 |
| 203799_at | 9936 | CD302 | CD302 molecule | 1.85 |
| 211075_s_at, 213857_s_at | 961 | CD47 | CD47 molecule | 2.17, 1.97 |
| 201925_s_at | 1604 | CD55 | CD55 molecule, decay accelerating factor for complement (Cromer blood group) | 2.61 |
| 209619_at | 972 | CD74 | CD74 molecule, major histocompatibility complex, class II invariant chain | 1.87 |
| 200675_at | 975 | CD81 | CD81 molecule | 2.26 |
| 205288_at | 8556 | CDC14A | CDC14 cell division cycle 14 homolog A (S. cerevisiae) | 3.42 |
| 201853_s_at | 994 | CDC25B | cell division cycle 25 homolog B (S. pombe) | 2.14 |
| 214464_at | 8476 | CDC42BPA | CDC42 binding protein kinase alpha (DMPK-like) | 2.13 |
| 209286_at, 209287_s_at, 209288_s_at | 10602 | CDC42EP3 | CDC42 effector protein (Rho GTPase binding) 3 | 1.93, 2.86, 4.27 |
| 209112_at | 1027 | CDKN1B | cyclin-dependent kinase inhibitor 1B (p27, Kip1) | 2.19 |
| 213182_x_at, 213348_at, 216894_x_at, 219534_x_at | 1028 | CDKN1C | cyclin-dependent kinase inhibitor 1C (p57, Kip2) | 2.48, 2.58, 3.22, 3.27 |
| 36499_at | 1952 | CELSR2 | cadherin, EGF LAG seven-pass G-type receptor 2 (flamingo homolog, Drosophila) | 1.99 |
| 209662_at | 1070 | CETN3 | centrin, EF-hand protein, 3 (CDC31 homolog, yeast) | 2.21 |
| 213800_at | 3075 | CFH | complement factor H | 2.27 |
| 206910_x_at | 3080 | CFHR2 | complement factor H-related 2 | 1.8 |
| 218642_s_at | 79145 | CHCHD7 | coiled-coil-helix-coiled-coil-helix domain containing 7 | 1.81 |
| 208806_at | 1107 | CHD3 | chromodomain helicase DNA binding protein 3 | 2.09 |
| 209395_at, 209396_s_at | 1116 | CHI3L1 | chitinase 3-like 1 (cartilage glycoprotein-39) | 5.90, 5.79 |
| 218927_s_at | 55501 | CHST12 | carbohydrate (chondroitin 4) sulfotransferase 12 | 2.05 |
| 203921_at | 9435 | CHST2 | carbohydrate (N-acetylglucosamine-6-O) sulfotransferase 2 | 2.33 |
| 200999_s_at | 10970 | CKAP4 | cytoskeleton-associated protein 4 | 2.47 |
| 204482_at | 7122 | CLDN5 | claudin 5 | 3.61 |
| 213415_at | 1193 | CLIC2 | chloride intracellular channel 2 | 2.03 |
| 213317_at, 217628_at | 53405 | CLIC5 | chloride intracellular channel 5 | 2.65, 2.87 |
| 212358_at | 25999 | CLIP3 | CAP-GLY domain containing linker protein 3 | 2 |
| 205518_s_at, 210571_s_at | 8418 | CMAH | cytidine monophosphate-N-acetylneuraminic acid hydroxylase (CMP-N-acetylneuraminate monooxygenase) pseudogene | 2.27, 2.04 |
| 201852_x_at, 211161_s_at, 215076_s_at | 1281 | COL3A1 | collagen, type III, alpha 1 | 1.83, 2.47, 2.07 |
| 222073_at | 1285 | COL4A3 | collagen, type IV, alpha 3 (Goodpasture antigen) | 3.27 |
| 214602_at | 1286 | COL4A4 | collagen, type IV, alpha 4 | 1.93 |
| 213110_s_at | 1287 | COL4A5 | collagen, type IV, alpha 5 | 4.04 |
| 209789_at | 10391 | CORO2B | coronin, actin binding protein, 2B | 3.17 |
| 206244_at, 217484_at, 217552_x_at | 1378 | CR1 | complement component (3b/4b) receptor 1 (Knops blood group) | 1.79, 2.01, 3.76 |
| 203368_at | 78987 | CRELD1 | cysteine-rich with EGF-like domains 1 | 1.92 |
| 209967_s_at | 1390 | CREM | cAMP responsive element modulator | 1.83 |
| 205984_at | 1393 | CRHBP | corticotropin releasing hormone binding protein | 3.8 |
| 202551_s_at, 202552_s_at | 51232 | CRIM1 | cysteine rich transmembrane BMP regulator 1 (chordin-like) | 1.91, 4.06 |
| 208978_at | 1397 | CRIP2 | cysteine-rich protein 2 | 2.28 |
| 203804_s_at | 51747 | CROP | cisplatin resistance-associated overexpressed protein | 1.78 |
| 218871_x_at, 222235_s_at | 55454 | CSGALNACT2 | chondroitin sulfate N-acetylgalactosaminyltransferase 2 | 2.28, 2.43 |
| 208774_at | 1453 | CSNK1D | casein kinase 1, delta | 1.96 |
| 221260_s_at | 81566 | CSRNP2 | cysteine-serine-rich nuclear protein 2 | 1.98 |
| 200621_at | 1465 | CSRP1 | cysteine and glycine-rich protein 1 | 2.24 |
| 201906_s_at | 10217 | CTDSPL | CTD (carboxy-terminal domain, RNA polymerase II, polypeptide A) small phosphatase-like | 2.84 |
| 209101_at | 1490 | CTGF | connective tissue growth factor | 3.83 |
| 201370_s_at | 8452 | CUL3 | cullin 3 | 2.1 |
| 202436_s_at, 202437_s_at | 1545 | CYP1B1 | cytochrome P450, family 1, subfamily B, polypeptide 1 | 1.92, 3.03 |
| 219825_at | 56603 | CYP26B1 | cytochrome P450, family 26, subfamily B, polypeptide 1 | 1.99 |
| 212793_at | 23500 | DAAM2 | dishevelled associated activator of morphogenesis 2 | 1.93 |
| 205417_s_at | 1605 | DAG1 | dystroglycan 1 (dystrophin-associated glycoprotein 1) | 2.8 |
| 202806_at | 1627 | DBN1 | drebrin 1 | 2.51 |
| 201893_x_at, 209335_at, 211813_x_at, 211896_s_at | 1634 | DCN | decorin | 2.67, 3.72, 3.07, 3.68 |
| 201572_x_at | 1635 | DCTD | dCMP deaminase | 1.94 |
| 212690_at | 23259 | DDHD2 | DDHD domain containing 2 | 1.83 |
| 214788_x_at | 23109 | DDN | dendrin | 4.06 |
| 214079_at | 10202 | DHRS2 | dehydrogenase/reductase (SDR family) member 2 | 1.83 |
| 213661_at | 25891 | DKFZP586H2123 | regeneration associated muscle protease | 3.68 |
| 202196_s_at, 214247_s_at | 27122 | DKK3 | dickkopf homolog 3 (Xenopus laevis) | 2.17, 3.03 |
| 201681_s_at | 9231 | DLG5 | discs, large homolog 5 (Drosophila) | 2.14 |
| 203881_s_at | 1756 | DMD | dystrophin | 2.65 |
| 209015_s_at | 10049 | DNAJB6 | DnaJ (Hsp40) homolog, subfamily B, member 6 | 1.77 |
| 203187_at | 1793 | DOCK1 | dedicator of cytokinesis 1 | 1.81 |
| 205003_at | 9732 | DOCK4 | dedicator of cytokinesis 4 | 2.54 |
| 212538_at | 23348 | DOCK9 | dedicator of cytokinesis 9 | 2.48 |
| 207789_s_at | 1804 | DPP6 | dipeptidyl-peptidase 6 | 3.49 |
| 200762_at | 1808 | DPYSL2 | dihydropyrimidinase-like 2 | 1.88 |
| 201430_s_at, 201431_s_at | 1809 | DPYSL3 | dihydropyrimidinase-like 3 | 2.13, 3.10 |
| 212254_s_at, 215016_x_at | 667 | DST | dystonin | 2.61, 2.68 |
| 203367_at | 11072 | DUSP14 | dual specificity phosphatase 14 | 1.85 |
| 205348_s_at | 1780 | DYNC1I1 | dynein, cytoplasmic 1, intermediate chain 1 | 3.31 |
| 204464_s_at | 1909 | EDNRA | endothelin receptor type A | 2.63 |
| 201842_s_at, 201843_s_at | 2202 | EFEMP1 | EGF-containing fibulin-like extracellular matrix protein 1 | 2.56, 2.91 |
| 209356_x_at | 30008 | EFEMP2 | EGF-containing fibulin-like extracellular matrix protein 2 | 1.93 |
| 219833_s_at | 114327 | EFHC1 | EF-hand domain (C-terminal) containing 1 | 1.77 |
| 202668_at | 1948 | EFNB2 | ephrin-B2 | 3.64 |
| 206115_at | 1960 | EGR3 | early growth response 3 | 2.23 |
| 221870_at | 30846 | EHD2 | EH-domain containing 2 | 2.04 |
| 218935_at | 30845 | EHD3 | EH-domain containing 3 | 4.28 |
| 209536_s_at | 30844 | EHD4 | EH-domain containing 4 | 2.19 |
| 213294_at | 5610 | EIF2AK2 | eukaryotic translation initiation factor 2-alpha kinase 2 | 2.06 |
| 218696_at | 9451 | EIF2AK3 | eukaryotic translation initiation factor 2-alpha kinase 3 | 2.4 |
| 31845_at | 2000 | ELF4 | E74-like factor 4 (ets domain transcription factor) | 2.44 |
| 221773_at | 2004 | ELK3 | ELK3, ETS-domain protein (SRF accessory protein 2), mRNA (cDNA clone MGC:13551 IMAGE:4287696) | 1.98 |
| 55692_at | 63916 | ELMO2 | engulfment and cell motility 2 | 2.43 |
| 219436_s_at | 51705 | EMCN | endomucin | 1.95 |
| 204797_s_at | 2009 | EML1 | echinoderm microtubule associated protein like 1 | 2.01 |
| 212573_at | 23052 | ENDOD1 | endonuclease domain containing 1 | 2.07 |
| 204161_s_at | 22875 | ENPP4 | ectonucleotide pyrophosphatase/phosphodiesterase 4 (putative function) | 1.82 |
| 221486_at | 2029 | ENSA | endosulfine alpha | 2.11 |
| 204076_at | 9583 | ENTPD4 | ectonucleoside triphosphate diphosphohydrolase 4 | 2.01 |
| 210385_s_at, 214012_at | 51752 | ERAP1 | endoplasmic reticulum aminopeptidase 1 | 1.77, 1.96 |
| 214053_at | 2066 | ERBB4 | v-erb-a erythroblastic leukemia viral oncogene homolog 4 (avian) | 1.84 |
| 213541_s_at | 2078 | ERG | v-ets erythroblastosis virus E26 oncogene homolog (avian) | 2.26 |
| 204034_at | 23474 | ETHE1 | ethylmalonic encephalopathy 1 | 1.95 |
| 201328_at | 2114 | ETS2 | v-ets erythroblastosis virus E26 oncogene homolog 2 (avian) | 2.38 |
| 209717_at | 7813 | EVI5 | ecotropic viral integration site 5 | 1.82 |
| 217838_s_at | 51466 | EVL | Enah/Vasp-like | 2.72 |
| 203989_x_at | 2149 | F2R | coagulation factor II (thrombin) receptor | 3.96 |
| 204363_at | 2152 | F3 | coagulation factor III (thromboplastin, tissue factor) | 2.9 |
| 204714_s_at | 2153 | F5 | coagulation factor V (proaccelerin, labile factor) | 3.37 |
| 213455_at | 92689 | FAM114A1 | family with sequence similarity 114, member A1 | 2.19 |
| 212981_s_at | 9747 | FAM115A | family with sequence similarity 115, member A | 1.83 |
| 218518_at | 51306 | FAM13B1 | family with sequence similarity 13, member B1 | 2.32 |
| 51158_at | 400451 | FAM174B | family with sequence similarity 174, member B | 2.84 |
| 202916_s_at | 9917 | FAM20B | family with sequence similarity 20, member B | 2.46 |
| 202771_at | 9780 | FAM38A | family with sequence similarity 38, member A | 3 |
| 218029_at, 45749_at | 79567 | FAM65A | family with sequence similarity 65, member A | 3.36, 2.61 |
| 209829_at | 9750 | FAM65B | family with sequence similarity 65, member B | 2.52 |
| 212333_at | 25940 | FAM98A | family with sequence similarity 98, member A | 1.97 |
| 218397_at | 55120 | FANCL | Fanconi anemia, complementation group L | 2.61 |
| 201579_at | 2195 | FAT1 | FAT tumor suppressor homolog 1 (Drosophila) | 2.36 |
| 219427_at | 79633 | FAT4 | FAT tumor suppressor homolog 4 (Drosophila) | 2.05 |
| 213249_at | 23194 | FBXL7 | F-box and leucine-rich repeat protein 7 | 2.01 |
| 203620_s_at | 9873 | FCHSD2 | FCH and double SH3 domains 2 | 2.66 |
| 205866_at | 8547 | FCN3 | ficolin (collagen/fibrinogen domain containing) 3 (Hakata antigen) | 3.55 |
| 212367_at | 10116 | FEM1B | fem-1 homolog b (C. elegans) | 2.12 |
| 209210_s_at | 10979 | FERMT2 | fermitin family homolog 2 (Drosophila) | 1.84 |
| 203562_at | 9638 | FEZ1 | fasciculation and elongation protein zeta 1 (zygin I) | 2.34 |
| 215000_s_at | 9637 | FEZ2 | fasciculation and elongation protein zeta 2 (zygin II) | 1.82 |
| 205117_at, 208240_s_at | 2246 | FGF1 | fibroblast growth factor 1 (acidic) | 3.61, 2.89 |
| 201540_at, 210298_x_at, 210299_s_at | 2273 | FHL1 | four and a half LIM domains 1 | 3.20, 2.00, 2.95 |
| 58780_s_at | 55701 | FLJ10357 | hypothetical protein FLJ10357 | 2.4 |
| 218454_at | 79887 | FLJ22662 | hypothetical protein FLJ22662 | 2.44 |
| 204359_at | 23768 | FLRT2 | fibronectin leucine rich transmembrane protein 2 | 2.8 |
| 219250_s_at | 23767 | FLRT3 | fibronectin leucine rich transmembrane protein 3 | 1.99 |
| 212288_at | 23048 | FNBP1 | formin binding protein 1 | 3.75 |
| 215017_s_at | 54874 | FNBP1L | formin binding protein 1-like | 2.36 |
| 200090_at | 2339 | FNTA | farnesyltransferase, CAAX box, alpha | 1.92 |
| 213260_at | 2296 | FOXC1 | forkhead box C1 | 3.25 |
| 206307_s_at | 2297 | FOXD1 | forkhead box D1 | 2.84 |
| 204072_s_at, 214319_at | 10129 | FRY | furry homolog (Drosophila) | 4.66, 3.26 |
| 203697_at, 203698_s_at | 2487 | FRZB | frizzled-related protein | 2.06, 2.05 |
| 208782_at | 11167 | FSTL1 | follistatin-like 1 | 2.6 |
| 210105_s_at, 212486_s_at | 2534 | FYN | FYN oncogene related to SRC, FGR, YES | 2.30, 2.95 |
| 210220_at | 2535 | FZD2 | frizzled homolog 2 (Drosophila) | 3.69 |
| 203706_s_at | 8324 | FZD7 | frizzled homolog 7 (Drosophila) | 2.05 |
| 203853_s_at | 9846 | GAB2 | GRB2-associated binding protein 2 | 2.37 |
| 204417_at | 2581 | GALC | galactosylceramidase | 2.33 |
| 204457_s_at | 2619 | GAS1 | growth arrest-specific 1 | 3.4 |
| 205498_at | 2690 | GHR | growth hormone receptor | 2.48 |
| 219243_at | 55303 | GIMAP4 | GTPase, IMAP family member 4 | 2.34 |
| 219777_at | 474344 | GIMAP6 | GTPase, IMAP family member 6 | 3.82 |
| 201667_at | 2697 | GJA1 | gap junction protein, alpha 1, 43kDa | 3.43 |
| 40687_at | 2701 | GJA4 | gap junction protein, alpha 4, 37kDa | 2.01 |
| 209883_at | 23127 | GLT25D2 | glycosyltransferase 25 domain containing 2 | 2.27 |
| 215001_s_at | 2752 | GLUL | glutamate-ammonia ligase (glutamine synthetase) | 1.99 |
| 204875_s_at, 214106_s_at | 2762 | GMDS | GDP-mannose 4,6-dehydratase | 3.24, 3.21 |
| 204115_at | 2791 | GNG11 | guanine nucleotide binding protein (G protein), gamma 11 | 2.12 |
| 212959_s_at | 79158 | GNPTAB | N-acetylglucosamine-1-phosphate transferase, alpha and beta subunits | 2.62 |
| 201056_at | 2804 | GOLGB1 | golgin B1, golgi integral membrane protein | 2 |
| 204324_s_at | 27333 | GOLIM4 | golgi integral membrane protein 4 | 2.18 |
| 218692_at | 55638 | GOLSYN | Golgi-localized protein | 2.44 |
| 201141_at | 10457 | GPNMB | glycoprotein (transmembrane) nmb | 2.8 |
| 203108_at | 9052 | GPRC5A | G protein-coupled receptor, family C, group 5, member A | 2.79 |
| 213845_at | 2898 | GRIK2 | glutamate receptor, ionotropic, kainate 2 | 2.68 |
| 204396_s_at | 2869 | GRK5 | G protein-coupled receptor kinase 5 | 3.68 |
| 200696_s_at | 2934 | GSN | gelsolin (amyloidosis, Finnish type) | 2.1 |
| 203817_at | 2983 | GUCY1B3 | guanylate cyclase 1, soluble, beta 3 | 2.04 |
| 204237_at | 51454 | GULP1 | GULP, engulfment adaptor PTB domain containing 1 | 2.73 |
| 211275_s_at | 2992 | GYG1 | glycogenin 1 | 2.24 |
| 208886_at | 3005 | H1F0 | H1 histone family, member 0 | 2.48 |
| 204805_s_at | 8971 | H1FX | H1 histone family, member X | 2.6 |
| 220936_s_at | 55766 | H2AFJ | H2A histone family, member J | 3.28 |
| 208579_x_at | 54145 | H2BFS | H2B histone family, member S | 2.46 |
| 203821_at, 38037_at | 1839 | HBEGF | heparin-binding EGF-like growth factor | 3.45, 2.97 |
| 213069_at | 57493 | HEG1 | HEG homolog 1 (zebrafish) | 2.18 |
| 204689_at | 3087 | HHEX | hematopoietically expressed homeobox | 2.4 |
| 209806_at | 85236 | HIST1H2BK | histone cluster 1, H2bk | 3.42 |
| 212642_s_at | 3097 | HIVEP2 | human immunodeficiency virus type I enhancer binding protein 2 | 1.84 |
| 202934_at | 3099 | HK2 | hexokinase 2 | 2.36 |
| 211990_at | 3113 | HLA-DPA1 | major histocompatibility complex, class II, DP alpha 1 | 1.8 |
| 200904_at | 3133 | HLA-E | major histocompatibility complex, class I, E | 2.02 |
| 211929_at | 220988 | HNRNPA3 | heterogeneous nuclear ribonucleoprotein A3 | 2.04 |
| 214639_s_at | 3198 | HOXA1 | homeobox A1 | 1.79 |
| 204544_at | 11234 | HPS5 | Hermansky-Pudlak syndrome 5 | 2.64 |
| 205579_at | 3269 | HRH1 | histamine receptor H1 | 1.93 |
| 219985_at | 9955 | HS3ST3A1 | heparan sulfate (glucosamine) 3-O-sulfotransferase 3A1 | 3.92 |
| 214434_at | 259217 | HSPA12A | heat shock 70kDa protein 12A | 2.54 |
| 211538_s_at | 3306 | HSPA2 | heat shock 70kDa protein 2 | 2.27 |
| 221667_s_at | 26353 | HSPB8 | heat shock 22kDa protein 8 | 2.66 |
| 201185_at | 5654 | HTRA1 | HtrA serine peptidase 1 | 3.74 |
| 204683_at, 213620_s_at | 3384 | ICAM2 | intercellular adhesion molecule 2 | 2.14, 3.55 |
| 207826_s_at | 3399 | ID3 | inhibitor of DNA binding 3, dominant negative helix-loop-helix protein | 2.62 |
| 209292_at | 3400 | ID4 | Id-related helix-loop-helix protein Id4 | 2.62 |
| 206332_s_at, 208966_x_at | 3428 | IFI16 | interferon, gamma-inducible protein 16 | 2.09, 2.65 |
| 202411_at | 3429 | IFI27 | interferon, alpha-inducible protein 27 | 2.26 |
| 214453_s_at | 10561 | IFI44 | interferon-induced protein 44 | 1.89 |
| 201601_x_at | 8519 | IFITM1 | interferon induced transmembrane protein 1 (9-27) | 1.97 |
| 201315_x_at | 10581 | IFITM2 | interferon induced transmembrane protein 2 (1-8D) | 2.61 |
| 212203_x_at | 10410 | IFITM3 | interferon induced transmembrane protein 3 (1-8U) | 1.81 |
| 209541_at | 3479 | IGF1 | insulin-like growth factor 1 (somatomedin C) | 4.2 |
| 202718_at | 3485 | IGFBP2 | insulin-like growth factor binding protein 2, 36kDa | 3.53 |
| 203424_s_at, 211959_at | 3488 | IGFBP5 | insulin-like growth factor binding protein 5 | 1.90, 2.73 |
| 209341_s_at | 3551 | IKBKB | inhibitor of kappa light polypeptide gene enhancer in B-cells, kinase beta | 1.88 |
| 201887_at | 3597 | IL13RA1 | interleukin 13 receptor, alpha 1 | 1.89 |
| 206172_at | 3598 | IL13RA2 | interleukin 13 receptor, alpha 2 | 4.14 |
| 205227_at | 3556 | IL1RAP | interleukin 1 receptor accessory protein | 4.33 |
| 207526_s_at | 9173 | IL1RL1 | interleukin 1 receptor-like 1 | 2.66 |
| 218637_at | 55364 | IMPACT | Impact homolog (mouse) | 1.97 |
| 218192_at | 51447 | IP6K2 | inositol hexakisphosphate kinase 2 | 1.96 |
| 200791_s_at | 8826 | IQGAP1 | IQ motif containing GTPase activating protein 1 | 1.82 |
| 203474_at | 10788 | IQGAP2 | IQ motif containing GTPase activating protein 2 | 2.06 |
| 203906_at | 9922 | IQSEC1 | IQ motif and Sec7 domain 1 | 1.93 |
| 201474_s_at | 3675 | ITGA3 | integrin, alpha 3 (antigen CD49C, alpha 3 subunit of VLA-3 receptor) | 2.92 |
| 202351_at | 3685 | ITGAV | integrin, alpha V (vitronectin receptor, alpha polypeptide, antigen CD51) | 2.82 |
| 204627_s_at, 216261_at | 3690 | ITGB3 | integrin, beta 3 (platelet glycoprotein IIIa, antigen CD61) | 1.77, 2.26 |
| 201124_at, 201125_s_at | 3693 | ITGB5 | integrin, beta 5 | 1.90, 2.35 |
| 219064_at | 80760 | ITIH5 | inter-alpha (globulin) inhibitor H5 | 4.15 |
| 202746_at | 9452 | ITM2A | integral membrane protein 2A | 2.46 |
| 203298_s_at | 3720 | JARID2 | jumonji, AT rich interactive domain 2 | 1.85 |
| 213005_s_at | 23189 | KANK1 | KN motif and ankyrin repeat domains 1 | 2.26 |
| 218418_s_at | 25959 | KANK2 | KN motif and ankyrin repeat domains 2 | 1.77 |
| 213715_s_at | 256949 | KANK3 | KN motif and ankyrin repeat domains 3 | 2.09 |
| 203845_at | 8850 | KAT2B | K(lysine) acetyltransferase 2B | 2.29 |
| 204301_at | 9920 | KBTBD11 | kelch repeat and BTB (POZ) domain containing 11 | 2.13 |
| 212447_at | 25948 | KBTBD2 | kelch repeat and BTB (POZ) domain containing 2 | 2.12 |
| 213832_at | 3752 | KCND3 | potassium voltage-gated channel, Shal-related subfamily, member 3 | 2.67 |
| 210036_s_at | 3757 | KCNH2 | potassium voltage-gated channel, subfamily H (eag-related), member 2 | 2.25 |
| 34858_at | 23510 | KCTD2 | potassium channel tetramerisation domain containing 2 | 2.03 |
| 204017_at | 11015 | KDELR3 | KDEL (Lys-Asp-Glu-Leu) endoplasmic reticulum protein retention receptor 3 | 3.21 |
| 203934_at | 3791 | KDR | kinase insert domain receptor (a type III receptor tyrosine kinase) | 3.11 |
| 203143_s_at, 203144_s_at | 9674 | KIAA0040 | KIAA0040 | 2.46, 2.26 |
| 212733_at | 9711 | KIAA0226 | KIAA0226 | 1.88 |
| 212441_at | 9778 | KIAA0232 | KIAA0232 | 2.01 |
| 203288_at | 9710 | KIAA0355 | KIAA0355 | 1.92 |
| 212427_at | 23392 | KIAA0368 | KIAA0368 | 2.41 |
| 209379_s_at | 54462 | KIAA1128 | KIAA1128 | 2.17 |
| 213316_at | 57608 | KIAA1462 | KIAA1462 | 2.14 |
| 222139_at | 57612 | KIAA1466 | KIAA1466 gene | 2.12 |
| 212877_at, 212878_s_at, 213656_s_at | 3831 | KLC1 | kinesin light chain 1 | 2.51, 2.06, 4.27 |
| 204733_at | 5653 | KLK6 | kallikrein-related peptidase 6 | 4.16 |
| 205778_at | 5650 | KLK7 | kallikrein-related peptidase 7 | 3.7 |
| 216264_s_at | 3913 | LAMB2 | laminin, beta 2 (laminin S) | 2.63 |
| 215543_s_at | 9215 | LARGE | like-glycosyltransferase | 2.02 |
| 212446_s_at | 253782 | LASS6 | LAG1 homolog, ceramide synthase 6 | 3.9 |
| 221011_s_at | 81606 | LBH | limb bud and heart development homolog (mouse) | 1.88 |
| 206481_s_at | 9079 | LDB2 | LIM domain binding 2 | 2.61 |
| 209894_at | 3953 | LEPR | leptin receptor | 2.7 |
| 218717_s_at | 55214 | LEPREL1 | leprecan-like 1 | 2.03 |
| 201105_at | 3956 | LGALS1 | lectin, galactoside-binding, soluble, 1 | 2.89 |
| 200923_at | 3959 | LGALS3BP | lectin, galactoside-binding, soluble, 3 binding protein | 1.87 |
| 208933_s_at, 208935_s_at, 208936_x_at | 3964 | LGALS8 | lectin, galactoside-binding, soluble, 8 | 3.48, 2.10, 2.27 |
| 218656_s_at | 10186 | LHFP | lipoma HMGIC fusion partner | 2.79 |
| 202193_at | 3985 | LIMK2 | LIM domain kinase 2 | 2.57 |
| 204249_s_at | 4005 | LMO2 | LIM domain only 2 (rhombotin-like 1) | 2.29 |
| 212098_at | 151162 | LOC151162 | hypothetical LOC151162 | 4.05 |
| 204298_s_at, 215446_s_at | 4015 | LOX | lysyl oxidase | 3.87, 5.37 |
| 203570_at | 4016 | LOXL1 | lysyl oxidase-like 1 | 2.65 |
| 203549_s_at | 4023 | LPL | lipoprotein lipase | 3.15 |
| 218816_at | 55227 | LRRC1 | leucine rich repeat containing 1 | 1.87 |
| 219949_at | 79442 | LRRC2 | leucine rich repeat containing 2 | 4.26 |
| 219922_s_at | 4054 | LTBP3 | latent transforming growth factor beta binding protein 3 | 2.76 |
| 217842_at | 51631 | LUC7L2 | LUC7-like 2 (S. cerevisiae) | 2.11 |
| 203518_at | 1130 | LYST | lysosomal trafficking regulator | 2.23 |
| 213975_s_at | 4069 | LYZ | lysozyme (renal amyloidosis) | 2.01 |
| 207358_x_at, 208634_s_at, 214894_x_at | 23499 | MACF1 | microtubule-actin crosslinking factor 1 | 1.90, 2.28, 2.10 |
| 218559_s_at | 9935 | MAFB | v-maf musculoaponeurotic fibrosarcoma oncogene homolog B (avian) | 4.13 |
| 209737_at | 9863 | MAGI2 | membrane associated guanylate kinase, WW and PDZ domain containing 2 | 4.71 |
| 220945_x_at | 54682 | MANSC1 | MANSC domain containing 1 | 2.74 |
| 212233_at | 4131 | MAP1B | microtubule-associated protein 1B | 2.61 |
| 212566_at | 4134 | MAP4 | microtubule-associated protein 4 | 2.32 |
| 218181_s_at | 9448 | MAP4K4 | mitogen-activated protein kinase kinase kinase kinase 4 | 2.62 |
| 200644_at | 65108 | MARCKSL1 | MARCKS-like 1 | 2.15 |
| 201930_at | 4175 | MCM6 | minichromosome maintenance complex component 6 | 3.63 |
| 211675_s_at | 29969 | MDFIC | MyoD family inhibitor domain containing | 3.04 |
| 204058_at, 204059_s_at | 4199 | ME1 | malic enzyme 1, NADP(+)-dependent, cytosolic | 2.01, 1.97 |
| 209200_at | 4208 | MEF2C | myocyte enhancer factor 2C | 1.82 |
| 212830_at | 1955 | MEGF9 | multiple EGF-like-domains 9 | 3.12 |
| 207480_s_at | 4212 | MEIS2 | Meis homeobox 2 | 2.81 |
| 219858_s_at | 54842 | MFSD6 | major facilitator superfamily domain containing 6 | 2.1 |
| 212472_at, 212473_s_at | 9645 | MICAL2 | microtubule associated monoxygenase, calponin and LIM domain containing 2 | 3.87, 3.67 |
| 206247_at | 4277 | MICB | MHC class I polypeptide-related sequence B | 2.13 |
| 218071_s_at | 23609 | MKRN2 | makorin ring finger protein 2 | 1.89 |
| 203435_s_at | 4311 | MME | membrane metallo-endopeptidase | 1.79 |
| 219909_at | 79148 | MMP28 | matrix metallopeptidase 28 | 1.91 |
| 219321_at | 64398 | MPP5 | membrane protein, palmitoylated 5 (MAGUK p55 subfamily member 5) | 2.03 |
| 203801_at | 63931 | MRPS14 | mitochondrial ribosomal protein S14 | 2.56 |
| 204885_s_at | 10232 | MSLN | mesothelin | 2.29 |
| 201761_at | 10797 | MTHFD2 | methylenetetrahydrofolate dehydrogenase (NADP+ dependent) 2, methenyltetrahydrofolate cyclohydrolase | 3.65 |
| 214429_at | 9107 | MTMR6 | myotubularin related protein 6 | 2.09 |
| 212509_s_at | 439921 | MXRA7 | matrix-remodelling associated 7 | 2.29 |
| 213422_s_at | 54587 | MXRA8 | matrix-remodelling associated 8 | 2.55 |
| 211926_s_at | 4627 | MYH9 | myosin, heavy chain 9, non-muscle | 1.98 |
| 201058_s_at | 10398 | MYL9 | myosin, light chain 9, regulatory | 4.69 |
| 217623_at | 91807 | MYLK3 | myosin light chain kinase 3 | 2.89 |
| 212364_at | 4430 | MYO1B | myosin IB | 2.62 |
| 212338_at | 4642 | MYO1D | myosin ID | 3.66 |
| 203072_at | 4643 | MYO1E | myosin IE | 2.7 |
| 218966_at | 55930 | MYO5C | myosin VC | 1.81 |
| 201798_s_at, 211864_s_at | 26509 | MYOF | myoferlin | 2.61, 2.61 |
| 219509_at | 58529 | MYOZ1 | myozenin 1 | 2.27 |
| 213782_s_at | 51778 | MYOZ2 | myozenin 2 | 2.53 |
| 213375_s_at | 90634 | N4BP2L1 | NEDD4 binding protein 2-like 1 | 2.63 |
| 209272_at | 4664 | NAB1 | NGFI-A binding protein 1 (EGR1 binding protein 1) | 1.89 |
| 212993_at | 138151 | NACC2 | NACC family member 2, BEN and BTB (POZ) domain containing | 2.42 |
| 219368_at | 4674 | NAP1L2 | nucleosome assembly protein 1-like 2 | 1.98 |
| 204749_at | 4675 | NAP1L3 | nucleosome assembly protein 1-like 3 | 2.29 |
| 221207_s_at | 26960 | NBEA | neurobeachin | 1.85 |
| 203315_at | 8440 | NCK2 | NCK adaptor protein 2 | 1.95 |
| 209550_at | 4692 | NDN | necdin homolog (mouse) | 3.49 |
| 206453_s_at | 57447 | NDRG2 | NDRG family member 2 | 2.69 |
| 203961_at, 203962_s_at | 10529 | NEBL | nebulette | 3.99, 3.72 |
| 202149_at | 4739 | NEDD9 | neural precursor cell expressed, developmentally down-regulated 9 | 1.93 |
| 218678_at | 10763 | NES | nestin | 3.81 |
| 213438_at | 23114 | NFASC | neurofascin homolog (chicken) | 4.49 |
| 204702_s_at | 9603 | NFE2L3 | nuclear factor (erythroid-derived 2)-like 3 | 2.02 |
| 201591_s_at | 11188 | NISCH | nischarin | 2.3 |
| 218380_at | 22861 | NLRP1 | NLR family, pyrin domain containing 1 | 3.99 |
| 202443_x_at, 212377_s_at | 4853 | NOTCH2 | Notch homolog 2 (Drosophila) | 1.94,2.57 |
| 214722_at | 388677 | NOTCH2NL | Notch homolog 2 (Drosophila) N-terminal like | 2.1 |
| 214321_at | 4856 | NOV | nephroblastoma overexpressed gene | 3.27 |
| 202679_at | 4864 | NPC1 | Niemann-Pick disease, type C1 | 1.79 |
| 218086_at | 56654 | NPDC1 | neural proliferation, differentiation and control, 1 | 1.98 |
| 207673_at | 4868 | NPHS1 | nephrosis 1, congenital, Finnish type (nephrin) | 3.05 |
| 220424_at | 7827 | NPHS2 | nephrosis 2, idiopathic, steroid-resistant (podocin) | 3.57 |
| 32625_at | 4881 | NPR1 | natriuretic peptide receptor A/guanylate cyclase A (atrionatriuretic peptide receptor A) | 3.06 |
| 201468_s_at, 210519_s_at | 1728 | NQO1 | NAD(P)H dehydrogenase, quinone 1 | 2.66, 2.00 |
| 215104_at | 83714 | NRIP2 | nuclear receptor interacting protein 2 | 1.91 |
| 218625_at | 51299 | NRN1 | neuritin 1 | 2.34 |
| 221796_at | 4915 | NTRK2 | neurotrophic tyrosine kinase, receptor, type 2 | 3.45 |
| 213960_at | 4916 | NTRK3 | neurotrophic tyrosine kinase, receptor, type 3 | 1.86 |
| 200649_at | 4924 | NUCB1 | nucleobindin 1 | 1.92 |
| 219489_s_at | 64359 | NXN | nucleoredoxin | 3.55 |
| 219100_at | 79991 | OBFC1 | oligonucleotide/oligosaccharide-binding fold containing 1 | 1.81 |
| 212775_at | 23363 | OBSL1 | obscurin-like 1 | 1.77 |
| 219582_at | 79627 | OGFRL1 | opioid growth factor receptor-like 1 | 1.97 |
| 217525_at | 283298 | OLFML1 | olfactomedin-like 1 | 1.89 |
| 213075_at | 169611 | OLFML2A | olfactomedin-like 2A | 3.64 |
| 200897_s_at, 200907_s_at | 23022 | PALLD | palladin, cytoskeletal associated protein | 2.47, 1.84 |
| 213372_at | 152559 | PAQR3 | progestin and adipoQ receptor family member III | 2.34 |
| 221527_s_at | 56288 | PARD3 | par-3 partitioning defective 3 homolog (C. elegans) | 2.89 |
| 217890_s_at | 55742 | PARVA | parvin, alpha | 2.26 |
| 219295_s_at | 26577 | PCOLCE2 | procollagen C-endopeptidase enhancer 2 | 5.4 |
| 204735_at | 5141 | PDE4A | phosphodiesterase 4A, cAMP-specific (phosphodiesterase E2 dunce homolog, Drosophila) | 1.97 |
| 203708_at | 5142 | PDE4B | phosphodiesterase 4B, cAMP-specific (phosphodiesterase E4 dunce homolog, Drosophila) | 3.14 |
| 210305_at, 213388_at | 9659 | PDE4DIP | phosphodiesterase 4D interacting protein | 1.82, 3.26 |
| 219304_s_at | 80310 | PDGFD | platelet derived growth factor D | 2.63 |
| 202273_at | 5159 | PDGFRB | platelet-derived growth factor receptor, beta polypeptide | 1.82 |
| 208690_s_at | 9124 | PDLIM1 | PDZ and LIM domain 1 | 1.86 |
| 219165_at | 64236 | PDLIM2 | PDZ and LIM domain 2 (mystique) | 3.1 |
| 212412_at | 10611 | PDLIM5 | PDZ and LIM domain 5 | 2.21 |
| 221898_at | 10630 | PDPN | podoplanin | 4.55 |
| 200787_s_at, 200788_s_at | 8682 | PEA15 | phosphoprotein enriched in astrocytes 15 | 2.30, 3.11 |
| 208982_at | 5175 | PECAM1 | platelet/endothelial cell adhesion molecule | 2.99 |
| 209422_at | 51230 | PHF20 | PHD finger protein 20 | 1.92 |
| 205325_at | 9796 | PHYHIP | phytanoyl-CoA 2-hydroxylase interacting protein | 2.81 |
| 217863_at | 8554 | PIAS1 | protein inhibitor of activated STAT, 1 | 1.9 |
| 209785_s_at | 8605 | PLA2G4C | phospholipase A2, group IVC (cytosolic, calcium-independent) | 2.5 |
| 207415_at, 210194_at | 22925 | PLA2R1 | phospholipase A2 receptor 1, 180kDa | 4.06, 3.66 |
| 201860_s_at | 5327 | PLAT | plasminogen activator, tissue | 4 |
| 203895_at | 5332 | PLCB4 | phospholipase C, beta 4 | 2.04 |
| 205111_s_at, 205112_at | 51196 | PLCE1 | phospholipase C, epsilon 1 | 5.05, 4.72 |
| 201939_at | 10769 | PLK2 | polo-like kinase 2 (Drosophila) | 2.28 |
| 202620_s_at | 5352 | PLOD2 | procollagen-lysine, 2-oxoglutarate 5-dioxygenase 2 | 2.21 |
| 202075_s_at | 5360 | PLTP | phospholipid transfer protein | 3.6 |
| 221538_s_at | 5361 | PLXNA1 | plexin A1 | 2.05 |
| 38671_at | 23129 | PLXND1 | plexin D1 | 1.93 |
| 217875_s_at | 56937 | PMEPA1 | prostate transmembrane protein, androgen induced 1 | 1.85 |
| 209598_at | 10687 | PNMA2 | paraneoplastic antigen MA2 | 3.95 |
| 218824_at | 55228 | PNMAL1 | PNMA-like 1 | 2.07 |
| 201578_at | 5420 | PODXL | podocalyxin-like | 3.59 |
| 204441_s_at | 23649 | POLA2 | polymerase (DNA directed), alpha 2 (70kD subunit) | 2.53 |
| 201876_at | 5445 | PON2 | paraoxonase 2 | 2.24 |
| 210809_s_at | 10631 | POSTN | periostin, osteoblast specific factor | 2.85 |
| 209147_s_at, 210946_at | 8611 | PPAP2A | phosphatidic acid phosphatase type 2A | 2.38, 2.13 |
| 214978_s_at | 8497 | PPFIA4 | protein tyrosine phosphatase, receptor type, f polypeptide (PTPRF), interacting protein (liprin), alpha 4 | 3.37 |
| 203063_at | 9647 | PPM1F | protein phosphatase 1F (PP2C domain containing) | 1.89 |
| 213849_s_at | 5521 | PPP2R2B | protein phosphatase 2 (formerly 2A), regulatory subunit B, beta isoform | 2.74 |
| 201877_s_at | 5527 | PPP2R5C | protein phosphatase 2, regulatory subunit B', gamma isoform | 1.88 |
| 201594_s_at | 9989 | PPP4R1 | protein phosphatase 4, regulatory subunit 1 | 2.37 |
| 203680_at | 5577 | PRKAR2B | protein kinase, cAMP-dependent, regulatory, type II, beta | 4.72 |
| 218764_at | 5583 | PRKCH | protein kinase C, eta | 2.6 |
| 213518_at | 5584 | PRKCI | protein kinase C, iota | 2.42 |
| 202098_s_at, 221564_at | 3275 | PRMT2 | protein arginine methyltransferase 2 | 2.00, 2.18 |
| 218613_at | 23362 | PSD3 | pleckstrin and Sec7 domain containing 3 | 3.31 |
| 202659_at | 5699 | PSMB10 | proteasome (prosome, macropain) subunit, beta type, 10 | 1.9 |
| 209040_s_at | 5696 | PSMB8 | proteasome (prosome, macropain) subunit, beta type, 8 (large multifunctional peptidase 7) | 2.56 |
| 204279_at | 5698 | PSMB9 | proteasome (prosome, macropain) subunit, beta type, 9 (large multifunctional peptidase 2) | 2.67 |
| 211663_x_at, 211748_x_at, 212187_x_at | 5730 | PTGDS | prostaglandin D2 synthase 21kDa (brain) | 4.69, 3.96, 4.20 |
| 204897_at | 5734 | PTGER4 | prostaglandin E receptor 4 (subtype EP4) | 4.68 |
| 204201_s_at | 5783 | PTPN13 | protein tyrosine phosphatase, non-receptor type 13 (APO-1/CD95 (Fas)-associated phosphatase) | 2.12 |
| 213795_s_at | 5786 | PTPRA | protein tyrosine phosphatase, receptor type, A | 1.96 |
| 205846_at, 217177_s_at | 5787 | PTPRB | protein tyrosine phosphatase, receptor type, B | 1.88, 3.02 |
| 214043_at | 5789 | PTPRD | protein tyrosine phosphatase, receptor type, D | 1.84 |
| 203029_s_at | 5799 | PTPRN2 | protein tyrosine phosphatase, receptor type, N polypeptide 2 | 2.43 |
| 208121_s_at | 5800 | PTPRO | protein tyrosine phosphatase, receptor type, O | 4.97 |
| 208789_at | 284119 | PTRF | polymerase I and transcript release factor | 1.97 |
| 212662_at | 5817 | PVR | poliovirus receptor | 2.07 |
| 203149_at | 5819 | PVRL2 | poliovirus receptor-related 2 (herpesvirus entry mediator B) | 1.97 |
| 212012_at | 7837 | PXDN | peroxidasin homolog (Drosophila) | 1.87 |
| 212636_at | 9444 | QKI | quaking homolog, KH domain RNA binding (mouse) | 3.87 |
| 217764_s_at | 11031 | RAB31 | RAB31, member RAS oncogene family | 2.94 |
| 203020_at, 213982_s_at | 9910 | RABGAP1L | RAB GTPase activating protein 1-like | 2.23, 2.67 |
| 202052_s_at | 26064 | RAI14 | retinoic acid induced 14 | 2.15 |
| 205779_at | 10266 | RAMP2 | receptor (G protein-coupled) activity modifying protein 2 | 1.81 |
| 205326_at | 10268 | RAMP3 | receptor (G protein-coupled) activity modifying protein 3 | 2.65 |
| 210051_at | 10411 | RAPGEF3 | Rap guanine nucleotide exchange factor (GEF) 3 | 2.32 |
| 204681_s_at | 9771 | RAPGEF5 | Rap guanine nucleotide exchange factor (GEF) 5 | 1.93 |
| 209496_at | 5919 | RARRES2 | retinoic acid receptor responder (tazarotene induced) 2 | 2.15 |
| 205801_s_at | 25780 | RASGRP3 | RAS guanyl releasing protein 3 (calcium and DAG-regulated) | 2.3 |
| 219142_at | 65997 | RASL11B | RAS-like, family 11, member B | 3.23 |
| 203132_at | 5925 | RB1 | retinoblastoma 1 | 1.78 |
| 216215_s_at | 23543 | RBM9 | RNA binding motif protein 9 | 2.17 |
| 201486_at | 5955 | RCN2 | reticulocalbin 2, EF-hand calcium binding domain | 1.8 |
| 205407_at | 8434 | RECK | reversion-inducing-cysteine-rich protein with kazal motifs | 3.96 |
| 208021_s_at | 5981 | RFC1 | replication factor C (activator 1) 1, 145kDa | 2.06 |
| 204316_at, 204319_s_at | 6001 | RGS10 | regulator of G-protein signaling 10 | 1.88, 2.57 |
| 202388_at | 5997 | RGS2 | regulator of G-protein signaling 2, 24kDa | 1.8 |
| 212122_at | 23433 | RHOQ | ras homolog gene family, member Q | 2.34 |
| 201785_at | 6035 | RNASE1 | ribonuclease, RNase A family, 1 (pancreatic) | 1.91 |
| 219988_s_at | 55182 | RNF220 | ring finger protein 220 | 3.01 |
| 218528_s_at | 152006 | RNF38 | ring finger protein 38 | 1.83 |
| 213194_at | 6091 | ROBO1 | roundabout, axon guidance receptor, homolog 1 (Drosophila) | 3.7 |
| 213044_at | 6093 | ROCK1 | Rho-associated, coiled-coil containing protein kinase 1 | 2.05 |
| 221614_s_at | 9501 | RPH3AL | rabphilin 3A-like (without C2 domains) | 1.84 |
| 212647_at | 6237 | RRAS | related RAS viral (r-ras) oncogene homolog | 3.72 |
| 222204_s_at | 54700 | RRN3 | RRN3 RNA polymerase I transcription factor homolog (S. cerevisiae) | 2.05 |
| 212846_at | 23076 | RRP1B | ribosomal RNA processing 1 homolog B (S. cerevisiae) | 1.97 |
| 34408_at | 6253 | RTN2 | reticulon 2 | 2.12 |
| 206306_at | 6263 | RYR3 | ryanodine receptor 3 | 2.37 |
| 203186_s_at | 6275 | S100A4 | S100 calcium binding protein A4 | 2.48 |
| 202917_s_at | 6279 | S100A8 | S100 calcium binding protein A8 | 1.8 |
| 204642_at | 1901 | S1PR1 | sphingosine-1-phosphate receptor 1 | 2.17 |
| 213262_at | 26278 | SACS | spastic ataxia of Charlevoix-Saguenay (sacsin) | 1.81 |
| 208740_at | 10284 | SAP18 | Sin3A-associated protein, 18kDa | 1.86 |
| 200069_at | 9733 | SART3 | squamous cell carcinoma antigen recognized by T cells 3 | 2.1 |
| 204030_s_at | 29970 | SCHIP1 | schwannomin interacting protein 1 | 2.13 |
| 201462_at | 9805 | SCRN1 | secernin 1 | 1.81 |
| 212157_at | 6383 | SDC2 | syndecan 2 | 1.79 |
| 218649_x_at | 9147 | SDCCAG1 | serologically defined colon cancer antigen 1 | 1.82 |
| 202084_s_at | 6397 | SEC14L1 | SEC14-like 1 (S. cerevisiae) | 1.86 |
| 202375_at | 9871 | SEC24D | SEC24 family, member D (S. cerevisiae) | 1.98 |
| 203071_at | 7869 | SEMA3B | sema domain, immunoglobulin domain (Ig), short basic domain, secreted, (semaphorin) 3B | 2.17 |
| 203789_s_at | 10512 | SEMA3C | sema domain, immunoglobulin domain (Ig), short basic domain, secreted, (semaphorin) 3C | 1.92 |
| 219689_at | 56920 | SEMA3G | sema domain, immunoglobulin domain (Ig), short basic domain, secreted, (semaphorin) 3G | 4.69 |
| 213169_at | 9037 | SEMA5A | sema domain, seven thrombospondin repeats (type 1 and type 1-like), transmembrane domain (TM) and short cytoplasmic domain, (semaphorin) 5A | 3.24 |
| 201307_at, 214293_at | 55752 | 11-Sep | septin 11 | 2.33, 3.39 |
| 213151_s_at | 989 | 7-Sep | septin 7 | 1.78 |
| 221472_at | 10955 | SERINC3 | serine incorporator 3 | 2.72 |
| 209723_at | 5272 | SERPINB9 | serpin peptidase inhibitor, clade B (ovalbumin), member 9 | 2.1 |
| 200986_at | 710 | SERPING1 | serpin peptidase inhibitor, clade G (C1 inhibitor), member 1 | 1.94 |
| 202657_s_at | 9792 | SERTAD2 | SERTA domain containing 2 | 1.92 |
| 221768_at | 6421 | SFPQ | Myoblast cell surface antigen 24.1D5 | 2.47 |
| 212179_at | 25957 | SFRS18 | splicing factor, arginine/serine-rich 18 | 1.89 |
| 35626_at | 6448 | SGSH | N-sulfoglucosamine sulfohydrolase | 2.57 |
| 209090_s_at | 51100 | SH3GLB1 | SH3-domain GRB2-like endophilin B1 | 2.66 |
| 219083_at | 55164 | SHQ1 | SHQ1 homolog (S. cerevisiae) | 2.42 |
| 204967_at | 357 | SHROOM2 | shroom family member 2 | 1.81 |
| 208078_s_at | 150094 | SIK1 | salt-inducible kinase 1 | 2.54 |
| 202897_at | 140885 | SIRPA | signal-regulatory protein alpha | 2.46 |
| 205856_at | 6563 | SLC14A1 | solute carrier family 14 (urea transporter), member 1 (Kidd blood group) | 2.39 |
| 206081_at | 9187 | SLC24A1 | solute carrier family 24 (sodium/potassium/calcium exchanger), member 1 | 1.84 |
| 219090_at, 57588_at | 57419 | SLC24A3 | solute carrier family 24 (sodium/potassium/calcium exchanger), member 3 | 2.88, 2.77 |
| 221024_s_at | 81031 | SLC2A10 | solute carrier family 2 (facilitated glucose transporter), member 10 | 2.62 |
| 202499_s_at | 6515 | SLC2A3 | solute carrier family 2 (facilitated glucose transporter), member 3 | 1.85 |
| 202088_at | 25800 | SLC39A6 | solute carrier family 39 (zinc transporter), member 6 | 2.05 |
| 218416_s_at, 218417_s_at | 55652 | SLC48A1 | solute carrier family 48 (heme transporter), member 1 | 2.76, 1.91 |
| 206875_s_at | 9748 | SLK | STE20-like kinase (yeast) | 2.72 |
| 217828_at | 79811 | SLTM | SAFB-like, transcription modulator | 1.8 |
| 207069_s_at | 4091 | SMAD6 | SMAD family member 6 | 2.52 |
| 213720_s_at | 6597 | SMARCA4 | SWI/SNF related, matrix associated, actin dependent regulator of chromatin, subfamily a, member 4 | 1.83 |
| 204099_at | 6604 | SMARCD3 | SWI/SNF related, matrix associated, actin dependent regulator of chromatin, subfamily d, member 3 | 1.98 |
| 212569_at | 23347 | SMCHD1 | structural maintenance of chromosomes flexible hinge domain containing 1 | 1.82 |
| 202508_s_at | 6616 | SNAP25 | synaptosomal-associated protein, 25kDa | 1.97 |
| 204466_s_at, 204467_s_at | 6622 | SNCA | synuclein, alpha (non A4 component of amyloid precursor) | 3.57, 2.96 |
| 213364_s_at | 6642 | SNX1 | sorting nexin 1 | 2.35 |
| 217789_at | 58533 | SNX6 | sorting nexin 6 | 1.85 |
| 212807_s_at | 6272 | SORT1 | sortilin 1 | 2.01 |
| 210219_at | 6672 | SP100 | SP100 nuclear antigen | 1.8 |
| 200665_s_at, 212667_at | 6678 | SPARC | secreted protein, acidic, cysteine-rich (osteonectin) | 3.37, 3.07 |
| 202363_at | 6695 | SPOCK1 | sparc/osteonectin, cwcv and kazal-like domains proteoglycan (testican) 1 | 3.97 |
| 202523_s_at, 202524_s_at | 9806 | SPOCK2 | sparc/osteonectin, cwcv and kazal-like domains proteoglycan (testican) 2 | 2.06, 4.13 |
| 208611_s_at, 215235_at | 6709 | SPTAN1 | spectrin, alpha, non-erythrocytic 1 (alpha-fodrin) | 2.94, 3.60 |
| 217995_at | 58472 | SQRDL | sulfide quinone reductase-like (yeast) | 1.9 |
| 213329_at | 23380 | SRGAP2 | SLIT-ROBO Rho GTPase activating protein 2 | 2.86 |
| 201859_at | 5552 | SRGN | serglycin | 1.78 |
| 203787_at | 23635 | SSBP2 | single-stranded DNA binding protein 2 | 2.8 |
| 213921_at | 6750 | SST | somatostatin | 1.82 |
| 210942_s_at, 213355_at | 10402 | ST3GAL6 | ST3 beta-galactoside alpha-2,3-sialyltransferase 6 | 2.70, 3.55 |
| 200887_s_at, 209969_s_at, AFFX-HUMISGF3A/M97935_3_at | 6772 | STAT1 | signal transducer and activator of transcription 1, 91kDa | 2.14, 1.90, 2.36 |
| 213413_at | 11037 | STON1 | stonin 1 | 3.84 |
| 203769_s_at | 412 | STS | steroid sulfatase (microsomal), isozyme S | 1.81 |
| 202260_s_at | 6812 | STXBP1 | syntaxin binding protein 1 | 2.02 |
| 212344_at, 212353_at, 212354_at | 23213 | SULF1 | sulfatase 1 | 3.57, 5.05, 4.57 |
| 209307_at | 23075 | SWAP70 | SWAP-70 protein | 2.11 |
| 209447_at | 23345 | SYNE1 | spectrin repeat containing, nuclear envelope 1 | 2.08 |
| 212828_at | 8871 | SYNJ2 | synaptojanin 2 | 3.55 |
| 202796_at | 11346 | SYNPO | synaptopodin | 4.78 |
| 202289_s_at | 10579 | TACC2 | transforming, acidic coiled-coil containing protein 2 | 2.51 |
| 202840_at | 8148 | TAF15 | TAF15 RNA polymerase II, TATA box binding protein (TBP)-associated factor, 68kDa | 1.94 |
| 200916_at | 8407 | TAGLN2 | transgelin 2 | 1.85 |
| 219443_at | 55617 | TASP1 | taspase, threonine aspartase, 1 | 2.28 |
| 212796_s_at | 23102 | TBC1D2B | TBC1 domain family, member 2B | 2.49 |
| 216194_s_at | 1155 | TBCB | tubulin folding cofactor B | 1.92 |
| 219682_s_at | 6926 | TBX3 | T-box 3 | 2.57 |
| 207554_x_at, 336_at | 6915 | TBXA2R | thromboxane A2 receptor | 2.11, 2.24 |
| 211276_at | 140597 | TCEAL2 | transcription elongation factor A (SII)-like 2 | 3.91 |
| 202396_at | 10915 | TCERG1 | transcription elongation regulator 1 | 1.86 |
| 204931_at | 6943 | TCF21 | transcription factor 21 | 5.89 |
| 213891_s_at | 6925 | TCF4 | transcription factor 4 | 1.96 |
| 221016_s_at | 83439 | TCF7L1 | transcription factor 7-like 1 (T-cell specific, HMG-box) | 2.41 |
| 203743_s_at | 6996 | TDG | thymine-DNA glycosylase | 1.84 |
| 206702_at | 7010 | TEK | TEK tyrosine kinase, endothelial | 2.45 |
| 212494_at | 23371 | TENC1 | tensin like C1 domain containing phosphatase (tensin 2) | 2.98 |
| 209651_at | 7041 | TGFB1I1 | transforming growth factor beta 1 induced transcript 1 | 2.7 |
| 208944_at | 7048 | TGFBR2 | transforming growth factor, beta receptor II (70/80kDa) | 2.98 |
| 213894_at, 214920_at | 221981 | THSD7A | thrombospondin, type I, domain containing 7A | 2.47, 2.53 |
| 201448_at | 7072 | TIA1 | TIA1 cytotoxic granule-associated RNA binding protein | 1.81 |
| 213135_at | 7074 | TIAM1 | T-cell lymphoma invasion and metastasis 1 | 2.2 |
| 201666_at | 7076 | TIMP1 | TIMP metallopeptidase inhibitor 1 | 3.1 |
| 201150_s_at | 7078 | TIMP3 | TIMP metallopeptidase inhibitor 3 | 3.53 |
| 212665_at | 25976 | TIPARP | TCDD-inducible poly(ADP-ribose) polymerase | 1.8 |
| 202011_at | 7082 | TJP1 | tight junction protein 1 (zona occludens 1) | 2.51 |
| 203221_at | 7088 | TLE1 | transducin-like enhancer of split 1 (E(sp1) homolog, Drosophila) | 1.99 |
| 204872_at | 7091 | TLE4 | transducin-like enhancer of split 4 (E(sp1) homolog, Drosophila) | 2.51 |
| 213882_at | 83941 | TM2D1 | TM2 domain containing 1 | 1.97 |
| 209386_at, 209387_s_at, 215034_s_at | 4071 | TM4SF1 | transmembrane 4 L six family member 1 | 2.16, 2.09, 2.00 |
| 220240_s_at | 55002 | TMCO3 | transmembrane and coiled-coil domains 3 | 1.86 |
| 208837_at | 23423 | TMED3 | transmembrane emp24 protein transport domain containing 3 | 2.17 |
| 219315_s_at | 79652 | TMEM204 | transmembrane protein 204 | 2.52 |
| 219410_at | 55076 | TMEM45A | transmembrane protein 45A | 3.09 |
| 209656_s_at | 83604 | TMEM47 | transmembrane protein 47 | 2.02 |
| 203662_s_at | 7111 | TMOD1 | tropomodulin 1 | 2.53 |
| 202561_at | 8658 | TNKS | tankyrase, TRF1-interacting ankyrin-related ADP-ribose polymerase | 2.32 |
| 209904_at | 7134 | TNNC1 | troponin C type 1 (slow) | 2.04 |
| 205177_at | 7135 | TNNI1 | troponin I type 1 (skeletal, slow) | 3.7 |
| 215389_s_at | 7139 | TNNT2 | troponin T type 2 (cardiac) | 4.82 |
| 212635_at | 3842 | TNPO1 | transportin 1 | 1.93 |
| 217853_at | 64759 | TNS3 | tensin 3 | 2.34 |
| 202633_at | 11073 | TOPBP1 | topoisomerase (DNA) II binding protein 1 | 1.85 |
| 203476_at | 7162 | TPBG | trophoblast glycoprotein | 1.87 |
| 201691_s_at | 7163 | TPD52 | tumor protein D52 | 2.05 |
| 204083_s_at | 7169 | TPM2 | tropomyosin 2 (beta) | 4.85 |
| 218876_at | 51673 | TPPP3 | tubulin polymerization-promoting protein family member 3 | 5.35 |
| 221571_at | 7187 | TRAF3 | TNF receptor-associated factor 3 | 1.81 |
| 204352_at | 7188 | TRAF5 | TNF receptor-associated factor 5 | 2.84 |
| 202369_s_at | 9697 | TRAM2 | translocation associated membrane protein 2 | 2.88 |
| 202478_at | 28951 | TRIB2 | tribbles homolog 2 (Drosophila) | 2.53 |
| 213293_s_at | 10346 | TRIM22 | tripartite motif-containing 22 | 1.83 |
| 205803_s_at | 7220 | TRPC1 | transient receptor potential cation channel, subfamily C, member 1 | 1.78 |
| 208763_s_at | 1831 | TSC22D3 | TSC22 domain family, member 3 | 2.55 |
| 214606_at | 10100 | TSPAN2 | tetraspanin 2 | 1.94 |
| 209263_x_at | 7106 | TSPAN4 | tetraspanin 4 | 1.82 |
| 202242_at | 7102 | TSPAN7 | tetraspanin 7 | 1.78 |
| 218012_at | 64061 | TSPYL2 | TSPY-like 2 | 1.85 |
| 212928_at | 23270 | TSPYL4 | TSPY-like 4 | 1.9 |
| 213122_at | 85453 | TSPYL5 | TSPY-like 5 | 1.99 |
| 209118_s_at | 7846 | TUBA1A | tubulin, alpha 1a | 2.22 |
| 213266_at | 27229 | TUBGCP4 | Tubulin, gamma complex associated protein 4, mRNA (cDNA clone MGC:2720 IMAGE:2821891) | 2.65 |
| 212725_s_at | 55000 | TUG1 | taurine upregulated 1 (non-protein coding) | 1.96 |
| 209227_at, 209228_x_at, 213423_x_at | 7991 | TUSC3 | tumor suppressor candidate 3 | 2.13, 2.27, 2.13 |
| 211431_s_at, 211432_s_at | 7301 | TYRO3 | TYRO3 protein tyrosine kinase | 2.64, 3.30 |
| 219192_at | 55833 | UBAP2 | ubiquitin associated protein 2 | 2.08 |
| 208760_at | 7329 | UBE2I | Ubiquitin-conjugating enzyme UBC9 | 1.9 |
| 201649_at | 9246 | UBE2L6 | ubiquitin-conjugating enzyme E2L 6 | 2.39 |
| 218801_at | 55757 | UGCGL2 | UDP-glucose ceramide glucosyltransferase-like 2 | 2.8 |
| 203519_s_at | 26019 | UPF2 | UPF2 regulator of nonsense transcripts homolog (yeast) | 1.88 |
| 218757_s_at | 65109 | UPF3B | UPF3 regulator of nonsense transcripts homolog B (yeast) | 2.35 |
| 202413_s_at | 7398 | USP1 | ubiquitin specific peptidase 1 | 1.98 |
| 218386_x_at | 10600 | USP16 | ubiquitin specific peptidase 16 | 1.87 |
| 203869_at, 203870_at | 64854 | USP46 | ubiquitin specific peptidase 46 | 3.26, 3.81 |
| 213326_at | 6843 | VAMP1 | vesicle-associated membrane protein 1 (synaptobrevin 1) | 1.87 |
| 201556_s_at | 6844 | VAMP2 | vesicle-associated membrane protein 2 (synaptobrevin 2) | 2.47 |
| 213480_at | 8674 | VAMP4 | vesicle-associated membrane protein 4 | 2.2 |
| 204929_s_at | 10791 | VAMP5 | vesicle-associated membrane protein 5 (myobrevin) | 2.26 |
| 203940_s_at | 22846 | VASH1 | vasohibin 1 | 2.07 |
| 210512_s_at, 212171_x_at | 7422 | VEGFA | vascular endothelial growth factor A | 2.72, 1.84 |
| 212399_s_at | 9686 | VGLL4 | vestigial like 4 (Drosophila) | 2.07 |
| 209950_s_at | 50853 | VILL | villin-like | 2.05 |
| 201426_s_at | 7431 | VIM | vimentin | 2.53 |
| 217949_s_at | 79001 | VKORC1 | vitamin K epoxide reductase complex, subunit 1 | 1.93 |
| 212326_at | 55187 | VPS13D | vacuolar protein sorting 13 homolog D (S. cerevisiae) | 1.78 |
| 200629_at | 7453 | WARS | tryptophanyl-tRNA synthetase | 1.9 |
| 202250_s_at | 50717 | WDR42A | WD repeat domain 42A | 2.43 |
| 202908_at | 7466 | WFS1 | Wolfram syndrome 1 (wolframin) | 2.54 |
| 210861_s_at | 8838 | WISP3 | WNT1 inducible signaling pathway protein 3 | 2.17 |
| 206067_s_at, 216953_s_at | 7490 | WT1 | Wilms tumor 1 | 4.58, 2.39 |
| 219520_s_at | 55841 | WWC3 | WWC family member 3 | 1.88 |
| 212637_s_at | 11059 | WWP1 | WW domain containing E3 ubiquitin protein ligase 1 | 2.08 |
| 213725_x_at | 64131 | XYLT1 | xylosyltransferase I | 2.4 |
| 218833_at | 51776 | ZAK | sterile alpha motif and leucine zipper containing kinase AZK | 1.98 |
| 218348_s_at | 29066 | ZC3H7A | zinc finger CCCH-type containing 7A | 1.95 |
| 212704_at | 23318 | ZCCHC11 | zinc finger, CCHC domain containing 11 | 1.78 |
| 218249_at | 64429 | ZDHHC6 | zinc finger, DHHC-type containing 6 | 3.06 |
| 212764_at | 6935 | ZEB1 | zinc finger E-box binding homeobox 1 | 3.42 |
| 203603_s_at | 9839 | ZEB2 | zinc finger E-box binding homeobox 2 | 2.28 |
| 222237_s_at | 7771 | ZFP112 | zinc finger protein 112 homolog (mouse) | 2.3 |
| 219778_at | 23414 | ZFPM2 | zinc finger protein, multitype 2 | 3.43 |
| 202049_s_at | 9202 | ZMYM4 | zinc finger, MYM-type 4 | 1.96 |
| 207417_s_at | 7730 | ZNF177 | zinc finger protein 177 | 2.06 |
| 203585_at | 7739 | ZNF185 | zinc finger protein 185 (LIM domain) | 2.21 |
| 214761_at | 23090 | ZNF423 | zinc finger protein 423 | 2.29 |
| 215767_at | 91752 | ZNF804A | zinc finger protein 804A | 4.25 |
