# Supplementary material for: Systematic Analysis of a Novel Human Renal Glomerulus-Enriched Gene Expression Dataset
Source: PLoS One. 2010 Jul 12;5(7):e11545. doi: 10.1371/journal.pone.0011545 (PMC2902524; doi:10.1371/journal.pone.0011545)
Supplement: Table S2 — List of known podocyte-, mesangial- and endothelial-specific markers as well as validated glomerular gene and protein expression data. (0.35 MB DOC) [file pone.0011545.s003.doc]

Table S2

| **Entrez Gene** | **Gene Symbol** | **Gene Name** | **Cell type** | **Reference** |
| --- | --- | --- | --- | --- |
| 59 | ACTA2 | actin, alpha 2, smooth muscle, aorta | mesangial | [1] |
| 81 | ACTN4 | actinin, alpha 4 | podocyte | [1] |
| 107 | ADCY1 | adenylate cyclase 1 (brain) | podocyte | [1] |
| 133 | ADM | adrenomedullin | glomerular | [1] |
| 177 | AGER | advanced glycosylation end product-specific receptor | podocyte | [1] |
| 375790 | AGRN | agrin | glomerular | [1] |
| 57085 | AGTRAP | angiotensin II receptor-associated protein | glomerular | [1] |
| 203 | AK1 | adenylate kinase 1 | podocyte | [1] |
| 284 | ANGPT1 | angiopoietin 1 | glomerular | [1] |
| 285 | ANGPT2 | angiopoietin 2 | mesangial | [1] |
| 23452 | ANGPTL2 | angiopoietin-like 2 | podocyte | [1,2] |
| 405 | ARNT | aryl hydrocarbon receptor nuclear translocator | glomerular | [1] |
| 421 | ARVCF | armadillo repeat gene deletes in velocardiofacial syndrome | podocyte | [1,2] |
| 79827 | ASAM | adipocyte-specific adhesion molecule | glomerular | [1] |
| 4059 | BCAM | basal cell adhesion molecule (Lutheran blood group) | glomerular | [1] |
| 9564 | BCAR1 | breast cancer anti-estrogen resistance 1 | glomerular | [1] |
| 655 | BMP7 | bone morphogenetic protein 7 | glomerular | [1] |
| 114897 | C1QTNF1 | C1q and tumor necrosis factor related protein 1 | podocyte | [1] |
| 800 | CALD1 | caldesmon 1 | glomerular | [1] |
| 10983 | CCNI | cyclin I | podocyte | [2] |
| 23607 | CD2AP | CD2-associated protein | podocyte | [1] |
| 947 | CD34 | CD34 molecule | mesangial | [1] |
| 1604 | CD55 | CD55 molecule, decay accelerating factor for complement (Cromer blood group) | podocyte | [2] |
| 941 | CD80 | CD80 molecule | podocyte | [3] |
| 1012 | CDH13 | cadherin 13, H-cadherin (heart) | podocyte | [4] |
| 1001 | CDH3 | cadherin 3, type 1, P-cadherin (placental) | podocyte | [1] |
| 1003 | CDH5 | cadherin 5, type 2 (vascular endothelium) | glomerular | [1] |
| 1027 | CDKN1B | cyclin-dependent kinase inhibitor 1B (p27, Kip1) | glomerular | [1] |
| 1028 | CDKN1C | cyclin-dependent kinase inhibitor 1C (p57, Kip2) | podocyte | [1] |
| 10370 | CITED2 | Cbp/p300-interacting transactivator, with Glu/Asp-rich carboxy-terminal domain, 2 | podocyte | [1] |
| 7122 | CLDN5 | claudin 5 | endothelial | [1] |
| 9022 | CLIC3 | chloride intracellular channel 3 | podocyte | [1,2] |
| 80781 | COL18A1 | collagen, type XVIII, alpha 1 | glomerular | [1] |
| 1282 | COL4A1 | collagen, type IV, alpha 1 | glomerular | [1] |
| 1284 | COL4A2 | collagen, type IV, alpha 2 | glomerular | [1] |
| 1285 | COL4A3 | collagen, type IV, alpha 3 (Goodpasture antigen) | glomerular | [1] |
| 1287 | COL4A5 | collagen, type IV, alpha 5 | glomerular | [1] |
| 1378 | CR1 | complement component (3b/4b) receptor 1 (Knops blood group) | podocyte | [5] |
| 51232 | CRIM1 | cysteine rich transmembrane BMP regulator 1 (chordin-like) | glomerular | [1] |
| 1490 | CTGF | connective tissue growth factor | glomerular | [1] |
| 1499 | CTNNB1 | catenin (cadherin-associated protein), beta 1, 88kDa | podocyte | [1] |
| 1500 | CTNND1 | catenin (cadherin-associated protein), delta 1 | glomerular | [1] |
| 1515 | CTSL2 | cathepsin L2 | glomerular | [1] |
| 2017 | CTTN | cortactin | podocyte | [1] |
| 8065 | CUL5 | cullin 5 | glomerular | [1] |
| 1523 | CUX1 | cut-like homeobox 1 | glomerular | [1] |
| 6376 | CX3CL1 | chemokine (C-X3-C motif) ligand 1 | glomerular | [1] |
| 6387 | CXCL12 | chemokine (C-X-C motif) ligand 12 (stromal cell-derived factor 1) | glomerular | [1] |
| 2833 | CXCR3 | chemokine (C-X-C motif) receptor 3 | glomerular | [1] |
| 1580 | CYP4B1 | cytochrome P450, family 4, subfamily B, polypeptide 1 | mesangial | [1] |
| 1605 | DAG1 | dystroglycan 1 (dystrophin-associated glycoprotein 1) | podocyte | [1] |
| 28988 | DBNL | drebrin-like | glomerular | [1] |
| 23109 | DDN | dendrin | podocyte | [2] |
| 1674 | DES | desmin | glomerular | [1] |
| 28514 | DLL1 | delta-like 1 (Drosophila) | glomerular | [1] |
| 1942 | EFNA1 | ephrin-A1 | glomerular | [1] |
| 1947 | EFNB1 | ephrin-B1 | podocyte | [1] |
| 1948 | EFNB2 | ephrin-B2 | glomerular | [1] |
| 30845 | EHD3 | EH-domain containing 3 | endothelial | [6] |
| 10480 | EIF3M | eukaryotic translation initiation factor 3, subunit M | podocyte | [1] |
| 51705 | EMCN | endomucin | endothelial | [1] |
| 2022 | ENG | endoglin | glomerular | [1] |
| 2028 | ENPEP | glutamyl aminopeptidase (aminopeptidase A) | glomerular | [1] |
| 2034 | EPAS1 | endothelial PAS domain protein 1 | glomerular | [1] |
| 90332 | EXOC3L2 | exocyst complex component 3-like 2 | mesangial | [1] |
| 7430 | EZR | ezrin | podocyte | [1] |
| 2149 | F2R | coagulation factor II (thrombin) receptor | mesangial | [1] |
| 2195 | FAT1 | FAT tumor suppressor homolog 1 (Drosophila) | podocyte | [7] |
| 2247 | FGF2 | fibroblast growth factor 2 (basic) | glomerular | [1] |
| 2260 | FGFR1 | fibroblast growth factor receptor 1 | glomerular | [1] |
| 2321 | FLT1 | fms-related tyrosine kinase 1 (vascular endothelial growth factor/vascular permeability factor receptor) | endothelial | [1] |
| 2324 | FLT4 | fms-related tyrosine kinase 4 | glomerular | [1] |
| 2335 | FN1 | fibronectin 1 | glomerular | [1] |
| 2303 | FOXC2 | forkhead box C2 (MFH-1, mesenchyme forkhead 1) | podocyte | [1] |
| 2534 | FYN | FYN oncogene related to SRC, FGR, YES | podocyte | [2] |
| 55568 | GALNT10 | UDP-N-acetyl-alpha-D-galactosamine:polypeptide N-acetylgalactosaminyltransferase 10 (GalNAc-T10) | podocyte | [1] |
| 2625 | GATA3 | GATA binding protein 3 | glomerular | [1] |
| 23062 | GGA2 | golgi associated, gamma adaptin ear containing, ARF binding protein 2 | glomerular | [1] |
| 2697 | GJA1 | gap junction protein, alpha 1, 43kDa | glomerular | [1] |
| 2702 | GJA5 | gap junction protein, alpha 5, 40kDa | glomerular | [1] |
| 113263 | GLCCI1 | glucocorticoid induced transcript 1 | podocyte | [1,2] |
| 221395 | GPR116 | G protein-coupled receptor 116 | podocyte | [1] |
| 3068 | HDGF | hepatoma-derived growth factor (high-mobility group protein 1-like) | glomerular | [1] |
| 3091 | HIF1A | hypoxia inducible factor 1, alpha subunit (basic helix-loop-helix transcription factor) | glomerular | [1] |
| 3213 | HOXB3 | homeobox B3 | glomerular | [1] |
| 3309 | HSPA5 | heat shock 70kDa protein 5 (glucose-regulated protein, 78kDa) | glomerular | [1] |
| 3315 | HSPB1 | heat shock 27kDa protein 1 | glomerular | [1] |
| 3339 | HSPG2 | heparan sulfate proteoglycan 2 | glomerular | [1] |
| 3384 | ICAM2 | intercellular adhesion molecule 2 | endothelial | [1] |
| 3481 | IGF2 | insulin-like growth factor 2 (somatomedin A) | glomerular | [1] |
| 3486 | IGFBP3 | insulin-like growth factor binding protein 3 | glomerular | [1] |
| 3488 | IGFBP5 | insulin-like growth factor binding protein 5 | mesangial | [1] |
| 3611 | ILK | integrin-linked kinase | glomerular | [1] |
| 3673 | ITGA2 | integrin, alpha 2 (CD49B, alpha 2 subunit of VLA-2 receptor) | glomerular | [1] |
| 3675 | ITGA3 | integrin, alpha 3 (antigen CD49C, alpha 3 subunit of VLA-3 receptor) | glomerular | [1] |
| 3678 | ITGA5 | integrin, alpha 5 (fibronectin receptor, alpha polypeptide) | endothelial | [1] |
| 3655 | ITGA6 | integrin, alpha 6 | glomerular | [1] |
| 8516 | ITGA8 | integrin, alpha 8 | mesangial | [1] |
| 3685 | ITGAV | integrin, alpha V (vitronectin receptor, alpha polypeptide, antigen CD51) | glomerular | [1] |
| 3688 | ITGB1 | integrin, beta 1 (fibronectin receptor, beta polypeptide, antigen CD29 includes MDF2, MSK12) | glomerular | [1] |
| 3693 | ITGB5 | integrin, beta 5 | podocyte | [1] |
| 182 | JAG1 | jagged 1 (Alagille syndrome) | glomerular | [1] |
| 3718 | JAK3 | Janus kinase 3 | glomerular | [1] |
| 3728 | JUP | junction plakoglobin | glomerular | [1] |
| 3791 | KDR | kinase insert domain receptor (a type III receptor tyrosine kinase) | glomerular | [1] |
| 1316 | KLF6 | Kruppel-like factor 6 | mesangial | [1] |
| 3911 | LAMA5 | laminin, alpha 5 | glomerular | [1] |
| 3912 | LAMB1 | laminin, beta 1 | glomerular | [1] |
| 3913 | LAMB2 | laminin, beta 2 (laminin S) | glomerular | [1] |
| 3915 | LAMC1 | laminin, gamma 1 (formerly LAMB2) | glomerular | [1] |
| 9079 | LDB2 | LIM domain binding 2 | mesangial | [1] |
| 3965 | LGALS9 | lectin, galactoside-binding, soluble, 9 | glomerular | [1] |
| 4008 | LMO7 | LIM domain 7 | mesangial | [1] |
| 4010 | LMX1B | LIM homeobox transcription factor 1, beta | podocyte | [1] |
| 57554 | LRRC7 | leucine rich repeat containing 7 | glomerular | [1] |
| 9208 | LRRFIP1 | leucine rich repeat (in FLII) interacting protein 1 | podocyte | [1,2] |
| 9935 | MAFB | v-maf musculoaponeurotic fibrosarcoma oncogene homolog B (avian) | podocyte | [1] |
| 9223 | MAGI1 | membrane associated guanylate kinase, WW and PDZ domain containing 1 | glomerular | [1] |
| 9863 | MAGI2 | membrane associated guanylate kinase, WW and PDZ domain containing 2 | glomerular | [1] |
| 84557 | MAP1LC3A | microtubule-associated protein 1 light chain 3 alpha | podocyte | [8] |
| 9053 | MAP7 | microtubule-associated protein 7 | glomerular | [1] |
| 4137 | MAPT | microtubule-associated protein tau | podocyte | [1,2] |
| 4192 | MDK | midkine (neurite growth-promoting factor 2) | glomerular | [1] |
| 4212 | MEIS2 | Meis homeobox 2 | mesangial | [1] |
| 4232 | MEST | mesoderm specific transcript homolog (mouse) | mesangial | [1] |
| 8972 | MGAM | maltase-glucoamylase (alpha-glucosidase) | podocyte | [1] |
| 4311 | MME (CD10) | membrane metallo-endopeptidase | podocyte | [9] |
| 4313 | MMP2 | matrix metallopeptidase 2 (gelatinase A, 72kDa gelatinase, 72kDa type IV collagenase) | glomerular | [1] |
| 8510 | MMP23B | matrix metallopeptidase 23B | podocyte | [1,2] |
| 4478 | MSN | moesin | glomerular | [1] |
| 4627 | MYH9 | myosin, heavy chain 9, non-muscle | glomerular | [1] |
| 4653 | MYOC | myocilin, trabecular meshwork inducible glucocorticoid response | podocyte | [1] |
| 79731 | NARS2 | asparaginyl-tRNA synthetase 2, mitochondrial (putative) | podocyte | [1] |
| 10763 | NES | nestin | podocyte | [10] |
| 4811 | NID1 | nidogen 1 | glomerular | [1] |
| 22795 | NID2 | nidogen 2 (osteonidogen) | glomerular | [1] |
| 4846 | NOS3 | nitric oxide synthase 3 (endothelial cell) | glomerular | [1] |
| 4851 | NOTCH1 | Notch homolog 1, translocation-associated (Drosophila) | glomerular | [1] |
| 4853 | NOTCH2 | Notch homolog 2 (Drosophila) | glomerular | [1] |
| 4855 | NOTCH4 | Notch homolog 4 (Drosophila) | glomerular | [1] |
| 4868 | NPHS1 | nephrosis 1, congenital, Finnish type (nephrin) | podocyte | [1] |
| 7827 | NPHS2 | nephrosis 2, idiopathic, steroid-resistant (podocin) | podocyte | [1] |
| 8829 | NRP1 | neuropilin 1 | podocyte | [1] |
| 8828 | NRP2 | neuropilin 2 | glomerular | [1] |
| 23022 | PALLD | palladin, cytoskeletal associated protein | podocyte | [1,2] |
| 51294 | PCDH12 | protocadherin 12 | glomerular | [1] |
| 5046 | PCSK6 | proprotein convertase subtilisin/kexin type 6 | podocyte | [1,2] |
| 5155 | PDGFB | platelet-derived growth factor beta polypeptide (simian sarcoma viral (v-sis) oncogene homolog) | endothelial | [1] |
| 80310 | PDGFD | platelet derived growth factor D | glomerular | [1] |
| 5159 | PDGFRB | platelet-derived growth factor receptor, beta polypeptide | glomerular | [1] |
| 10630 | PDPN | podoplanin | podocyte | [11] |
| 5175 | PECAM1 | platelet/endothelial cell adhesion molecule | glomerular | [1] |
| 5310 | PKD1 | polycystic kidney disease 1 (autosomal dominant) | glomerular | [1] |
| 22925 | PLA2R1 | phospholipase A2 receptor 1, 180kDa | glomerular | [12] |
| 5329 | PLAUR | plasminogen activator, urokinase receptor | podocyte | [2] |
| 51196 | PLCE1 | phospholipase C, epsilon 1 | podocyte | [13] |
| 127435 | PODN | podocan | glomerular | [1] |
| 5420 | PODXL | podocalyxin-like | podocyte | [1] |
| 8611 | PPAP2A | phosphatidic acid phosphatase type 2A | mesangial | [1] |
| 5742 | PTGS1 | prostaglandin-endoperoxide synthase 1 (prostaglandin G/H synthase and cyclooxygenase) | mesangial | [1] |
| 5745 | PTHR1 | parathyroid hormone 1 receptor | glomerular | [1] |
| 5747 | PTK2 | PTK2 protein tyrosine kinase 2 | glomerular | [1] |
| 5800 | PTPRO | protein tyrosine phosphatase, receptor type, O | podocyte | [2] |
| 5829 | PXN | paxillin | glomerular | [1] |
| 5864 | RAB3A | RAB3A, member RAS oncogene family | glomerular | [1] |
| 5865 | RAB3B | RAB3B, member RAS oncogene family | podocyte | [1,2] |
| 9649 | RALGPS1 | Ral GEF with PH domain and SH3 binding motif 1 | podocyte | [1] |
| 387496 | RASL11A | RAS-like, family 11, member A | podocyte | [1,2] |
| 5947 | RBP1 | retinol binding protein 1, cellular | glomerular | [1] |
| 5962 | RDX | radixin | glomerular | [1] |
| 114822 | RHPN1 | rhophilin, Rho GTPase binding protein 1 | podocyte | [1] |
| 6092 | ROBO2 | roundabout, axon guidance receptor, homolog 2 (Drosophila) | podocyte | [1] |
| 22895 | RPH3A | rabphilin 3A homolog (mouse) | glomerular | [1] |
| 3921 | RPSA | ribosomal protein SA | glomerular | [1] |
| 57142 | RTN4 | reticulon 4 | podocyte | [1] |
| 29970 | SCHIP1 | schwannomin interacting protein 1 | podocyte | [1] |
| 57147 | SCYL3 | SCY1-like 3 (S. cerevisiae) | podocyte | [1] |
| 6385 | SDC4 | syndecan 4 | glomerular | [1] |
| 10371 | SEMA3A | sema domain, immunoglobulin domain (Ig), short basic domain, secreted, (semaphorin) 3A | glomerular | [1] |
| 6405 | SEMA3F | sema domain, immunoglobulin domain (Ig), short basic domain, secreted, (semaphorin) 3F | glomerular | [1] |
| 56920 | SEMA3G | sema domain, immunoglobulin domain (Ig), short basic domain, secreted, (semaphorin) 3G | podocyte | [1,2] |
| 89778 | SERPINB11 | serpin peptidase inhibitor, clade B (ovalbumin), member 11 (gene/pseudogene) | glomerular | [1] |
| 5270 | SERPINE2 | serpin peptidase inhibitor, clade E (nexin, plasminogen activator inhibitor type 1), member 2 | mesangial | [1] |
| 6423 | SFRP2 | secreted frizzled-related protein 2 | mesangial | [1] |
| 9467 | SH3BP5 | SH3-domain binding protein 5 (BTK-associated) | glomerular | [1] |
| 152573 | SHISA3 | shisa homolog 3 (Xenopus laevis) | podocyte | [1] |
| 9351 | SLC9A3R2 | solute carrier family 9 (sodium/hydrogen exchanger), member 3 regulator 2 | glomerular | [1] |
| 4086 | SMAD1 | SMAD family member 1 | glomerular | [1] |
| 4087 | SMAD2 | SMAD family member 2 | glomerular | [1] |
| 4088 | SMAD3 | SMAD family member 3 | glomerular | [1] |
| 4089 | SMAD4 | SMAD family member 4 | glomerular | [1] |
| 4091 | SMAD6 | SMAD family member 6 | glomerular | [1] |
| 4092 | SMAD7 | SMAD family member 7 | glomerular | [1] |
| 9627 | SNCAIP | synuclein, alpha interacting protein | podocyte | [1,2] |
| 6678 | SPARC | secreted protein, acidic, cysteine-rich (osteonectin) | glomerular | [1] |
| 11346 | SYNPO | synaptopodin | podocyte | [1] |
| 6943 | TCF21 | transcription factor 21 | podocyte | [1] |
| 7010 | TEK | TEK tyrosine kinase, endothelial | endothelial | [1] |
| 7040 | TGFB1 | transforming growth factor, beta 1 | glomerular | [1] |
| 221981 | THSD7A | thrombospondin, type I, domain containing 7A | podocyte | [1] |
| 7075 | TIE1 | tyrosine kinase with immunoglobulin-like and EGF-like domains 1 | glomerular | [1] |
| 7078 | TIMP3 | TIMP metallopeptidase inhibitor 3 | podocyte | [1] |
| 7082 | TJP1 | tight junction protein 1 (zona occludens 1) | podocyte | [1] |
| 7094 | TLN1 | talin 1 | glomerular | [1] |
| 7145 | TNS1 | tensin 1 | glomerular | [1] |
| 64759 | TNS3 | tensin 3 | mesangial | [14] |
| 7148 | TNXB | tenascin XB | glomerular | [1] |
| 7157 | TP53 | tumor protein p53 | glomerular | [1] |
| 28951 | TRIB2 | tribbles homolog 2 (Drosophila) | podocyte | [1] |
| 7311 | UBA52 | ubiquitin A-52 residue ribosomal protein fusion product 1 | podocyte | [1] |
| 7345 | UCHL1 | ubiquitin carboxyl-terminal esterase L1 (ubiquitin thiolesterase) | podocyte | [15] |
| 55230 | USP40 | ubiquitin specific peptidase 40 | podocyte | [1] |
| 7402 | UTRN | utrophin | podocyte | [1] |
| 7414 | VCL | vinculin | glomerular | [1] |
| 7422 | VEGFA | vascular endothelial growth factor A | glomerular | [1] |
| 7431 | VIM | vimentin | glomerular | [1] |
| 7448 | VTN | vitronectin | glomerular | [1] |
| 7490 | WT1 | Wilms tumor 1 | podocyte | [1] |
| 126374 | WTIP | Wilms tumor 1 interacting protein | glomerular | [1] |
| 83719 | YPEL3 | yippee-like 3 (Drosophila) | podocyte | [1] |

1. Takemoto M, He L, Norlin J, Patrakka J, Xiao Z, et al. (2006) Large-scale identification of genes implicated in kidney glomerulus development and function. Embo J 25: 1160-1174.

2. Sun Y, He L, Takemoto M, Patrakka J, Pikkarainen T, et al. (2009) Glomerular transcriptome changes associated with lipopolysaccharide-induced proteinuria. Am J Nephrol 29: 558-570.

3. Reiser J, von Gersdorff G, Loos M, Oh J, Asanuma K, et al. (2004) Induction of B7-1 in podocytes is associated with nephrotic syndrome. J Clin Invest 113: 1390-1397.

4. Arnemann J, Sultani O, Hasgun D, Coerdt W (2006) T-/H-cadherin (CDH13): a new marker for differentiating podocytes. Virchows Arch 448: 160-164.

5. Barisoni L, Kriz W, Mundel P, D'Agati V (1999) The dysregulated podocyte phenotype: a novel concept in the pathogenesis of collapsing idiopathic focal segmental glomerulosclerosis and HIV-associated nephropathy. J Am Soc Nephrol 10: 51-61.

6. Patrakka J, Xiao Z, Nukui M, Takemoto M, He L, et al. (2007) Expression and subcellular distribution of novel glomerulus-associated proteins dendrin, ehd3, sh2d4a, plekhh2, and 2310066E14Rik. J Am Soc Nephrol 18: 689-697.

7. Inoue T, Yaoita E, Kurihara H, Shimizu F, Sakai T, et al. (2001) FAT is a component of glomerular slit diaphragms. Kidney Int 59: 1003-1012.

8. Asanuma K, Tanida I, Shirato I, Ueno T, Takahara H, et al. (2003) MAP-LC3, a promising autophagosomal marker, is processed during the differentiation and recovery of podocytes from PAN nephrosis. Faseb J 17: 1165-1167.

9. Dijkman HB, Weening JJ, Smeets B, Verrijp KC, van Kuppevelt TH, et al. (2006) Proliferating cells in HIV and pamidronate-associated collapsing focal segmental glomerulosclerosis are parietal epithelial cells. Kidney Int 70: 338-344.

10. Chen J, Boyle S, Zhao M, Su W, Takahashi K, et al. (2006) Differential expression of the intermediate filament protein nestin during renal development and its localization in adult podocytes. J Am Soc Nephrol 17: 1283-1291.

11. Matsui K, Breiteneder-Geleff S, Kerjaschki D (1998) Epitope-specific antibodies to the 43-kD glomerular membrane protein podoplanin cause proteinuria and rapid flattening of podocytes. J Am Soc Nephrol 9: 2013-2026.

12. Beck S, Beck G, Ostendorf T, Floege J, Lambeau G, et al. (2006) Upregulation of group IB secreted phospholipase A(2) and its M-type receptor in rat ANTI-THY-1 glomerulonephritis. Kidney Int 70: 1251-1260.

13. Hinkes B, Wiggins RC, Gbadegesin R, Vlangos CN, Seelow D, et al. (2006) Positional cloning uncovers mutations in PLCE1 responsible for a nephrotic syndrome variant that may be reversible. Nat Genet 38: 1397-1405.

14. Yamashita M, Horikoshi S, Asanuma K, Takahara H, Shirato I, et al. (2004) Tensin is potentially involved in extracellular matrix production in mesangial cells. Histochem Cell Biol 121: 245-254.

15. Diomedi-Camassei F, Rava L, Lerut E, Callea F, Van Damme B (2005) Protein gene product 9.5 and ubiquitin are expressed in metabolically active epithelial cells of normal and pathologic human kidney. Nephrol Dial Transplant 20: 2714-2719.
