# Supplementary material for: Systematic Analysis of a Novel Human Renal Glomerulus-Enriched Gene Expression Dataset
Source: PLoS One. 2010 Jul 12;5(7):e11545. doi: 10.1371/journal.pone.0011545 (PMC2902524; doi:10.1371/journal.pone.0011545)
Supplement: Table S4 — Prominent biological aspects found by DAVID analysis (0.36 MB DOC) [file pone.0011545.s005.doc]

**Table S4**

| **GO Term** | **GO ID** | **Genes involved in the GO category** | **involved genes/**  **total genes (%)** | **p-value**  **(Ease-Score DAVID)** | **Fold enrichment** |
| --- | --- | --- | --- | --- | --- |
|  |  |  |  |  |  |
| **Angiogenesis** |  |  |  |  |  |
| angiogenesis | GO:0001525 | 17 | 2.51 | 3.03E-03 | 2.28 |
| blood vessel development | GO:0001568 | 36 | 5.32 | 2.50E-08 | 2.88 |
| blood vessel morphogenesis | GO:0048514 | 29 | 4.28 | 3.15E-06 | 2.68 |
| patterning of blood vessels | GO:0001569 | 5 | 0.74 | 2.08E-02 | 4.60 |
| regulation of angiogenesis | GO:0045765 | 8 | 1.18 | 3.96E-02 | 2.49 |
| vasculature development | GO:0001944 | 39 | 5.76 | 1.22E-09 | 3.04 |
| vasculogenesis | GO:0001570 | 11 | 1.62 | 3.12E-05 | 5.19 |
|  |  |  |  |  |  |
|  |  |  |  |  |  |
| **Cell Cycle** |  |  |  |  |  |
| cell cycle arrest | GO:0007050 | 14 | 2.07 | 1.08E-03 | 2.86 |
| negative regulation of cell cycle | GO:0045786 | 11 | 1.62 | 7.03E-03 | 2.70 |
| negative regulation of cell cycle process | GO:0010948 | 6 | 0.89 | 6.89E-03 | 4.80 |
| negative regulation of mitotic cell cycle | GO:0045930 | 5 | 0.74 | 2.08E-02 | 4.60 |
| negative regulation of S phase of mitotic cell cycle | GO:0045749 | 4 | 0.59 | 7.25E-03 | 9.20 |
| regulation of S phase | GO:0033261 | 5 | 0.74 | 1.73E-02 | 4.84 |
| regulation of S phase of mitotic cell cycle | GO:0007090 | 5 | 0.74 | 7.25E-03 | 6.13 |
|  |  |  |  |  |  |
|  |  |  |  |  |  |
| **Cell death** |  |  |  |  |  |
| induction of programmed cell death | GO:0012502 | 24 | 3.55 | 4.18E-02 | 1.53 |
| negative regulation of apoptosis | GO:0043066 | 30 | 4.43 | 5.46E-03 | 1.70 |
| negative regulation of cell death | GO:0060548 | 30 | 4.43 | 6.82E-03 | 1.68 |
| negative regulation of programmed cell death | GO:0043069 | 30 | 4.43 | 6.46E-03 | 1.68 |
| regulation of apoptosis | GO:0042981 | 60 | 8.86 | 8.56E-04 | 1.53 |
| regulation of cell death | GO:0010941 | 60 | 8.86 | 1.17E-03 | 1.51 |
| regulation of programmed cell death | GO:0043067 | 60 | 8.86 | 1.06E-03 | 1.52 |
|  |  |  |  |  |  |
|  |  |  |  |  |  |
| **Cytoskeleton** |  |  |  |  |  |
| actin cytoskeleton organization | GO:0030036 | 23 | 3.40 | 7.70E-04 | 2.19 |
| actin filament-based process | GO:0030029 | 24 | 3.55 | 9.00E-04 | 2.12 |
| cytoplasmic microtubule organization | GO:0031122 | 3 | 0.44 | 3.81E-02 | 9.20 |
| cytoskeleton organization | GO:0007010 | 36 | 5.32 | 9.71E-04 | 1.78 |
| negative regulation of cytoskeleton organization | GO:0051494 | 8 | 1.18 | 1.75E-02 | 2.94 |
| positive regulation of microtubule polymerization | GO:0031116 | 4 | 0.59 | 1.43E-02 | 7.36 |
| positive regulation of microtubule polymerization or depolymerization | GO:0031112 | 4 | 0.59 | 1.89E-02 | 6.69 |
| regulation of actin filament-based process | GO:0032970 | 10 | 1.48 | 3.09E-02 | 2.27 |
| regulation of cytoskeleton organization | GO:0051493 | 16 | 2.36 | 1.75E-03 | 2.49 |
| regulation of microtubule cytoskeleton organization | GO:0070507 | 7 | 1.03 | 1.21E-02 | 3.58 |
| regulation of microtubule polymerization | GO:0031113 | 4 | 0.59 | 1.89E-02 | 6.69 |
| regulation of microtubule polymerization or depolymerization | GO:0031110 | 7 | 1.03 | 1.82E-03 | 5.15 |
| regulation of microtubule-based process | GO:0032886 | 8 | 1.18 | 6.00E-03 | 3.59 |
|  |  |  |  |  |  |
|  |  |  |  |  |  |
| **DNA/RNA** |  |  |  |  |  |
| chromatin assembly | GO:0031497 | 10 | 1.48 | 6.59E-03 | 2.92 |
| chromatin assembly or disassembly | GO:0006333 | 12 | 1.77 | 1.94E-02 | 2.21 |
| DNA packaging | GO:0006323 | 10 | 1.48 | 4.30E-02 | 2.14 |
| negative regulation of gene expression | GO:0010629 | 36 | 5.32 | 1.20E-02 | 1.53 |
| negative regulation of nucleobase, nucleoside, nucleotide and nucleic acid metabolic process | GO:0045934 | 37 | 5.47 | 1.15E-02 | 1.52 |
| negative regulation of transcription | GO:0016481 | 35 | 5.17 | 5.93E-03 | 1.61 |
| nucleosome assembly | GO:0006334 | 10 | 1.48 | 5.31E-03 | 3.01 |
| nucleosome organization | GO:0034728 | 10 | 1.48 | 1.18E-02 | 2.67 |
|  |  |  |  |  |  |
|  |  |  |  |  |  |
| **Immune response/Inflammation** |  |  |  |  |  |
| activation of plasma proteins involved in acute inflammatory response | GO:0002541 | 8 | 1.18 | 3.88E-03 | 3.87 |
| complement activation | GO:0006956 | 7 | 1.03 | 1.37E-02 | 3.48 |
| humoral immune response | GO:0006959 | 9 | 1.33 | 4.65E-02 | 2.24 |
|  |  |  |  |  |  |
|  |  |  |  |  |  |
|  |  |  |  |  |  |
| **Metabolism** |  |  |  |  |  |
| aminoglycan metabolic process | GO:0006022 | 9 | 1.33 | 4.50E-03 | 3.38 |
| chondroitin sulfate metabolic process | GO:0030204 | 4 | 0.59 | 1.89E-02 | 6.69 |
| chondroitin sulfate proteoglycan metabolic process | GO:0050654 | 6 | 0.89 | 6.15E-04 | 7.88 |
| glycoprotein metabolic process | GO:0009100 | 18 | 2.66 | 8.17E-03 | 2.01 |
| glycosaminoglycan metabolic process | GO:0030203 | 7 | 1.03 | 2.47E-02 | 3.07 |
| negative regulation of biosynthetic process | GO:0009890 | 45 | 6.65 | 9.27E-04 | 1.66 |
| negative regulation of cellular biosynthetic process | GO:0031327 | 42 | 6.20 | 3.39E-03 | 1.58 |
| negative regulation of cellular metabolic process | GO:0031324 | 53 | 7.83 | 1.83E-03 | 1.53 |
| negative regulation of macromolecule biosynthetic process | GO:0010558 | 43 | 6.35 | 1.16E-03 | 1.66 |
| negative regulation of macromolecule metabolic process | GO:0010605 | 53 | 7.83 | 2.32E-03 | 1.52 |
| negative regulation of nitrogen compound metabolic process | GO:0051172 | 38 | 5.61 | 8.40E-03 | 1.54 |
| neurotransmitter receptor metabolic process | GO:0045213 | 3 | 0.44 | 8.51E-03 | 18.39 |
| polysaccharide biosynthetic process | GO:0000271 | 6 | 0.89 | 4.80E-02 | 2.98 |
| polysaccharide metabolic process | GO:0005976 | 12 | 1.77 | 7.20E-03 | 2.54 |
| proteoglycan metabolic process | GO:0006029 | 8 | 1.18 | 1.66E-03 | 4.46 |
| receptor metabolic process | GO:0043112 | 6 | 0.89 | 1.86E-02 | 3.81 |
|  |  |  |  |  |  |
|  |  |  |  |  |  |
| **Muscle** |  |  |  |  |  |
| muscle cell differentiation | GO:0042692 | 16 | 2.36 | 1.03E-03 | 2.63 |
| muscle organ development | GO:0007517 | 21 | 3.10 | 3.00E-03 | 2.05 |
| muscle tissue development | GO:0060537 | 14 | 2.07 | 1.00E-02 | 2.22 |
| positive regulation of smooth muscle cell proliferation | GO:0048661 | 6 | 0.89 | 2.44E-02 | 3.56 |
| regulation of smooth muscle cell proliferation | GO:0048660 | 7 | 1.03 | 3.69E-02 | 2.80 |
| striated muscle cell development | GO:0055002 | 7 | 1.03 | 4.04E-02 | 2.74 |
| striated muscle cell differentiation | GO:0051146 | 12 | 1.77 | 4.17E-03 | 2.72 |
| striated muscle tissue development | GO:0014706 | 12 | 1.77 | 3.61E-02 | 2.01 |
|  |  |  |  |  |  |
|  |  |  |  |  |  |
| **Nervous System** |  |  |  |  |  |
| axon guidance | GO:0007411 | 11 | 1.62 | 2.71E-02 | 2.20 |
| axonogenesis | GO:0007409 | 20 | 2.95 | 1.23E-03 | 2.27 |
| cell morphogenesis involved in neuron differentiation | GO:0048667 | 22 | 3.25 | 5.11E-04 | 2.31 |
| nervous system development | GO:0007399 | 82 | 12.11 | 8.43E-06 | 1.63 |
| neurogenesis | GO:0022008 | 45 | 6.65 | 1.57E-03 | 1.62 |
| neuron development | GO:0048666 | 30 | 4.43 | 1.06E-03 | 1.90 |
| neuron differentiation | GO:0030182 | 34 | 5.02 | 3.06E-03 | 1.70 |
| neuron projection development | GO:0031175 | 23 | 3.40 | 3.67E-03 | 1.94 |
| neuron projection morphogenesis | GO:0048812 | 23 | 3.40 | 3.17E-04 | 2.34 |
| regulation of neurological system process | GO:0031644 | 14 | 2.07 | 4.47E-02 | 1.81 |
| regulation of synaptic transmission, GABAergic | GO:0032228 | 5 | 0.74 | 1.16E-02 | 5.41 |
|  |  |  |  |  |  |
|  |  |  |  |  |  |
| **Proteins/Enzymes** |  |  |  |  |  |
| dephosphorylation | GO:0016311 | 13 | 1.92 | 4.07E-02 | 1.90 |
| negative regulation of protein complex disassembly | GO:0043242 | 7 | 1.03 | 1.56E-02 | 3.39 |
| peptide cross-linking | GO:0018149 | 5 | 0.74 | 2.88E-02 | 4.18 |
| peptide cross-linking via chondroitin 4-sulfate glycosaminoglycan | GO:0019800 | 3 | 0.44 | 1.64E-02 | 13.79 |
| positive regulation of protein polymerization | GO:0032273 | 5 | 0.74 | 2.46E-02 | 4.38 |
| protein maturation | GO:0051604 | 12 | 1.77 | 3.61E-02 | 2.01 |
| protein processing | GO:0016485 | 11 | 1.62 | 4.70E-02 | 2.00 |
| protein-DNA complex assembly | GO:0065004 | 10 | 1.48 | 9.83E-03 | 2.74 |
| regulation of caspase activity | GO:0043281 | 9 | 1.33 | 4.35E-02 | 2.27 |
| regulation of cellular protein metabolic process | GO:0032268 | 36 | 5.32 | 5.98E-03 | 1.60 |
| regulation of endopeptidase activity | GO:0052548 | 9 | 1.33 | 4.97E-02 | 2.21 |
| regulation of hydrolase activity | GO:0051336 | 26 | 3.84 | 1.36E-02 | 1.66 |
| regulation of peptidase activity | GO:0052547 | 10 | 1.48 | 2.68E-02 | 2.33 |
| regulation of protein amino acid phosphorylation | GO:0001932 | 15 | 2.22 | 4.02E-02 | 1.79 |
| regulation of protein complex assembly | GO:0043254 | 10 | 1.48 | 2.68E-02 | 2.33 |
| regulation of protein complex disassembly | GO:0043244 | 7 | 1.03 | 4.41E-02 | 2.68 |
| regulation of protein metabolic process | GO:0051246 | 42 | 6.20 | 2.09E-03 | 1.63 |
| regulation of protein modification process | GO:0031399 | 23 | 3.40 | 2.98E-02 | 1.60 |
| regulation of protein polymerization | GO:0032271 | 8 | 1.18 | 3.36E-02 | 2.58 |
|  |  |  |  |  |  |
|  |  |  |  |  |  |
| **Signal transduction/Pathways** |  |  |  |  |  |
| elevation of cytosolic calcium ion concentration during G-protein signaling, coupled to IP3 second messenger (phospholipase C activating) | GO:0051482 | 4 | 0.59 | 2.42E-02 | 6.13 |
| enzyme linked receptor protein signaling pathway | GO:0007167 | 37 | 5.47 | 2.80E-05 | 2.11 |
| integrin-mediated signaling pathway | GO:0007229 | 12 | 1.77 | 3.30E-04 | 3.68 |
| intracellular receptor-mediated signaling pathway | GO:0030522 | 9 | 1.33 | 3.51E-02 | 2.36 |
| negative regulation of signal transduction | GO:0009968 | 18 | 2.66 | 3.62E-02 | 1.70 |
| platelet-derived growth factor receptor signaling pathway | GO:0048008 | 5 | 0.74 | 1.16E-02 | 5.41 |
| positive regulation of signal transduction | GO:0009967 | 22 | 3.25 | 4.73E-02 | 1.54 |
| regulation of G-protein coupled receptor protein signaling pathway | GO:0008277 | 7 | 1.03 | 4.41E-02 | 2.68 |
| regulation of Ras protein signal transduction | GO:0046578 | 16 | 2.36 | 2.22E-02 | 1.89 |
| regulation of signal transduction | GO:0009966 | 71 | 10.49 | 1.71E-06 | 1.79 |
| regulation of small GTPase mediated signal transduction | GO:0051056 | 21 | 3.10 | 2.82E-03 | 2.07 |
| regulation of Wnt receptor signaling pathway | GO:0030111 | 8 | 1.18 | 6.87E-03 | 3.50 |
| transmembrane receptor protein tyrosine kinase signaling pathway | GO:0007169 | 26 | 3.84 | 1.90E-04 | 2.27 |
| Wnt receptor signaling pathway | GO:0016055 | 14 | 2.07 | 9.35E-03 | 2.24 |
|  |  |  |  |  |  |
|  |  |  |  |  |  |
| **Others** |  |  |  |  |  |
| anatomical structure formation involved in morphogenesis | GO:0048646 | 31 | 4.58 | 1.33E-03 | 1.85 |
| anatomical structure morphogenesis | GO:0009653 | 98 | 14.48 | 4.19E-08 | 1.73 |
| biological adhesion | GO:0022610 | 52 | 7.68 | 4.91E-04 | 1.63 |
| blood circulation | GO:0008015 | 18 | 2.66 | 1.50E-02 | 1.88 |
| bone development | GO:0060348 | 12 | 1.77 | 3.82E-02 | 1.99 |
| branching morphogenesis of a tube | GO:0048754 | 11 | 1.62 | 1.71E-03 | 3.26 |
| cell adhesion | GO:0007155 | 52 | 7.68 | 4.91E-04 | 1.63 |
| cell development | GO:0048468 | 56 | 8.27 | 8.83E-06 | 1.86 |
| cell differentiation | GO:0030154 | 116 | 17.13 | 4.38E-07 | 1.57 |
| cell migration | GO:0016477 | 23 | 3.40 | 1.80E-02 | 1.69 |
| cell morphogenesis | GO:0000902 | 32 | 4.73 | 4.38E-04 | 1.96 |
| cell morphogenesis involved in differentiation | GO:0000904 | 26 | 3.84 | 1.51E-04 | 2.30 |
| cell motility | GO:0048870 | 23 | 3.40 | 3.82E-02 | 1.56 |
| cell motion | GO:0006928 | 38 | 5.61 | 2.48E-03 | 1.66 |
| cell part morphogenesis | GO:0032990 | 24 | 3.55 | 1.60E-03 | 2.03 |
| cell projection morphogenesis | GO:0048858 | 24 | 3.55 | 7.88E-04 | 2.14 |
| cell projection organization | GO:0030030 | 28 | 4.14 | 1.06E-02 | 1.66 |
| cell-cell junction organization | GO:0045216 | 6 | 0.89 | 2.44E-02 | 3.56 |
| cellular component morphogenesis | GO:0032989 | 36 | 5.32 | 1.43E-04 | 1.98 |
| cellular developmental process | GO:0048869 | 118 | 17.43 | 1.16E-06 | 1.53 |
| circulatory system process | GO:0003013 | 18 | 2.66 | 1.50E-02 | 1.88 |
| developmental growth | GO:0048589 | 11 | 1.62 | 8.45E-03 | 2.63 |
| endocytosis | GO:0006897 | 22 | 3.25 | 2.19E-03 | 2.06 |
| extracellular matrix organization | GO:0030198 | 13 | 1.92 | 5.15E-03 | 2.52 |
| extracellular structure organization | GO:0043062 | 19 | 2.81 | 7.78E-04 | 2.43 |
| heart development | GO:0007507 | 19 | 2.81 | 2.01E-02 | 1.78 |
| interspecies interaction between organisms | GO:0044419 | 23 | 3.40 | 4.24E-02 | 1.54 |
| kidney development | GO:0001822 | 12 | 1.77 | 7.20E-03 | 2.54 |
| localization of cell | GO:0051674 | 23 | 3.40 | 3.82E-02 | 1.56 |
| lung alveolus development | GO:0048286 | 5 | 0.74 | 2.08E-02 | 4.60 |
| lymph vessel development | GO:0001945 | 3 | 0.44 | 3.81E-02 | 9.20 |
| membrane invagination | GO:0010324 | 22 | 3.25 | 2.19E-03 | 2.06 |
| mesenchymal cell development | GO:0014031 | 7 | 1.03 | 4.04E-02 | 2.74 |
| mesenchymal cell differentiation | GO:0048762 | 7 | 1.03 | 4.04E-02 | 2.74 |
| mesenchyme development | GO:0060485 | 7 | 1.03 | 4.41E-02 | 2.68 |
| morphogenesis of a branching structure | GO:0001763 | 11 | 1.62 | 4.28E-03 | 2.89 |
| negative regulation of biological process | GO:0048519 | 135 | 19.94 | 5.94E-08 | 1.55 |
| negative regulation of cell communication | GO:0010648 | 22 | 3.25 | 9.26E-03 | 1.82 |
| negative regulation of cell motion | GO:0051271 | 8 | 1.18 | 3.65E-02 | 2.54 |
| negative regulation of cell proliferation | GO:0008285 | 34 | 5.02 | 5.52E-04 | 1.88 |
| negative regulation of cellular process | GO:0048523 | 129 | 19.05 | 1.23E-08 | 1.62 |
| negative regulation of developmental process | GO:0051093 | 21 | 3.10 | 2.51E-02 | 1.68 |
| negative regulation of organelle organization | GO:0010639 | 10 | 1.48 | 2.14E-02 | 2.42 |
| organ development | GO:0048513 | 118 | 17.43 | 2.55E-05 | 1.44 |
| pattern specification process | GO:0007389 | 21 | 3.10 | 1.95E-02 | 1.72 |
| positive regulation of biological process | GO:0048518 | 139 | 20.53 | 7.06E-06 | 1.42 |
| positive regulation of cell communication | GO:0010647 | 27 | 3.99 | 9.19E-03 | 1.69 |
| positive regulation of cell migration | GO:0030335 | 11 | 1.62 | 1.91E-02 | 2.33 |
| positive regulation of cell motion | GO:0051272 | 11 | 1.62 | 3.29E-02 | 2.13 |
| positive regulation of cell proliferation | GO:0008284 | 30 | 4.43 | 4.55E-02 | 1.43 |
| positive regulation of epithelial cell proliferation | GO:0050679 | 6 | 0.89 | 3.12E-02 | 3.34 |
| positive regulation of ion transport | GO:0043270 | 6 | 0.89 | 4.80E-02 | 2.98 |
| positive regulation of locomotion | GO:0040017 | 11 | 1.62 | 3.29E-02 | 2.13 |
| positive regulation of mesenchymal cell proliferation | GO:0002053 | 4 | 0.59 | 4.44E-02 | 4.90 |
| regulation of anatomical structure morphogenesis | GO:0022603 | 24 | 3.55 | 3.90E-04 | 2.25 |
| regulation of biological quality | GO:0065008 | 93 | 13.74 | 4.87E-03 | 1.31 |
| regulation of blood coagulation | GO:0030193 | 7 | 1.03 | 9.12E-03 | 3.79 |
| regulation of body fluid levels | GO:0050878 | 16 | 2.36 | 3.90E-03 | 2.30 |
| regulation of cell communication | GO:0010646 | 84 | 12.41 | 2.91E-07 | 1.76 |
| regulation of cell growth | GO:0001558 | 19 | 2.81 | 7.13E-03 | 1.99 |
| regulation of cell migration | GO:0030334 | 19 | 2.81 | 2.60E-03 | 2.18 |
| regulation of cell morphogenesis | GO:0022604 | 14 | 2.07 | 8.10E-03 | 2.28 |
| regulation of cell motion | GO:0051270 | 22 | 3.25 | 8.00E-04 | 2.24 |
| regulation of cell proliferation | GO:0042127 | 66 | 9.75 | 3.61E-05 | 1.67 |
| regulation of cell shape | GO:0008360 | 9 | 1.33 | 2.24E-03 | 3.76 |
| regulation of cellular component biogenesis | GO:0044087 | 16 | 2.36 | 3.62E-03 | 2.32 |
| regulation of cellular component organization | GO:0051128 | 42 | 6.20 | 8.00E-05 | 1.91 |
| regulation of coagulation | GO:0050818 | 7 | 1.03 | 1.56E-02 | 3.39 |
| regulation of developmental process | GO:0050793 | 54 | 7.98 | 2.91E-04 | 1.65 |
| regulation of epithelial cell proliferation | GO:0050678 | 10 | 1.48 | 6.59E-03 | 2.92 |
| regulation of growth | GO:0040008 | 27 | 3.99 | 1.13E-02 | 1.67 |
| regulation of locomotion | GO:0040012 | 20 | 2.95 | 3.64E-03 | 2.07 |
| regulation of mesenchymal cell proliferation | GO:0010464 | 5 | 0.74 | 9.26E-03 | 5.75 |
| regulation of organelle organization | GO:0033043 | 20 | 2.95 | 8.19E-03 | 1.92 |
| regulation of response to external stimulus | GO:0032101 | 17 | 2.51 | 6.46E-03 | 2.11 |
| regulation of response to stimulus | GO:0048583 | 36 | 5.32 | 7.41E-03 | 1.58 |
| regulation of response to stress | GO:0080134 | 23 | 3.40 | 1.72E-02 | 1.69 |
| regulation of system process | GO:0044057 | 27 | 3.99 | 8.09E-03 | 1.71 |
| response to external stimulus | GO:0009605 | 60 | 8.86 | 9.16E-03 | 1.38 |
| response to extracellular stimulus | GO:0009991 | 18 | 2.66 | 4.43E-02 | 1.66 |
| response to mechanical stimulus | GO:0009612 | 8 | 1.18 | 2.36E-02 | 2.78 |
| response to nutrient levels | GO:0031667 | 17 | 2.51 | 3.71E-02 | 1.73 |
| response to wounding | GO:0009611 | 42 | 6.20 | 1.75E-03 | 1.64 |
| skeletal system development | GO:0001501 | 24 | 3.55 | 3.80E-02 | 1.54 |
| tissue development | GO:0009888 | 51 | 7.53 | 5.31E-04 | 1.64 |
| tube development | GO:0035295 | 20 | 2.95 | 9.11E-03 | 1.90 |
| tube morphogenesis | GO:0035239 | 13 | 1.92 | 1.70E-02 | 2.15 |
| urogenital system development | GO:0001655 | 12 | 1.77 | 1.94E-02 | 2.21 |
| wound healing | GO:0042060 | 19 | 2.81 | 4.69E-03 | 2.07 |
