# Supplementary material for: Systematic Analysis of a Novel Human Renal Glomerulus-Enriched Gene Expression Dataset
Source: PLoS One. 2010 Jul 12;5(7):e11545. doi: 10.1371/journal.pone.0011545 (PMC2902524; doi:10.1371/journal.pone.0011545)
Supplement: Table S5 — DAVID Functional Annotation Cluster Analysis (0.13 MB DOC) [file pone.0011545.s006.doc]

Table S5

| **Annotation Cluster 1** | Enrichment Score: 7.34 | |  |  |  |  |
| --- | --- | --- | --- | --- | --- | --- |
| **Category** | **ID** | **Term** | **Count** | **%** | **PValue** | **Fold Enrichment** |
| GOTERM_BP_ALL | GO:0001944 | vasculature development | 39 | 5.76 | 1.22E-09 | 3.04 |
| GOTERM_BP_ALL | GO:0001568 | blood vessel development | 36 | 5.32 | 2.50E-08 | 2.88 |
| GOTERM_BP_ALL | GO:0048514 | blood vessel morphogenesis | 29 | 4.28 | 3.15E-06 | 2.68 |
|  |  |  |  |  |  |  |
| **Annotation Cluster 2** | Enrichment Score: 3.02 | |  |  |  |  |
| **Category** | **ID** | **Term** | **Count** | **%** | **PValue** | **Fold Enrichment** |
| GOTERM_BP_ALL | GO:0000904 | cell morphogenesis involved in differentiation | 26 | 3.84 | 1.51E-04 | 2.30 |
| GOTERM_BP_ALL | GO:0048812 | neuron projection morphogenesis | 23 | 3.40 | 3.17E-04 | 2.34 |
| GOTERM_BP_ALL | GO:0000902 | cell morphogenesis | 32 | 4.73 | 4.38E-04 | 1.96 |
| GOTERM_BP_ALL | GO:0048667 | cell morphogenesis involved in neuron differentiation | 22 | 3.25 | 5.11E-04 | 2.31 |
| GOTERM_BP_ALL | GO:0048858 | cell projection morphogenesis | 24 | 3.55 | 7.88E-04 | 2.14 |
| GOTERM_BP_ALL | GO:0048666 | neuron development | 30 | 4.43 | 1.06E-03 | 1.90 |
| GOTERM_BP_ALL | GO:0007409 | axonogenesis | 20 | 2.95 | 1.23E-03 | 2.27 |
| GOTERM_BP_ALL | GO:0032990 | cell part morphogenesis | 24 | 3.55 | 1.60E-03 | 2.03 |
| GOTERM_BP_ALL | GO:0031175 | neuron projection development | 23 | 3.40 | 3.67E-03 | 1.94 |
| GOTERM_BP_ALL | GO:0030030 | cell projection organization | 28 | 4.14 | 1.06E-02 | 1.66 |
|  |  |  |  |  |  |  |
| **Annotation Cluster 3** | Enrichment Score: 2.99 | |  |  |  |  |
| **Category** | **ID** | **Term** | **Count** | **%** | **PValue** | **Fold Enrichment** |
| GOTERM_BP_ALL | GO:0042981 | regulation of apoptosis | 60 | 8.86 | 8.56E-04 | 1.53 |
| GOTERM_BP_ALL | GO:0043067 | regulation of programmed cell death | 60 | 8.86 | 1.06E-03 | 1.52 |
| GOTERM_BP_ALL | GO:0010941 | regulation of cell death | 60 | 8.86 | 1.17E-03 | 1.51 |
|  |  |  |  |  |  |  |
| **Annotation Cluster 4** | Enrichment Score: 2.71 | |  |  |  |  |
| **Category** | **ID** | **Term** | **Count** | **%** | **PValue** | **Fold Enrichment** |
| GOTERM_BP_ALL | GO:0051270 | regulation of cell motion | 22 | 3.25 | 8.00E-04 | 2.24 |
| GOTERM_BP_ALL | GO:0030334 | regulation of cell migration | 19 | 2.81 | 2.60E-03 | 2.18 |
| GOTERM_BP_ALL | GO:0040012 | regulation of locomotion | 20 | 2.95 | 3.64E-03 | 2.07 |
|  |  |  |  |  |  |  |
| **Annotation Cluster 5** | Enrichment Score: 2.53 | |  |  |  |  |
| **Category** | **ID** | **Term** | **Count** | **%** | **PValue** | **Fold Enrichment** |
| GOTERM_BP_ALL | GO:0048666 | neuron development | 30 | 4.43 | 1.06E-03 | 1.90 |
| GOTERM_BP_ALL | GO:0022008 | neurogenesis | 45 | 6.65 | 1.57E-03 | 1.62 |
| GOTERM_BP_ALL | GO:0030182 | neuron differentiation | 34 | 5.02 | 3.06E-03 | 1.70 |
|  |  |  |  |  |  |  |
| **Annotation Cluster 6** | Enrichment Score: 2.46 | |  |  |  |  |
| **Category** | **ID** | **Term** | **Count** | **%** | **PValue** | **Fold Enrichment** |
| GOTERM_BP_ALL | GO:0009890 | negative regulation of biosynthetic process | 45 | 6.65 | 9.27E-04 | 1.66 |
| GOTERM_BP_ALL | GO:0010558 | negative regulation of macromolecule biosynthetic process | 43 | 6.35 | 1.16E-03 | 1.66 |
| GOTERM_BP_ALL | GO:0031324 | negative regulation of cellular metabolic process | 53 | 7.83 | 1.83E-03 | 1.53 |
| GOTERM_BP_ALL | GO:0009892 | negative regulation of metabolic process | 56 | 8.27 | 2.17E-03 | 1.50 |
| GOTERM_BP_ALL | GO:0010605 | negative regulation of macromolecule metabolic process | 53 | 7.83 | 2.32E-03 | 1.52 |
| GOTERM_BP_ALL | GO:0031327 | negative regulation of cellular biosynthetic process | 42 | 6.20 | 3.39E-03 | 1.58 |
| GOTERM_BP_ALL | GO:0016481 | negative regulation of transcription | 35 | 5.17 | 5.93E-03 | 1.61 |
| GOTERM_BP_ALL | GO:0051172 | negative regulation of nitrogen compound metabolic process | 38 | 5.61 | 8.40E-03 | 1.54 |
| GOTERM_BP_ALL | GO:0045934 | negative regulation of nucleobase, nucleoside, nucleotide and nucleic acid metabolic process | 37 | 5.47 | 1.15E-02 | 1.52 |
| GOTERM_BP_ALL | GO:0010629 | negative regulation of gene expression | 36 | 5.32 | 1.20E-02 | 1.53 |
|  |  |  |  |  |  |  |
| **Annotation Cluster 7** | Enrichment Score: 2.30 | |  |  |  |  |
| **Category** | **ID** | **Term** | **Count** | **%** | **PValue** | **Fold Enrichment** |
| GOTERM_BP_ALL | GO:0048754 | branching morphogenesis of a tube | 11 | 1.62 | 1.71E-03 | 3.26 |
| GOTERM_BP_ALL | GO:0001763 | morphogenesis of a branching structure | 11 | 1.62 | 4.28E-03 | 2.89 |
| GOTERM_BP_ALL | GO:0035239 | tube morphogenesis | 13 | 1.92 | 1.70E-02 | 2.15 |
|  |  |  |  |  |  |  |
| **Annotation Cluster 8** | Enrichment Score: 2.29 | |  |  |  |  |
| **Category** | **ID** | **Term** | **Count** | **%** | **PValue** | **Fold Enrichment** |
| GOTERM_BP_ALL | GO:0031110 | regulation of microtubule polymerization or depolymerization | 7 | 1.03 | 1.82E-03 | 5.15 |
| GOTERM_BP_ALL | GO:0032886 | regulation of microtubule-based process | 8 | 1.18 | 6.00E-03 | 3.59 |
| GOTERM_BP_ALL | GO:0070507 | regulation of microtubule cytoskeleton organization | 7 | 1.03 | 1.21E-02 | 3.58 |
|  |  |  |  |  |  |  |
| **Annotation Cluster 9** | Enrichment Score: 2.21 | |  |  |  |  |
| **Category** | **ID** | **Term** | **Count** | **%** | **PValue** | **Fold Enrichment** |
| GOTERM_BP_ALL | GO:0043066 | negative regulation of apoptosis | 30 | 4.43 | 5.46E-03 | 1.70 |
| GOTERM_BP_ALL | GO:0043069 | negative regulation of programmed cell death | 30 | 4.43 | 6.46E-03 | 1.68 |
| GOTERM_BP_ALL | GO:0060548 | negative regulation of cell death | 30 | 4.43 | 6.82E-03 | 1.68 |
|  |  |  |  |  |  |  |
| **Annotation Cluster 10** | Enrichment Score: 2.01 | |  |  |  |  |
| **Category** | **ID** | **Term** | **Count** | **%** | **PValue** | **Fold Enrichment** |
| GOTERM_BP_ALL | GO:0045749 | negative regulation of S phase of mitotic cell cycle | 4 | 0.59 | 7.25E-03 | 9.20 |
| GOTERM_BP_ALL | GO:0007090 | regulation of S phase of mitotic cell cycle | 5 | 0.74 | 7.25E-03 | 6.13 |
| GOTERM_BP_ALL | GO:0033261 | regulation of S phase | 5 | 0.74 | 1.73E-02 | 4.84 |
