# Supplementary material for: Systematic Analysis of a Novel Human Renal Glomerulus-Enriched Gene Expression Dataset
Source: PLoS One. 2010 Jul 12;5(7):e11545. doi: 10.1371/journal.pone.0011545 (PMC2902524; doi:10.1371/journal.pone.0011545)
Supplement: Table S6 — KEGG pathways (0.04 MB DOC) [file pone.0011545.s007.doc]

Table S6

| **KEGG_ID** | **Term** | **Count** | **%** | **P-Value** | **Fold Enrichment** | **Genes** |
| --- | --- | --- | --- | --- | --- | --- |
| hsa05410 | Hypertrophic cardiomyopathy (HCM) | 12 | 1.77 | 2.61E-03 | 2.86 | ATP2A2, CACNB2, DAG1, DMD, IGF1, ITGA3, ITGAV, ITGB3, ITGB5, TNNC1, TNNT2, TPM2 |
| hsa04144 | Endocytosis | 19 | 2.81 | 3.95E-03 | 2.07 | ARAP2, CBLB, EHD2, EHD3, EHD4, ERBB4, F2R, GRK5, HLA-E, HSPA2, IQSEC1, KDR, PARD3, PRKCI, PSD3, RAB31, SHGLB1, TGFBR2, WWP1 |
| hsa04810 | Regulation of actin cytoskeleton | 21 | 3.10 | 4.36E-03 | 1.96 | APC, ARGHEF12, DOCK1, F2R, FGF1, GSN, IQGAP1, IQGAP2, ITGA3, ITGAV, ITGB3, ITGB5, LIMK2, MYH9, MYL9, MYLK3, PDGFD, PDGFRB, ROCK1, RRAS, TIAM1 |
| hsa05414 | Dilated cardiomyopathy | 12 | 1.77 | 4.77E-03 | 2.65 | ATP2A2, CACNB2, DAG1, DMD, IGF1, ITGA3, ITGAV, ITGB3, ITGB5, TNNC1, TNNT2, TPM2 |
| hsa04510 | Focal adhesion | 20 | 2.95 | 7.51E-03 | 1.91 | AKT3, CAV1, COL3A1, COL4A4, DOCK1, FYN, IGF1, ITGA3, ITGAV, ITGB3, ITGB5, KDR, LAMB2, MYL9, MYLK3, PARVA, PDGD, PDGFRB, ROCK1, VEGFA |
| hsa04360 | Axon guidance | 14 | 2.07 | 9.05E-03 | 2.22 | ARHGEF12, DPYSL2, EFNB2, LIMK2, NCK2, ROBO1, ROCK1, SEMA3B, SEMA3C, SEMA3G, SEMA5A, SRGAP2 |
| hsa04610 | Complement and coagulation cascades | 10 | 1.48 | 1.03E-02 | 2.70 | C1R, C1S, CD55, CFH, CR1, F2R, F3, F5, PLAT, SERPING1 |
| hsa05412 | Arrhythmogenic right ventricular cardiomyopathy (ARVC) | 10 | 1.48 | 1.13E-02 | 2.66 | ATP2A2, CACNB2, DAG1, DMD, GJA1, ITGA3, ITGAV, ITGB3, ITGB5, TCF7L1 |
| hsa04512 | ECM-receptor interaction | 11 | 1.62 | 1.24E-02 | 2.46 | AGRN, CD47, COL3A1, COL4A4, DAG1, ITGA3, ITGAV, ITGB3, ITGB5, LAMB2, SDC2 |
| hsa04530 | Tight junction | 14 | 2.07 | 1.46E-02 | 2.10 | AKT3, CLDN5, MAGI2, MPP5, MYH9, MYL9, PARD3, PPP2R2B, PRKCH, PRKCI, RRAS, SPTAN1, TJP1, ZAK |
| hsa05222 | Small cell lung cancer | 11 | 1.62 | 1.58E-02 | 2.37 | AKT3, CDKN1B, COL4A4, IKBKB, ITGA3, ITGAV, LAMB2, PIAS1, RB1, TRAF3, TRAF5 |
| hsa04270 | Vascular smooth muscle contraction | 12 | 1.77 | 2.31E-02 | 2.13 | AGTR1, ARGHEF12, EDNRA, GUCY1B3, MYL9, MYLK3, NRP1, PLCB4, PRKCH, RAMP2, RAMP3, ROCK1 |
