# Supplementary material for: Systematic Analysis of a Novel Human Renal Glomerulus-Enriched Gene Expression Dataset
Source: PLoS One. 2010 Jul 12;5(7):e11545. doi: 10.1371/journal.pone.0011545 (PMC2902524; doi:10.1371/journal.pone.0011545)
Supplement: Table S7 — Neuron/brain-associated gene list (0.40 MB DOC) [file pone.0011545.s008.doc]

Table S7

| **Entrez Gene** | **Gene Symbol** | **Gene Title** | **Source** |
| --- | --- | --- | --- |
| 40 | ACCN1 | amiloride-sensitive cation channel 1, neuronal | [1] |
| 41 | ACCN2 | amiloride-sensitive cation channel 2, neuronal | [1] |
| 11332 | ACOT7 | acyl-CoA thioesterase 7 | [1] |
| 56 | ACRV1 | acrosomal vesicle protein 1 | [1] |
| 55860 | ACTR10 | actin-related protein 10 homolog (S. cerevisiae) | DDD |
| 27308 | AD7C-NTP | neuronal thread protein AD7c-NTP | [1] |
| 113451 | ADC | arginine decarboxylase | DDD |
| 137872 | ADHFE1 | alcohol dehydrogenase, iron containing, 1 | DDD |
| 151 | ADRA2B | adrenergic, alpha-2B-, receptor | [1] |
| 152 | ADRA2C | adrenergic, alpha-2C-, receptor | [1] |
| 154 | ADRB2 | adrenergic, beta-2-, receptor, surface | [1] |
| 155 | ADRB3 | adrenergic, beta-3-, receptor | [1] |
| 156 | ADRBK1 | adrenergic, beta, receptor kinase 1 | [1] |
| 157 | ADRBK2 | adrenergic, beta, receptor kinase 2 | [1] |
| 375790 | AGRN | agrin | [1] |
| 183 | AGT | angiotensinogen (serpin peptidase inhibitor, clade A, member 8) | DDD |
| 10000 | AKT3 | v-akt murine thymoma viral oncogene homolog 3 (protein kinase B, gamma) | DDD |
| 273 | AMPH | amphiphysin | DDD |
| 324 | APC | adenomatous polyposis coli | DDD |
| 361 | AQP4 | aquaporin 4 | DDD |
| 10552 | ARPC1A | actin related protein 2/3 complex, subunit 1A, 41kDa | DDD |
| 8623 | ASMTL | acetylserotonin O-methyltransferase-like | [1] |
| 460 | ASTN1 | astrotactin 1 | DDD |
| 25814 | ATXN10 | ataxin 10 | [1] |
| 146712 | B3GNTL1 | UDP-GlcNAc:betaGal beta-1,3-N-acetylglucosaminyltransferase-like 1 | DDD |
| 79870 | BAALC | brain and acute leukemia, cytoplasmic | DDD |
| 23621 | BACE1 | beta-site APP-cleaving enzyme 1 | DDD |
| 575 | BAI1 | brain-specific angiogenesis inhibitor 1 | [1] |
| 576 | BAI2 | brain-specific angiogenesis inhibitor 2 | [1] |
| 577 | BAI3 | brain-specific angiogenesis inhibitor 3 | DDD |
| 10409 | BASP1 | brain abundant, membrane attached signal protein 1 | [1] |
| 146227 | BEAN | brain expressed, associated with Nedd4 | [1] |
| 57596 | BEGAIN | brain-enriched guanylate kinase-associated homolog (rat) | [1] |
| 55859 | BEX1 | brain expressed, X-linked 1 | [1] |
| 56271 | BEX4 | brain expressed, X-linked 4 | [1] |
| 51283 | BFAR | bifunctional apoptosis regulator | DDD |
| 51660 | BRP44L | brain protein 44-like | [1] |
| 8927 | BSN | bassoon (presynaptic cytomatrix protein) | [1] |
| 26098 | C10orf137 | chromosome 10 open reading frame 137 | DDD |
| 81563 | C10rf21 | chromosome 1 open reading frame 21 | DDD |
| 10485 | C10rf61 | chromosome 1 open reading frame 61 | DDD |
| 79096 | C11orf49 | chromosome 11 open reading frame 49 | DDD |
| 83932 | C1orf124 | chromosome 1 open reading frame 124 | DDD |
| 126731 | C1orf96 | chromosome 1 open reading frame 96 | DDD |
| 257407 | C2orf72 | chromosome 2 open reading frame 72 | DDD |
| 133686 | C5orf33 | chromosome 5 open reading frame 33 | DDD |
| 387263 | C6orf120 | chromosome 6 open reading frame 120 | DDD |
| 51236 | C8orf30A | chromosome 8 open reading frame 30A | [1] |
| 56934 | CA10 | carbonic anhydrase X | DDD |
| 23705 | CADM1 | cell adhesion molecule 1 | [1] |
| 8618 | CADPS | Ca++-dependent secretion activator | DDD |
| 83698 | CALN1 | calneuron 1 | DDD |
| 818 | CAMK2G | calcium/calmodulin-dependent protein kinase II gamma | DDD |
| 9139 | CBFA2T2 | core-binding factor, runt domain, alpha subunit 2; translocated to, 2 | DDD |
| 4345 | CD200 | CD200 molecule | DDD |
| 1000 | CDH2 | cadherin 2, type 1, N-cadherin (neuronal) | [1] |
| 1006 | CDH8 | cadherin 8, type 2 | DDD |
| 1039 | CDR2 | cerebellar degeneration-related protein 2, 62kDa | [1] |
| 1054 | CEBPG | CCAAT/enhancer binding protein (C/EBP), gamma | DDD |
| 1113 | CHGA | chromogranin A (parathyroid secretory protein 1) | DDD |
| 1124 | CHN2 | chimerin (chimaerin) 2 | DDD |
| 1129 | CHRM2 | cholinergic receptor, muscarinic 2 | [1] |
| 1131 | CHRM3 | cholinergic receptor, muscarinic 3 | [1] |
| 1132 | CHRM4 | cholinergic receptor, muscarinic 4 | [1] |
| 1133 | CHRM5 | cholinergic receptor, muscarinic 5 | [1] |
| 1134 | CHRNA1 | cholinergic receptor, nicotinic, alpha 1 (muscle) | [1] |
| 1135 | CHRNA2 | cholinergic receptor, nicotinic, alpha 2 (neuronal) | [1] |
| 1136 | CHRNA3 | cholinergic receptor, nicotinic, alpha 3 | [1] |
| 1137 | CHRNA4 | cholinergic receptor, nicotinic, alpha 4 | [1] |
| 8973 | CHRNA6 | cholinergic receptor, nicotinic, alpha 6 | [1] |
| 55584 | CHRNA9 | cholinergic receptor, nicotinic, alpha 9 | [1] |
| 1140 | CHRNB1 | cholinergic receptor, nicotinic, beta 1 (muscle) | [1] |
| 1141 | CHRNB2 | cholinergic receptor, nicotinic, beta 2 (neuronal) | [1] |
| 1144 | CHRND | cholinergic receptor, nicotinic, delta | [1] |
| 1146 | CHRNG | cholinergic receptor, nicotinic, gamma | [1] |
| 1152 | CKB | creatine kinase, brain | DDD |
| 23122 | CLASP2 | cytoplasmic linker associated protein 2 | DDD |
| 5010 | CLDN11 | claudin 11 | [1] |
| 56650 | CLDND1 | claudin domain containing 1 | DDD |
| 1201 | CLN3 | ceroid-lipofuscinosis, neuronal 3 | [1] |
| 1203 | CLN5 | ceroid-lipofuscinosis, neuronal 5 | [1] |
| 54982 | CLN6 | ceroid-lipofuscinosis, neuronal 6, late infantile, variant | [1] |
| 2055 | CLN8 | ceroid-lipofuscinosis, neuronal 8 (epilepsy, progressive with mental retardation) | [1] |
| 1191 | CLU | clusterin | DDD |
| 1268 | CNR1 | Homo sapiens, clone IMAGE:5244076, mRNA | [1] |
| 1271 | CNTFR | ciliary neurotrophic factor receptor | [1] |
| 6900 | CNTN2 | contactin 2 (axonal) | [1] |
| 8506 | CNTNAP1 | contactin associated protein 1 | [1] |
| 1312 | COMT | catechol-O-methyltransferase | [1] |
| 10920 | COPS8 | COP9 constitutive photomorphogenic homolog subunit 8 (Arabidopsis) | DDD |
| 1363 | CPE | carboxypeptidase E | DDD |
| 10814 | CPLX2 | complexin 2 | DDD |
| 131034 | CPN4 | copine IV | DDD |
| 9362 | CPNE6 | copine VI (neuronal) | [1] |
| 9419 | CRIPT | cysteine-rich PDZ-binding protein | [1] |
| 1399 | CRKL | v-crk sarcoma virus CT10 oncogene homolog (avian)-like | DDD |
| 27065 | D4S234E | DNA segment on chromosome 4 (unique) 234 expressed sequence | DDD |
| 1621 | DBH | dopamine beta-hydroxylase (dopamine beta-monooxygenase) | [1] |
| 1622 | DBI | diazepam binding inhibitor (GABA receptor modulator, acyl-Coenzyme A binding protein) | [1] |
| 1627 | DBN1 | drebrin 1 | [1] |
| 1741 | DLG3 | discs, large homolog 3 (Drosophila) | [1] |
| 23312 | DMXL2 | Dmx-like 2 | DDD |
| 55567 | DNAH3 | dynein, axonemal, heavy chain 3 | [1] |
| 1770 | DNAH9 | dynein, axonemal, heavy chain 9 | [1] |
| 27019 | DNAI1 | dynein, axonemal, intermediate chain 1 | [1] |
| 80331 | DNAJC5 | DnaJ (Hsp40) homolog, subfamily C, member 5 | DDD |
| 9829 | DNAJC6 | DnaJ (Hsp40) homolog, subfamily C, member 6 | DDD |
| 83544 | DNAL1 | dynein, axonemal, light chain 1 | DDD |
| 10126 | DNAL4 | dynein, axonemal, light chain 4 | [1] |
| 7802 | DNALI1 | dynein, axonemal, light intermediate chain 1 | [1] |
| 85440 | DOCK7 | dedicator of cytokinesis 7 | DDD |
| 57628 | DPP10 | dipeptidyl-peptidase 10 | DDD |
| 1808 | DPYSL2 | dihydropyrimidinase-like 2 | DDD |
| 56896 | DPYSL5 | dihydropyrimidinase-like 5 | DDD |
| 1813 | DRD2 | dopamine receptor D2 | [1] |
| 1816 | DRD5 | dopamine receptor D5 | [1] |
| 1837 | DTNA | dystrobrevin, alpha | DDD |
| 1942 | EFNA1 | ephrin-A1 | [1] |
| 1944 | EFNA3 | ephrin-A3 | [1] |
| 1947 | EFNB1 | ephrin-B1 | [1] |
| 1948 | EFNB2 | ephrin-B2 | [1] |
| 1949 | EFNB3 | ephrin-B3 | [1] |
| 64123 | ELTD1 | EGF, latrophilin and seven transmembrane domain containing 1 | [1] |
| 2026 | ENO2 | enolase 2 (gamma, neuronal) | [1] |
| 57669 | EPB41L5 | erythrocyte membrane protein band 4.1 like 5 | DDD |
| 2039 | EPB49 | erythrocyte membrane protein band 4.9 (dematin) | DDD |
| 2041 | EPHA1 | EPH receptor A1 | [1] |
| 1969 | EPHA2 | EPH receptor A2 | [1] |
| 2043 | EPHA4 | EPH receptor A4 | [1] |
| 2047 | EPHB1 | EPH receptor B1 | [1] |
| 2048 | EPHB2 | EPH receptor B2 | [1] |
| 2049 | EPHB3 | EPH receptor B3 | [1] |
| 2050 | EPHB4 | EPH receptor B4 | [1] |
| 2051 | EPHB6 | EPH receptor B6 | [1] |
| 26059 | ERC2 | ELKS/RAB6-interacting/CAST family member 2 | DDD |
| 79033 | ERI3 | exoribonuclease 3 | [1] |
| 23265 | EXOC7 | exocyst complex component 7 | [1] |
| 404636 | FAM45A | CDNA clone IMAGE:5312680 | [1] |
| 2246 | FGF1 | fibroblast growth factor 1 (acidic) | DDD |
| 23413 | FREQ | frequenin homolog (Drosophila) | DDD |
| 11337 | GABARAP | GABA(A) receptor-associated protein | [1] |
| 23710 | GABARAPL1 | GABA(A) receptor-associated protein like 1 | [1] |
| 11345 | GABARAPL2 | GABA(A) receptor-associated protein-like 2 | [1] |
| 2550 | GABBR1 | gamma-aminobutyric acid (GABA) B receptor, 1 | [1] |
| 2554 | GABRA1 | gamma-aminobutyric acid (GABA) A receptor, alpha 1 | [1] |
| 2561 | GABRB2 | gamma-aminobutyric acid (GABA) A receptor, beta 2 | [1] |
| 2562 | GABRB3 | gamma-aminobutyric acid (GABA) A receptor, beta 3 | DDD |
| 2563 | GABRD | gamma-aminobutyric acid (GABA) A receptor, delta | [1] |
| 55879 | GABRQ | gamma-aminobutyric acid (GABA) receptor, theta | [1] |
| 2570 | GABRR2 | gamma-aminobutyric acid (GABA) receptor, rho 2 | [1] |
| 2571 | GAD1 | glutamate decarboxylase 1 (brain, 67kDa) | DDD |
| 9615 | GDA | guanine deaminase | DDD |
| 78997 | GDAP1L1 | ganglioside-induced differentiation-associated protein 1-like 1 | [1] |
| 54834 | GDAP2 | ganglioside induced differentiation associated protein 2 | [1] |
| 2670 | GFAP | glial fibrillary acidic protein | [1], DDD |
| 2742 | GLRA2 | glycine receptor, alpha 2 | [1] |
| 23127 | GLT25D2 | glycosyltransferase 25 domain containing 2 | DDD |
| 2781 | GNAZ | guanine nucleotide binding protein (G protein), alpha z polypeptide | DDD |
| 2824 | GPM6B | glycoprotein M6B | DDD |
| 2878 | GPX3 | glutathione peroxidase 3 (plasma) | DDD |
| 2890 | GRIA1 | glutamate receptor, ionotropic, AMPA 1 | DDD |
| 2893 | GRIA4 | glutamate receptor, ionotrophic, AMPA 4 | [1] |
| 2895 | GRID2 | glutamate receptor, ionotropic, delta 2 | [1] |
| 2899 | GRIK3 | glutamate receptor, ionotropic, kainate 3 | [1] |
| 2901 | GRIK5 | glutamate receptor, ionotropic, kainate 5 | [1] |
| 2902 | GRIN1 | glutamate receptor, ionotropic, N-methyl D-aspartate 1 | [1] |
| 2903 | GRIN2A | glutamate receptor, ionotropic, N-methyl D-aspartate 2A | [1] |
| 2904 | GRIN2B | glutamate receptor, ionotropic, N-methyl D-aspartate 2B | [1] |
| 2905 | GRIN2C | glutamate receptor, ionotropic, N-methyl D-aspartate 2C | [1] |
| 2907 | GRINA | glutamate receptor, ionotropic, N-methyl D-aspartate-associated protein 1 (glutamate binding) | [1] |
| 81488 | GRINL1A | glutamate receptor, ionotropic, N-methyl D-aspartate-like 1A | [1] |
| 80852 | GRIP2 | glutamate receptor interacting protein 2 | [1] |
| 2911 | GRM1 | glutamate receptor, metabotropic 1 | [1] |
| 2912 | GRM2 | glutamate receptor, metabotropic 2 | [1] |
| 2914 | GRM4 | glutamate receptor, metabotropic 4 | [1] |
| 2915 | GRM5 | glutamate receptor, metabotropic 5 | [1], DDD |
| 2916 | GRM6 | glutamate receptor, metabotropic 6 | [1] |
| 51617 | HMP19 | HMP19 protein | DDD |
| 84525 | HOPX | HOP homeobox | DDD |
| 3208 | HPCA | hippocalcin | [1], DDD |
| 3241 | HPCAL1 | hippocalcin-like 1 | [1] |
| 51440 | HPCAL4 | hippocalcin like 4 | [1], DDD |
| 266722 | HS6ST3 | heparan sulfate 6-O-sulfotransferase 3 | DDD |
| 3350 | HTR1A | 5-hydroxytryptamine (serotonin) receptor 1A | [1] |
| 3351 | HTR1B | 5-hydroxytryptamine (serotonin) receptor 1B | [1] |
| 3352 | HTR1D | 5-hydroxytryptamine (serotonin) receptor 1D | [1] |
| 3354 | HTR1E | 5-hydroxytryptamine (serotonin) receptor 1E | [1] |
| 3359 | HTR3A | 5-hydroxytryptamine (serotonin) receptor 3A | [1] |
| 9177 | HTR3B | 5-hydroxytryptamine (serotonin) receptor 3B | [1] |
| 3360 | HTR4 | 5-hydroxytryptamine (serotonin) receptor 4 | [1] |
| 3361 | HTR5A | 5-hydroxytryptamine (serotonin) receptor 5A | [1] |
| 3362 | HTR6 | 5-hydroxytryptamine (serotonin) receptor 6 | [1] |
| 3363 | HTR7 | 5-hydroxytryptamine (serotonin) receptor 7 (adenylate cyclase-coupled) | [1] |
| 9118 | INA | internexin neuronal intermediate filament protein, alpha | [1] |
| 6453 | ITSN1 | intersectin 1 (SH3 domain protein) | [1] |
| 50618 | ITSN2 | intersectin 2 | [1] |
| 9731 | KIAA0562 | KIAA0562 | [1] |
| 23349 | KIAA1045 | KIAA1045 | DDD |
| 57578 | KIAA1409 | KIAA1409 | DDD |
| 57698 | KIAA1598 | KIAA1598 | DDD |
| 3797 | KIF3C | kinesin family member 3C | DDD |
| 3897 | L1CAM | L1 cell adhesion molecule | [1] |
| 3913 | LAMB2 | laminin, beta 2 (laminin S) | [1] |
| 23185 | LARP4B | La ribonucleoprotein domain family, member 5 | DDD |
| 167410 | LIX1 | Lix1 homolog (chicken) | DDD |
| 64327 | LMBR1 | limb region 1 homolog (mouse) | DDD |
| 100131014 | LOC100131014 | similar to hCG2045213 | DDD |
| 157503 | LOC157503 | hypothetical protein LOC157503 | DDD |
| 254128 | LOC254128 | Hypothetical protein LOC254128, mRNA (cDNA clone IMAGE:5288009) | DDD |
| 283713 | LOC283713 | hypothetical protein LOC283713 | DDD |
| 284244 | LOC284244 | hypothetical protein LOC284244 | DDD |
| 22859 | LPHN1 | latrophilin 1 | [1] |
| 23266 | LPHN2 | latrophilin 2 | [1] |
| 23284 | LPHN3 | latrophilin 3 | [1] |
| 9890 | LPPR4 | plasticity related gene 1 | DDD |
| 10446 | LRRN2 | leucine rich repeat neuronal 2 | [1] |
| 54674 | LRRN3 | leucine rich repeat neuronal 3 | [1], DDD |
| 4045 | LSAMP | Limbic system-associated membrane protein LAMP | [1] |
| 66004 | LYNX1 | Ly6/neurotoxin 1 | DDD |
| 4131 | MAP1B | microtubule-associated protein 1B | DDD |
| 4133 | MAP2 | microtubule-associated protein 2 | [1] |
| 4293 | MAP3K9 | mitogen-activated protein kinase kinase kinase 9 | DDD |
| 4137 | MAPT | microtubule-associated protein tau | [1] |
| 27430 | MAT2B | methionine adenosyltransferase II, beta | DDD |
| 4155 | MBP | myelin basic protein | DDD |
| 55384 | MEG3 | maternally expressed 3 (non-protein coding) | DDD |
| 2315 | MLANA | melan-A | DDD |
| 23209 | MLC1 | megalencephalic leukoencephalopathy with subcortical cysts 1 | [1] |
| 4325 | MMP16 | matrix metallopeptidase 16 (membrane-inserted) | DDD |
| 10893 | MMP24 | matrix metallopeptidase 24 (membrane-inserted) | DDD |
| 4336 | MOBP | myelin-associated oligodendrocyte basic protein | DDD |
| 4628 | MYH10 | myosin, heavy chain 10, non-muscle | DDD |
| 8775 | NAPA | N-ethylmaleimide-sensitive factor attachment protein, alpha | [1] |
| 79664 | NARG2 | NMDA receptor regulated 2 | [1] |
| 89797 | NAV2 | neuron navigator 2 | [1] |
| 89795 | NAV3 | neuron navigator 3 | [1] |
| 26960 | NBEA | neurobeachin | [1] |
| 4684 | NCAM1 | neural cell adhesion molecule 1 | [1] |
| 4747 | NEFL | neurofilament, light polypeptide | DDD |
| 4741 | NEFM | neurofilament, medium polypeptide | [1] |
| 4761 | NEUROD2 | neurogenic differentiation 2 | [1] |
| 58158 | NEUROD4 | neurogenic differentiation 4 | [1] |
| 63974 | NEUROD6 | neurogenic differentiation 6 | [1] |
| 4762 | NEUROG1 | neurogenin 1 | [1] |
| 23114 | NFASC | neurofascin homolog (chicken) | [1], DDD |
| 4803 | NGF | nerve growth factor (beta polypeptide) | [1] |
| 4804 | NGFR | nerve growth factor receptor (TNFR superfamily, member 16) | [1] |
| 54413 | NLGN3 | neuroligin 3 | [1] |
| 57502 | NLGN4X | neuroligin 4, X-linked | [1] |
| 4828 | NMB | neuromedin B | [1] |
| 4826 | NNAT | neuronatin | [1] |
| 4861 | NPAS1 | neuronal PAS domain protein 1 | [1] |
| 4862 | NPAS2 | neuronal PAS domain protein 2 | [1] |
| 64067 | NPAS3 | neuronal PAS domain protein 3 | [1] |
| 56654 | NPDC1 | neural proliferation, differentiation and control, 1 | [1] |
| 4884 | NPTX1 | neuronal pentraxin I | [1] |
| 4885 | NPTX2 | neuronal pentraxin II | [1] |
| 23467 | NPTXR | neuronal pentraxin receptor | [1] |
| 4852 | NPY | neuropeptide Y | [1] |
| 4886 | NPY1R | neuropeptide Y receptor Y1 | [1] |
| 4887 | NPY2R | neuropeptide Y receptor Y2 | [1] |
| 4888 | NPY6R | neuropeptide Y receptor Y6 (pseudogene) | [1] |
| 10002 | NR2E3 | nuclear receptor subfamily 2, group E, member 3 | [1] |
| 4897 | NRCAM | neuronal cell adhesion molecule | [1], DDD |
| 3084 | NRG1 | neuregulin 1 | [1] |
| 4900 | NRGN | neurogranin (protein kinase C substrate, RC3) | [1] |
| 51299 | NRN1 | neuritin 1 | [1] |
| 9378 | NRXN1 | neurexin 1 | [1] |
| 9369 | NRXN3 | neurexin 3 | [1] |
| 64943 | NT5DC2 | 5'-nucleotidase domain containing 2 (NT5DC2), transcript variant 1, mRNA | DDD |
| 4908 | NTF3 | neurotrophin 3 | [1] |
| 50863 | NTM | neurotrimin | DDD |
| 4915 | NTRK2 | neurotrophic tyrosine kinase, receptor, type 2 | [1], DDD |
| 4916 | NTRK3 | neurotrophic tyrosine kinase, receptor, type 3 | [1] |
| 4923 | NTSR1 | neurotensin receptor 1 (high affinity) | [1] |
| 23620 | NTSR2 | neurotensin receptor 2 | [1] |
| 11248 | NXPH3 | neurexophilin 3 | [1] |
| 11247 | NXPH4 | neurexophilin 4 | [1] |
| 10439 | OLFM1 | olfactomedin 1 | DDD |
| 4974 | OMG | oligodendrocyte myelin glycoprotein | [1] |
| 93377 | OPALIN | oligodendrocytic myelin paranodal and inner loop protein | DDD |
| 4978 | OPCML | opioid binding protein/cell adhesion molecule-like | [1] |
| 4983 | OPHN1 | oligophrenin 1 | [1] |
| 4985 | OPRD1 | opioid receptor, delta 1 | [1] |
| 4987 | OPRL1 | opiate receptor-like 1 | [1] |
| 4988 | OPRM1 | opioid receptor, mu 1 | [1] |
| 5023 | P2RX1 | purinergic receptor P2X, ligand-gated ion channel, 1 | [1] |
| 22953 | P2RX2 | purinergic receptor P2X, ligand-gated ion channel, 2 | [1] |
| 5025 | P2RX4 | purinergic receptor P2X, ligand-gated ion channel, 4 | [1] |
| 5026 | P2RX5 | purinergic receptor P2X, ligand-gated ion channel, 5 | [1] |
| 79957 | PAQR6 | progestin and adipoQ receptor family member VI | DDD |
| 117583 | PARD3B | par-3 partitioning defective 3 homolog B (C. elegans) | DDD |
| 5087 | PBX1 | pre-B-cell leukemia homeobox 1 | DDD |
| 57526 | PCDH19 | protocadherin 19 | DDD |
| 56142 | PCDHA6 | protocadherin alpha 6 | DDD |
| 27445 | PCLO | piccolo (presynaptic cytomatrix protein) | [1] |
| 5121 | PCP4 | Purkinje cell protein 4 | [1] |
| 8682 | PEA15 | phosphoprotein enriched in astrocytes 15 | [1] |
| 23089 | PEG10 | paternally expressed 10 | DDD |
| 5217 | PFN2 | profilin 2 | DDD |
| 51317 | PHF21A | PHD finger protein 21A | DDD |
| 9796 | PHYHIP | phytanoyl-CoA 2-hydroxylase interacting protein | DDD |
| 84457 | PHYHIPL | phytanoyl-CoA 2-hydroxylase interacting protein-like | DDD |
| 5295 | PIK3R1 | phosphoinositide-3-kinase, regulatory subunit 1 (alpha) | DDD |
| 8502 | PKP4 | plakophilin 4 | DDD |
| 5354 | PLP1 | proteolipid protein 1 | DDD |
| 5521 | PPP2R2B | protein phosphatase 2 (formerly 2A), regulatory subunit B, beta isoform | DDD |
| 5526 | PPP2R5B | protein phosphatase 2, regulatory subunit B', beta isoform | DDD |
| 5579 | PRKCB | protein kinase C, beta | DDD |
| 5621 | PRNP | prion protein | [1] |
| 84249 | PSD2 | pleckstrin and Sec7 domain containing 2 | DDD |
| 5789 | PTPRD | protein tyrosine phosphatase, receptor type, D | DDD |
| 5864 | RAB3A | RAB3A, member RAS oncogene family | [1], DDD |
| 9545 | RAB3D | RAB3D, member RAS oncogene family | [1] |
| 22930 | RAB3GAP1 | RAB3 GTPase activating protein subunit 1 (catalytic) | [1] |
| 25782 | RAB3GAP2 | RAB3 GTPase activating protein subunit 2 (non-catalytic) | [1] |
| 5866 | RAB3IL1 | RAB3A interacting protein (rabin3)-like 1 | [1] |
| 23543 | RBM9 | RNA binding motif protein 9 | DDD |
| 5992 | RFX4 | regulatory factor X, 4 (influences HLA class II expression) | DDD |
| 23504 | RIMBP2 | RIMS binding protein 2 | [1] |
| 284716 | RIMKLA | ribosomal modification protein rimK-like family member A | DDD |
| 22999 | RIMS1 | regulating synaptic membrane exocytosis 1 | [1] |
| 9699 | RIMS2 | regulating synaptic membrane exocytosis 2 | [1] |
| 9783 | RIMS3 | regulating synaptic membrane exocytosis 3 | [1] |
| 6091 | ROBO1 | roundabout, axon guidance receptor, homolog 1 (Drosophila) | [1] |
| 64221 | ROBO3 | roundabout, axon guidance receptor, homolog 3 (Drosophila) | [1] |
| 22895 | RPH3A | rabphilin 3A homolog (mouse) | [1] |
| 9501 | RPH3AL | rabphilin 3A-like (without C2 domains) | [1] |
| 6252 | RTN1 | reticulon 1 | DDD |
| 10900 | RUNDC3A | RUN domain containing 3A | DDD |
| 6309 | SC5DL | sterol-C5-desaturase (ERG3 delta-5-desaturase homolog, S. cerevisiae)-like | DDD |
| 57556 | SEMA6A | MRNA, clone: FBR89, from chromosome 5q21-q22 | DDD |
| 5413 | 5-Sep | #NV | DDD |
| 23544 | SEZ6L | seizure related 6 homolog (mouse)-like | DDD |
| 26470 | SEZ6L2 | CDNA FLJ90517 fis, clone NT2RP3004552, highly similar to Type I transmembrane receptor | DDD |
| 344558 | SH3RF3 | SH3 domain containing ring finger 3 | DDD |
| 10280 | SIGMAR1 | sigma non-opioid intracellular receptor 1 | [1] |
| 57030 | SLC17A7 | solute carrier family 17 (sodium-dependent inorganic phosphate cotransporter), member 7 | [1] |
| 6570 | SLC18A1 | solute carrier family 18 (vesicular monoamine), member 1 | [1] |
| 6571 | SLC18A2 | solute carrier family 18 (vesicular monoamine), member 2 | [1] |
| 6572 | SLC18A3 | solute carrier family 18 (vesicular acetylcholine), member 3 | [1] |
| 6505 | SLC1A1 | solute carrier family 1 (neuronal/epithelial high affinity glutamate transporter, system Xag), member 1 | [1] |
| 6506 | SLC1A2 | solute carrier family 1 (glial high affinity glutamate transporter), member 2 | [1], DDD |
| 6507 | SLC1A3 | solute carrier family 1 (glial high affinity glutamate transporter), member 3 | [1] |
| 6511 | SLC1A6 | solute carrier family 1 (high affinity aspartate/glutamate transporter), member 6 | [1] |
| 60482 | SLC5A7 | solute carrier family 5 (choline transporter), member 7 | [1] |
| 6538 | SLC6A11 | solute carrier family 6 (neurotransmitter transporter, GABA), member 11 | [1] |
| 6539 | SLC6A12 | solute carrier family 6 (neurotransmitter transporter, betaine/GABA), member 12 | [1] |
| 6540 | SLC6A13 | solute carrier family 6 (neurotransmitter transporter, GABA), member 13 | [1] |
| 28968 | SLC6A16 | CDNA clone IMAGE:5268379 | [1] |
| 6530 | SLC6A2 | solute carrier family 6 (neurotransmitter transporter, noradrenalin), member 2 | [1] |
| 6531 | SLC6A3 | solute carrier family 6 (neurotransmitter transporter, dopamine), member 3 | [1] |
| 6532 | SLC6A4 | solute carrier family 6 (neurotransmitter transporter, serotonin), member 4 | [1] |
| 9152 | SLC6A5 | solute carrier family 6 (neurotransmitter transporter, glycine), member 5 | [1] |
| 6533 | SLC6A6 | solute carrier family 6 (neurotransmitter transporter, taurine), member 6 | [1] |
| 6534 | SLC6A7 | solute carrier family 6 (neurotransmitter transporter, L-proline), member 7 | [1] |
| 6535 | SLC6A8 | solute carrier family 6 (neurotransmitter transporter, creatine), member 8 | [1] |
| 6536 | SLC6A9 | solute carrier family 6 (neurotransmitter transporter, glycine), member 9 | [1] |
| 6616 | SNAP25 | HUMSNAP25B(F) | [1], DDD |
| 9892 | SNAP91 | synaptosomal-associated protein, 91kDa homolog (mouse) | DDD |
| 6622 | SNCA | synuclein, alpha (non A4 component of amyloid precursor) | [1] |
| 9627 | SNCAIP | synuclein, alpha interacting protein | [1] |
| 6620 | SNCB | synuclein, beta | [1] |
| 9751 | SNPH | syntaphilin | [1] |
| 55084 | SOBP | sine oculis binding protein homolog (Drosophila) | DDD |
| 6733 | SRPK2 | SFRS protein kinase 2 | DDD |
| 117178 | SSX2IP | synovial sarcoma, X breakpoint 2 interacting protein | DDD |
| 81849 | ST6GALNAC5 | Alpha 2,6-sialyltransferase (ST6GALNAC V gene) | DDD |
| 3925 | STMN1 | stathmin 1/oncoprotein 18 | DDD |
| 11075 | STMN2 | stathmin-like 2 | DDD |
| 6804 | STX1A | syntaxin 1A (brain) | [1] |
| 6812 | STXBP1 | syntaxin binding protein 1 | DDD |
| 6813 | STXBP2 | syntaxin binding protein 2 | [1] |
| 6814 | STXBP3 | syntaxin binding protein 3 | [1] |
| 9900 | SV2A | synaptic vesicle glycoprotein 2A | [1], DDD |
| 9899 | SV2B | synaptic vesicle glycoprotein 2B | [1] |
| 6853 | SYN1 | synapsin I | [1] |
| 6854 | SYN2 | synapsin II | [1] |
| 9145 | SYNGR1 | synaptogyrin 1 | [1] |
| 6855 | SYP | synaptophysin | [1] |
| 6856 | SYPL1 | synaptophysin-like 1 | [1] |
| 6857 | SYT1 | synaptotagmin I | [1], DDD |
| 23208 | SYT11 | synaptotagmin XI | [1], DDD |
| 91683 | SYT12 | Synaptotagmin XII, mRNA (cDNA clone MGC:46295 IMAGE:5770140) | [1] |
| 57586 | SYT13 | synaptotagmin XIII | [1] |
| 6860 | SYT4 | synaptotagmin IV | DDD |
| 9066 | SYT7 | synaptotagmin VII | DDD |
| 29114 | TAGLN3 | transgelin 3 | [1] |
| 23329 | TBC1D30 | TBC1 domain family, member 30 | DDD |
| 7070 | THY1 | Thy-1 cell surface antigen | DDD |
| 53346 | TM6SF1 | transmembrane 6 superfamily member 1 | DDD |
| 441151 | TMEM151B | transmembrane protein 151B | DDD |
| 25907 | TMEM158 | transmembrane protein 158 | [1] |
| 80723 | TMEM22 | transmembrane protein 22 | DDD |
| 25789 | TMEM59L | transmembrane protein 59-like | DDD |
| 1200 | TPP1 | tripeptidyl peptidase I | [1] |
| 51673 | TPPP3 | tubulin polymerization-promoting protein family member 3 | [1] |
| 114088 | TRIM9 | tripartite motif-containing 9 | DDD |
| 95681 | TSGA14 | CDNA FLJ54115 complete cds, moderately similar to Centrosomal protein of 41 kDa | DDD |
| 23270 | TSPYL4 | TSPY-like 4 | DDD |
| 85453 | TSPYL5 | TSPY-like 5 | DDD |
| 23508 | TTC9 | tetratricopeptide repeat domain 9 | DDD |
| 7846 | TUBA1A | tubulin, alpha 1a | [1], DDD |
| 347733 | TUBB2B | tubulin, beta 2B | DDD |
| 7345 | UCHL1 | Protein gene product (PGP) 9.5 | [1] |
| 6843 | VAMP1 | vesicle-associated membrane protein 1 (synaptobrevin 1) | [1] |
| 6844 | VAMP2 | vesicle-associated membrane protein 2 (synaptobrevin 2) | [1] |
| 10493 | VAT1 | vesicle amine transport protein 1 homolog (T. californica) | [1] |
| 7425 | VGF | VGF nerve growth factor inducible | [1] |
| 7447 | VSNL1 | visinin-like 1 | DDD |
| 55884 | WSB2 | WD repeat and SOCS box-containing 2 | DDD |
| 7504 | XK | X-linked Kx blood group (McLeod syndrome) | DDD |
| 9213 | XPR1 | xenotropic and polytropic retrovirus receptor | DDD |
| 10771 | ZMYND11 | zinc finger, MYND domain containing 11 | DDD |

1. Rastaldi MP, Armelloni S, Berra S, Calvaresi N, Corbelli A, et al. (2006) Glomerular podocytes contain neuron-like functional synaptic vesicles. Faseb J 20: 976-978.
