# Supplementary material for: Systematic Analysis of a Novel Human Renal Glomerulus-Enriched Gene Expression Dataset
Source: PLoS One. 2010 Jul 12;5(7):e11545. doi: 10.1371/journal.pone.0011545 (PMC2902524; doi:10.1371/journal.pone.0011545)
Supplement: Table S8 — Neuron/brain-associated genes present in the REGGED (0.07 MB DOC) [file pone.0011545.s009.doc]

Table S8

| **Probeset ID** | **Entrez Gene** | **Gene Symbol** | **Mean G – Mean T** | **Gene Title** | **Gene Ontology Biological Process (GO IDs)** | **Pathway** |
| --- | --- | --- | --- | --- | --- | --- |
| 217419_x_at | 375790 | AGRN | 2.82 | agrin | 0007009, 0007165, 0007213, 0007268, 0007528, 0008582, 0043113, 0045162, 0045213, 0045944, 0050808 |  |
| 212285_s_at | 375790 | AGRN | 2.68 | agrin | 0007009, 0007165, 0007213, 0007268, 0007528, 0008582, 0043113, 0045162, 0045213, 0045944, 0050808 |  |
| 212609_s_at | 10000 | AKT3 | 3.09 | Clones 23920 and 23921 mRNA sequence | 0006468, 0007165 | Integrin-mediated cell adhesion, S1P Signaling |
| 203525_s_at | 324 | APC | 2.66 | adenomatous polyposis coli | 0006461, 0006974, 0007026, 0007049, 0007050, 0007094, 0007155, 0008285, 0009798, 0009952, 0009953, 0009954, 0016055, 0030178, 0030334, 0030335, 0031274 , 0035019, 0045736, 0045786, 0050680, 0051726, 0051988, 0060070 | Wnt signaling |
| 217904_s_at | 23621 | BACE1 | 2.05 | beta-site APP-cleaving enzyme 1 | 0006508, 0006509, 0050435, 0050435 |  |
| 218332_at | 55859 | BEX1 | 1.96 | brain expressed, X-linked 1 | 0007275, 0007399, 0030154 |  |
| 215440_s_at | 56271 | BEX4 | 2.27 | brain expressed, X-linked 4 |  |  |
| 221272_s_at | 81563 | C1orf21 | 2.19 | chromosome 1 open reading frame 21 |  |  |
| 220889_s_at | 56934 | CA10 | 4.15 | carbonic anhydrase X | 0006730, 0007420 |  |
| 209583_s_at | 4345 | CD200 | 3.48 | CD200 molecule |  |  |
| 209582_s_at | 4345 | CD200 | 2.11 | CD200 molecule |  |  |
| 202806_at | 1627 | DBN1 | 2.51 | drebrin 1 | 0007015, 0007275, 0007399, 0030154, 0048168, 0050773 |  |
| 200762_at | 1808 | DPYSL2 | 1.88 | dihydropyrimidinase-like 2 | 0006139, 0007165, 0007275, 0007399, 0030154 |  |
| 202668_at | 1948 | EFNB2 | 3.64 | ephrin-B2 | 0001945, 0007267, 0007275, 0007399, 0009653, 0009887, 0030154, 0044419 |  |
| 205117_at | 2246 | FGF1 | 3.61 | fibroblast growth factor 1 (acidic) | 0001525, 0001759, 0007165, 0007275, 0008283, 0008543, 0009653, 0030154, 0030324, 0050679 |  |
| 208240_s_at | 2246 | FGF1 | 2.89 | fibroblast growth factor 1 (acidic) | 0001525, 0001759, 0007165, 0007275, 0008283, 0008543, 0009653, 0030154, 0030324, 0050679 |  |
| 209883_at | 23127 | GLT25D2 | 2.27 | glycosyltransferase 25 domain containing 2 | 9103 |  |
| 216264_s_at | 3913 | LAMB2 | 2.63 | laminin, beta 2 (laminin S) | 0007155, 0048677 | Inflammatory Response Pathway |
| 212233_at | 4131 | MAP1B | 2.61 | microtubule-associated protein 1B | 0001578, 0007026, 0016358 |  |
| 221207_s_at | 26960 | NBEA | 1.85 | neurobeachin |  |  |
| 213438_at | 23114 | NFASC | 4.49 | neurofascin homolog (chicken) | 7155 |  |
| 218086_at | 56654 | NPDC1 | 1.98 | neural proliferation, differentiation and control, 1 |  |  |
| 218625_at | 51299 | NRN1 | 2.34 | neuritin 1 |  |  |
| 221796_at | 4915 | NTRK2 | 3.45 | neurotrophic tyrosine kinase, receptor, type 2 | 0006468 , 0007169, 0007190, 0007275, 0007399, 0030154, 0031547, 0046777, 0046928, 0050773, 0051968 |  |
| 213960_at | 4916 | NTRK3 | 1.86 | neurotrophic tyrosine kinase, receptor, type 3 | 0006468, 0007169, 0007169, 0007275, 0007399, 0030154 |  |
| 200788_s_at | 8682 | PEA15 | 3.11 | phosphoprotein enriched in astrocytes 15 | 0006810, 0006915, 0006916, 0008643, 0042981, 0042981, 0046325 | Calcium regulation in cardiac_cells |
| 200787_s_at | 8682 | PEA15 | 2.30 | phosphoprotein enriched in astrocytes 15 | 0006810, 0006915, 0006916, 0008643, 0042981, 0042981, 0046325 | Calcium regulation in cardiac_cells |
| 205325_at | 9796 | PHYHIP | 2.81 | phytanoyl-CoA 2-hydroxylase interacting protein |  |  |
| 213849_s_at | 5521 | PPP2R2B | 2.74 | protein phosphatase 2 (formerly 2A), regulatory subunit B, beta isoform | 0007165, 0007286, 0008219 | Glycogen Metabolism |
| 214043_at | 5789 | PTPRD | 1.84 | protein tyrosine phosphatase, receptor type, D | 0006470, 0006796, 0007185, 0016311 |  |
| 216215_s_at | 23543 | RBM9 | 2.17 | RNA binding motif protein 9 | 0006397, 0008380, 0016070, 0016481, 0030520, 0042127 |  |
| 213194_at | 6091 | ROBO1 | 3.70 | roundabout, axon guidance receptor, homolog 1 (Drosophila) | 0006935, 0007155, 0007156, 0007275, 0007399, 0007399, 0007411, 0030154, 0050772 |  |
| 221614_s_at | 9501 | RPH3AL | 1.84 | rabphilin 3A-like (without C2 domains) | 0006886, 0006887 |  |
| 202508_s_at | 6616 | SNAP25 | 1.97 | synaptosomal-associated protein, 25kDa | 0001504, 0007268, 0007269, 0016081, 0050796 |  |
| 204466_s_at | 6622 | SNCA | 3.57 | synuclein, alpha (non A4 component of amyloid precursor) | 0001956 , 0001963 , 0006644 , 0006916 , 0007417, 0014059, 0032769, 0040012, 0042416, 0042417, 0042493, 0046928, 0048169, 0048489 |  |
| 204467_s_at | 6622 | SNCA | 2.96 | synuclein, alpha (non A4 component of amyloid precursor) | 0001956 , 0001963 , 0006644 , 0006916 , 0007417, 0014059, 0032769, 0040012, 0042416, 0042417, 0042493, 0046928, 0048169, 0048489 |  |
| 202260_s_at | 6812 | STXBP1 | 2.02 | syntaxin binding protein 1 | 0006810, 0006904, 0015031, 0016192 |  |
| 218876_at | 51673 | TPPP3 | 5.35 | tubulin polymerization-promoting protein family member 3 |  |  |
| 212928_at | 23270 | TSPYL4 | 1.90 | TSPY-like 4 | 6334 |  |
| 213122_at | 85453 | TSPYL5 | 1.99 | TSPY-like 5 | 6334 |  |
| 209118_s_at | 7846 | TUBA1A | 2.22 | tubulin, alpha 1a | 0007017, 0007018, 0051258 |  |
| 213326_at | 6843 | VAMP1 | 1.87 | vesicle-associated membrane protein 1 (synaptobrevin 1) | 16192 |  |
| 201556_s_at | 6844 | VAMP2 | 2.47 | vesicle-associated membrane protein 2 (synaptobrevin 2) | 0006944, 0016079, 0016192, 0017156, 0017157 |  |
