# Supplementary material for: Systematic Analysis of a Novel Human Renal Glomerulus-Enriched Gene Expression Dataset
Source: PLoS One. 2010 Jul 12;5(7):e11545. doi: 10.1371/journal.pone.0011545 (PMC2902524; doi:10.1371/journal.pone.0011545)
Supplement: Table S9 — Smooth muscle cell associated gene list. Genes marked in bold font are present in REGGED. (0.10 MB DOC) [file pone.0011545.s010.doc]

Table S9

| **Entrez Gene** | **Gene Symbol** | **Gene Title** | **Source** |
| --- | --- | --- | --- |
| 25890 | ABI3BP | ABI family, member 3 (NESH) binding protein (ABI3BP) | DDD |
| 59 | ACTA2 | Actin, alpha 2, smooth muscle, aorta (ACTA2) | DDD |
| 60 | ACTB | Actin, beta (ACTB) | DDD |
| 102 | ADAM10 | ADAM metallopeptidase domain 10 (ADAM10) | DDD |
| 54443 | ANLN | Anillin, actin binding protein (ANLN) | DDD |
| 308 | ANXA5 | Annexin A5 (ANXA5) | DDD |
| 10092 | ARPC5 | Actin related protein 2/3 complex, subunit 5, 16kDa (ARPC5) | DDD |
| 468 | ATF4 | Activating transcription factor 4 (tax-responsive enhancer element B67) (ATF4) | DDD |
| 22926 | ATF6 | Activating transcription factor 6 (ATF6) | DDD |
| 8702 | B4GALT4 | UDP-Gal:betaGlcNAc beta 1,4- galactosyltransferase, polypeptide 4 (B4GALT4) | DDD |
| 9689 | BZW1 | Basic leucine zipper and W2 domains 1 (BZW1) | DDD |
| 81688 | C6orf62 | Chromosome 6 open reading frame 62 (C6orf62) | DDD |
| 801 | CALM1 | Calmodulin 1 (phosphorylase kinase, delta) (CALM1) | DDD |
| 813 | CALU | Calumenin (CALU) | DDD |
| 10574 | CCT7 | Chaperonin containing TCP1, subunit 7 (eta) (CCT7) | DDD |
| 960 | CD44 | CD44 molecule (Indian blood group) (CD44) | DDD |
| 966 | CD59 | CD59 molecule, complement regulatory protein (CD59) | DDD |
| 1000 | CDH2 | Cadherin 2, type 1, N-cadherin (neuronal) (CDH2) | DDD |
| 25932 | CLIC4 | Chloride intracellular channel 4 (CLIC4) | DDD |
| 23603 | CORO1C | Coronin, actin binding protein, 1C (CORO1C) | DDD |
| **51232** | **CRIM1** | **Cysteine rich transmembrane BMP regulator 1 (chordin-like) (CRIM1)** | **DDD** |
| 2919 | CXCL1 | Chemokine (C-X-C motif) ligand 1 (melanoma growth stimulating activity, alpha) (CXCL1) | DDD |
| 6372 | CXCL6 | Chemokine (C-X-C motif) ligand 6 (granulocyte chemotactic protein 2) (CXCL6) | DDD |
| 1615 | DARS | Aspartyl-tRNA synthetase (DARS) | DDD |
| 9249 | DHRS3 | Dehydrogenase/reductase (SDR family) member 3 (DHRS3) | DDD |
| 55740 | ENAH | Enabled homolog (Drosophila) (ENAH) | DDD |
| 2023 | ENO1 | Enolase 1, (alpha) (ENO1) | DDD |
| 122786 | FRMD6 | FERM domain containing 6 (FRMD6) | DDD |
| 55783 | FTSJD1 | FtsJ methyltransferase domain containing 1 (FTSJD1) | DDD |
| 2739 | GLO1 | Glyoxalase I (GLO1) | DDD |
| 2876 | GPX1 | Glutathione peroxidase 1 (GPX1) | DDD |
| 26585 | GREM1 | Gremlin 1, cysteine knot superfamily, homolog (Xenopus laevis) (GREM1) | DDD |
| **51454** | **GULP1** | **GULP, engulfment adaptor PTB domain containing 1 (GULP1)** | **DDD** |
| 3069 | HDLBP | High density lipoprotein binding protein (HDLBP) | DDD |
| 3074 | HEXB | Hexosaminidase B (beta polypeptide) (HEXB) | DDD |
| 3091 | HIF1A | Hypoxia inducible factor 1, alpha subunit (basic helix-loop-helix transcription factor) (HIF1A) | DDD |
| 3320 | HSP90AA1 | Heat shock protein 90kDa alpha (cytosolic), class A member 1 (HSP90AA1) | DDD |
| 3552 | IL1A | Interleukin 1, alpha (IL1A) | DDD |
| 3553 | IL1B | Interleukin 1, beta (IL1B) | DDD |
| 3576 | IL8 | Interleukin 8 (IL8) | DDD |
| 3624 | INHBA | Inhibin, beta A (INHBA) | DDD |
| 440917 | LOC440917 | Similar to 14-3-3 protein epsilon (14-3-3E) (Mitochondrial import stimulation factor L subunit) (MSF L) (LOC440917) | DDD |
| **4015** | **LOX** | **Lysyl oxidase (LOX)** | **DDD** |
| 8076 | MFAP5 | Microfibrillar associated protein 5 (MFAP5) | DDD |
| 27249 | MMADHC | Methylmalonic aciduria (cobalamin deficiency) cblD type, with homocystinuria (MMADHC) | DDD |
| 4312 | MMP1 | Matrix metallopeptidase 1 (interstitial collagenase) (MMP1) | DDD |
| 22916 | NCBP2 | Nuclear cap binding protein subunit 2, 20kDa (NCBP2) | DDD |
| 4731 | NDUFV3 | NADH dehydrogenase (ubiquinone) flavoprotein 3, 10kDa (NDUFV3) | DDD |
| 8204 | NRIP1 | Nuclear receptor interacting protein 1 (NRIP1) | DDD |
| 11163 | NUDT4 | Nudix (nucleoside diphosphate linked moiety X)-type motif 4 (NUDT4) | DDD |
| 26986 | PABPC1 | Poly(A) binding protein, cytoplasmic 1 (PABPC1) | DDD |
| 5069 | PAPPA | Pregnancy-associated plasma protein A, pappalysin 1 (PAPPA) | DDD |
| 5283 | PIGH | Phosphatidylinositol glycan anchor biosynthesis, class H (PIGH) | DDD |
| **10631** | **POSTN** | **Periostin, osteoblast specific factor (POSTN)** | **DDD** |
| 5702 | PSMC3 | Proteasome (prosome, macropain) 26S subunit, ATPase, 3 (PSMC3) | DDD |
| 5743 | PTGS2 | Prostaglandin-endoperoxide synthase 2 (prostaglandin G/H synthase and cyclooxygenase) (PTGS2) | DDD |
| **11031** | **RAB31** | **RAB31, member RAS oncogene family (RAB31)** | **DDD** |
| 5917 | RARS | Arginyl-tRNA synthetase (RARS) | DDD |
| 5999 | RGS4 | Regulator of G-protein signaling 4 (RGS4) | DDD |
| 22836 | RHOBTB3 | Rho-related BTB domain containing 3 (RHOBTB3) | DDD |
| 6154 | RPL26 | Ribosomal protein L26 (RPL26) | DDD |
| 6184 | RPN1 | Ribophorin I (RPN1) | DDD |
| 5054 | SERPINE1 | Serpin peptidase inhibitor, clade E (nexin, plasminogen activator inhibitor type 1), member 1 (SERPINE1) | DDD |
| 871 | SERPINH1 | Serpin peptidase inhibitor, clade H (heat shock protein 47), member 1, (collagen binding protein 1) (SERPINH1) | DDD |
| 6451 | SH3BGRL | SH3 domain binding glutamic acid-rich protein like (SH3BGRL) | DDD |
| 81539 | SLC38A1 | Solute carrier family 38, member 1 (SLC38A1) | DDD |
| 8405 | SPOP | Speckle-type POZ protein (SPOP) | DDD |
| **5552** | **SRGN** | **Serglycin (SRGN)** | **DDD** |
| 7980 | TFPI2 | Tissue factor pathway inhibitor 2 (TFPI2) | DDD |
| 7045 | TGFBI | Transforming growth factor, beta-induced, 68kDa (TGFBI) | DDD |
| 7057 | THBS1 | Thrombospondin 1 (THBS1) | DDD |
| **4071** | **TM4SF1** | **Transmembrane 4 L six family member 1 (TM4SF1)** | **DDD** |
| 80008 | TMEM156 | Transmembrane protein 156 (TMEM156) | DDD |
| 55754 | TMEM30A | Transmembrane protein 30A (TMEM30A) | DDD |
| 7114 | TMSB4X | Thymosin beta 4, X-linked (TMSB4X) | DDD |
| 7168 | TPM1 | Tropomyosin 1 (alpha) (TPM1) | DDD |
| 84617 | TUBB6 | Tubulin, beta 6 (TUBB6) | DDD |
| 7296 | TXNRD1 | Thioredoxin reductase 1 (TXNRD1) | DDD |
| 10054 | UBA2 | Ubiquitin-like modifier activating enzyme 2 (UBA2) | DDD |
| 7358 | UGDH | UDP-glucose dehydrogenase (UGDH) | DDD |
| **10600** | **USP16** | **Ubiquitin specific peptidase 16 (USP16)** | **DDD** |
| 9341 | VAMP3 | Vesicle-associated membrane protein 3 (cellubrevin) (VAMP3) | DDD |
| **7431** | **VIM** | **Vimentin (VIM)** | **DDD** |
| 2547 | XRCC6 | X-ray repair complementing defective repair in Chinese hamster cells 6 (XRCC6) | DDD |
| 81555 | YIPF5 | Yip1 domain family, member 5 (YIPF5) | DDD |
| 7531 | YWHAE | Tyrosine 3-monooxygenase/tryptophan 5-monooxygenase activation protein, epsilon polypeptide (YWHAE) | DDD |
| 10168 | ZNF197 | Zinc finger protein 197 (ZNF197) | DDD |
| 7756 | ZNF207 | Zinc finger protein 207 (ZNF207) | DDD |
| 221302 | ZUFSP | Zinc finger with UFM1-specific peptidase domain (ZUFSP) | DDD |
