# Supplementary material for: Systematic Analysis of a Novel Human Renal Glomerulus-Enriched Gene Expression Dataset
Source: PLoS One. 2010 Jul 12;5(7):e11545. doi: 10.1371/journal.pone.0011545 (PMC2902524; doi:10.1371/journal.pone.0011545)
Supplement: Table S10 — Muscle- and heart associated gene list. Genes marked in bold font are present in REGGED. (0.16 MB DOC) [file pone.0011545.s011.doc]

Table S10

| **Entrez Gene** | **Gene Symbol** | **Gene Title** | **Source** |
| --- | --- | --- | --- |
| 58 | ACTA1 | Actin, alpha 1, skeletal muscle (ACTA1) | DDD |
| 70 | ACTC1 | Actin, alpha, cardiac muscle 1 (ACTC1) | DDD |
| 88 | ACTN2 | Actinin, alpha 2 (ACTN2) | DDD |
| 203 | AK1 | Adenylate kinase 1 (AK1) | DDD |
| 50808 | AK3 | Adenylate kinase 3 (AK3) | DDD |
| 226 | ALDOA | Aldolase A, fructose-bisphosphate (ALDOA) | DDD |
| 27063 | ANKRD1 | Ankyrin repeat domain 1 (cardiac muscle) (ANKRD1) | DDD |
| 487 | ATP2A1 | ATPase, Ca++ transporting, cardiac muscle, fast twitch 1 (ATP2A1) | DDD |
| **488** | **ATP2A2** | **ATPase, Ca++ transporting, cardiac muscle, slow twitch 2 (ATP2A2)** | **DDD** |
| 498 | ATP5A1 | ATP synthase, H+ transporting, mitochondrial F1 complex, alpha subunit 1, cardiac muscle (ATP5A1) | DDD |
| 539 | ATP5O | ATP synthase, H+ transporting, mitochondrial F1 complex, O subunit (ATP5O) | DDD |
| 93974 | ATPIF1 | ATPase inhibitory factor 1 (ATPIF1) | DDD |
| 761 | CA3 | Carbonic anhydrase III, muscle specific (CA3) | DDD |
| 844 | CASQ1 | Calsequestrin 1 (fast-twitch, skeletal muscle) (CASQ1) | DDD |
| 845 | CASQ2 | Calsequestrin 2 (cardiac muscle) (CASQ2) | DDD |
| 10983 | CCNI | Cyclin I (CCNI) | DDD |
| 948 | CD36 | CD36 molecule (thrombospondin receptor) (CD36) | DDD |
| 1073 | CFL2 | Cofilin 2 (muscle) (CFL2) | DDD |
| 400916 | CHCHD10 | Coiled-coil-helix-coiled-coil-helix domain containing 10 (CHCHD10) | DDD |
| 51142 | CHCHD2 | Coiled-coil-helix-coiled-coil-helix domain containing 2 (CHCHD2) | DDD |
| 1158 | CKM | Creatine kinase, muscle (CKM) | DDD |
| 1160 | CKMT2 | Creatine kinase, mitochondrial 2 (sarcomeric) (CKMT2) | DDD |
| 202333 | CMYA5 | Cardiomyopathy associated 5 (CMYA5) | DDD |
| 1329 | COX5B | Cytochrome c oxidase subunit Vb (COX5B) | DDD |
| 1339 | COX6A2 | Cytochrome c oxidase subunit VIa polypeptide 2 (COX6A2) | DDD |
| 1340 | COX6B1 | Cytochrome c oxidase subunit Vib polypeptide 1 (ubiquitous) (COX6B1) | DDD |
| 1345 | COX6C | Cytochrome c oxidase subunit VIc (COX6C) | DDD |
| 1349 | COX7B | Cytochrome c oxidase subunit VIIb (COX7B) | DDD |
| 1350 | COX7C | Cytochrome c oxidase subunit VIIc (COX7C) | DDD |
| 1351 | COX8A | Cytochrome c oxidase subunit 8A (ubiquitous) (COX8A) | DDD |
| 1410 | CRYAB | Crystallin, alpha B (CRYAB) | DDD |
| 8531 | CSDA | Cold shock domain protein A (CSDA) | DDD |
| 8048 | CSRP3 | Cysteine and glycine-rich protein 3 (cardiac LIM protein) (CSRP3) | DDD |
| 1537 | CYC1 | Cytochrome c-1 (CYC1) | DDD |
| 1674 | DES | Desmin (DES) | DDD |
| 11080 | DNAJB4 | DnaJ (Hsp40) homolog, subfamily B, member 4 (DNAJB4) | DDD |
| 1937 | EEF1G | Eukaryotic translation elongation factor 1 gamma (EEF1G) | DDD |
| 23741 | EID1 | EP300 interacting inhibitor of differentiation 1 (EID1) | DDD |
| 1974 | EIF4A2 | Eukaryotic translation initiation factor 4A, isoform 2 (EIF4A2) | DDD |
| 2027 | ENO3 | Enolase 3 (beta, muscle) (ENO3) | DDD |
| 346007 | EYS | Ribosomal protein S25 (RPS25) | DDD |
| 2170 | FABP3 | Fatty acid binding protein 3, muscle and heart (mammary-derived growth inhibitor) (FABP3) | DDD |
| 2167 | FABP4 | Fatty acid binding protein 4, adipocyte (FABP4) | DDD |
| **2273** | **FHL1** | **Four and a half LIM domains 1 (FHL1)** | **DDD** |
| 2274 | FHL2 | Four and a half LIM domains 2 (FHL2) | DDD |
| 2318 | FLNC | Filamin C, gamma (FLNC) | DDD |
| 5348 | FXYD1 | FXYD domain containing ion transport regulator 1 (FXYD1) | DDD |
| 2597 | GAPDH | Glyceraldehyde-3-phosphate dehydrogenase (GAPDH) | DDD |
| 2631 | GBAS | Glioblastoma amplified sequence (GBAS) | DDD |
| **2934** | **GSN** | **Gelsolin (amyloidosis, Finnish type) (GSN)** | **DDD** |
| 3032 | HADHB | Hydroxyacyl-Coenzyme A dehydrogenase/3-ketoacyl-Coenzyme A thiolase/enoyl-Coenzyme A hydratase (trifunctional protein), beta subunit (HADHB) | DDD |
| 3039 | HBA1 | Hemoglobin, alpha 1 (HBA1) | DDD |
| 3040 | HBA2 | Hemoglobin, alpha 2 (HBA2) | DDD |
| 3048 | HBG2 | Hemoglobin, gamma G (HBG2) | DDD |
| 148738 | HFE2 | Hemochromatosis type 2 (juvenile) (HFE2) | DDD |
| 192286 | HIGD2A | HIG1 hypoxia inducible domain family, member 2A (HIGD2A) | DDD |
| 3315 | HSPB1 | Heat shock 27kDa protein 1 (HSPB1) | DDD |
| 126393 | HSPB6 | Heat shock protein, alpha-crystallin-related, B6 (HSPB6) | DDD |
| 27129 | HSPB7 | Heat shock 27kDa protein family, member 7 (cardiovascular) (HSPB7) | DDD |
| **26353** | **HSPB8** | **Heat shock 22kDa protein 8 (HSPB8)** | **DDD** |
| 3514 | IGKC | Immunoglobulin kappa constant (IGKC) | DDD |
| 3535 | IGL@ | Immunoglobulin lambda locus (IGL@) | DDD |
| 3679 | ITGA7 | Integrin, alpha 7 (ITGA7) | DDD |
| 10324 | KBTBD10 | Kelch repeat and BTB (POZ) domain containing 10 (KBTBD10) | DDD |
| 11155 | LDB3 | LIM domain binding 3 (LDB3) | DDD |
| 100287143 | LOC100287143 | Hypothetical protein LOC100287143 (LOC100287143) | DDD |
| **4023** | **LPL** | **Lipoprotein lipase (LPL)** | **DDD** |
| 4060 | LUM | Lumican (LUM) | DDD |
| 4151 | MB | Myoglobin (MB) | DDD |
| 4151 | MB | Hypothetical protein LOC100289664 (LOC100289664) | DDD |
| 4256 | MGP | Matrix Gla protein (MGP) | DDD |
| 4502 | MT2A | Metallothionein 2A (MT2A) | DDD |
| 389125 | MUSTN1 | Musculoskeletal, embryonic nuclear protein 1 (MUSTN1) | DDD |
| 4604 | MYBPC1 | Myosin binding protein C, slow type (MYBPC1) | DDD |
| 4606 | MYBPC2 | Myosin binding protein C, fast type (MYBPC2) | DDD |
| 4607 | MYBPC3 | Myosin binding protein C, cardiac (MYBPC3) | DDD |
| 4619 | MYH1 | Myosin, heavy chain 1, skeletal muscle, adult (MYH1) | DDD |
| 4620 | MYH2 | Myosin, heavy chain 2, skeletal muscle, adult (MYH2) | DDD |
| 4625 | MYH7 | Myosin, heavy chain 7, cardiac muscle, beta (MYH7) | DDD |
| 4632 | MYL1 | Myosin, light chain 1, alkali; skeletal, fast (MYL1) | DDD |
| 10627 | MYL12A | Myosin, light chain 12A, regulatory, non-sarcomeric (MYL12A) | DDD |
| 4633 | MYL2 | Myosin, light chain 2, regulatory, cardiac, slow (MYL2) | DDD |
| 4634 | MYL3 | Myosin, light chain 3, alkali; ventricular, skeletal, slow (MYL3) | DDD |
| 58498 | MYL7 | Myosin, light chain 7, regulatory (MYL7) | DDD |
| **10398** | **MYL9** | **Myosin, light chain 9, regulatory (MYL9)** | **DDD** |
| 29895 | MYLPF | Myosin light chain, phosphorylatable, fast skeletal muscle (MYLPF) | DDD |
| 8736 | MYOM1 | Myomesin 1, 185kDa (MYOM1) | DDD |
| 9172 | MYOM2 | Myomesin (M-protein) 2, 165kDa (MYOM2) | DDD |
| 9499 | MYOT | Myotilin (MYOT) | DDD |
| **58529** | **MYOZ1** | **Myozenin 1 (MYOZ1)** | **DDD** |
| **51778** | **MYOZ2** | **Myozenin 2 (MYOZ2)** | **DDD** |
| 4694 | NDUFA1 | NADH dehydrogenase (ubiquinone) 1 alpha subcomplex, 1, 7.5kDa (NDUFA1) | DDD |
| 51079 | NDUFA13 | NADH dehydrogenase (ubiquinone) 1 alpha subcomplex, 13 (NDUFA13) | DDD |
| 4697 | NDUFA4 | NADH dehydrogenase (ubiquinone) 1 alpha subcomplex, 4, 9kDa (NDUFA4) | DDD |
| 4702 | NDUFA8 | NADH dehydrogenase (ubiquinone) 1 alpha subcomplex, 8, 19kDa (NDUFA8) | DDD |
| 4716 | NDUFB10 | NADH dehydrogenase (ubiquinone) 1 beta subcomplex, 10, 22kDa (NDUFB10) | DDD |
| 4725 | NDUFS5 | NADH dehydrogenase (ubiquinone) Fe-S protein 5, 15kDa (NADH-coenzyme Q reductase) (NDUFS5) | DDD |
| 4703 | NEB | Nebulin (NEB) | DDD |
| **10529** | **NEBL** | **Nebulette (NEBL)** | **DDD** |
| 91624 | NEXN | Nexilin (F actin binding protein) (NEXN) | DDD |
| 4878 | NPPA | Natriuretic peptide precursor A (NPPA) | DDD |
| 4879 | NPPB | Natriuretic peptide precursor B (NPPB) | DDD |
| 4892 | NRAP | Nebulin-related anchoring protein (NRAP) | DDD |
| **9659** | **PDE4DIP** | **Phosphodiesterase 4D interacting protein (PDE4DIP)** | **DDD** |
| 27295 | PDLIM3 | PDZ and LIM domain 3 (PDLIM3) | DDD |
| **10611** | **PDLIM5** | **PDZ and LIM domain 5 (PDLIM5)** | **DDD** |
| 5213 | PFKM | Phosphofructokinase, muscle (PFKM) | DDD |
| 5224 | PGAM2 | Phosphoglycerate mutase 2 (muscle) (PGAM2) | DDD |
| 5569 | PKIA | Protein kinase (cAMP-dependent, catalytic) inhibitor alpha (PKIA) | DDD |
| 5318 | PKP2 | Plakophilin 2 (PKP2) | DDD |
| 5350 | PLN | Phospholamban (PLN) | DDD |
| **5730** | **PTGDS** | **Prostaglandin D2 synthase 21kDa (brain) (PTGDS)** | **DDD** |
| 5837 | PYGM | Phosphorylase, glycogen, muscle (PYGM) | DDD |
| 8490 | RGS5 | Regulator of G-protein signaling 5 (RGS5) | DDD |
| **23433** | **RHOQ** | **Ras homolog gene family, member Q (RHOQ)** | **DDD** |
| 728658 | RPL13AP5 | MRNA upregulated during camptothecin-induced apoptosis of U937 cells | DDD |
| 6155 | RPL27 | Ribosomal protein L27 (RPL27) | DDD |
| 6160 | RPL31 | Ribosomal protein L31 (RPL31) | DDD |
| 6168 | RPL37A | Ribosomal protein L37a (RPL37A) | DDD |
| 6171 | RPL41 | Ribosomal protein L41 (RPL41) | DDD |
| 6171 | RPL41 | Ribosomal protein L41 (RPL41) | DDD |
| 6176 | RPLP1 | Ribosomal protein, large, P1 (RPLP1) | DDD |
| 6222 | RPS18 | Ribosomal protein S18 (RPS18) | DDD |
| 6229 | RPS24 | Ribosomal protein S24 (RPS24) | DDD |
| 57142 | RTN4 | Reticulon 4 (RTN4) | DDD |
| 6271 | S100A1 | S100 calcium binding protein A1 (S100A1) | DDD |
| 6284 | S100A13 | S100 calcium binding protein A13 (S100A13) | DDD |
| 6415 | SEPW1 | Selenoprotein W, 1 (SEPW1) | DDD |
| 10169 | SERF2 | Small EDRK-rich factor 2 (SERF2) | DDD |
| 6445 | SGCG | Sarcoglycan, gamma (35kDa dystrophin-associated glycoprotein) (SGCG) | DDD |
| 7871 | SLMAP | Sarcolemma associated protein (SLMAP) | DDD |
| 23676 | SMPX | Small muscle protein, X-linked (SMPX) | DDD |
| 10580 | SORBS1 | Sorbin and SH3 domain containing 1 (SORBS1) | DDD |
| 8470 | SORBS2 | Sorbin and SH3 domain containing 2 (SORBS2) | DDD |
| 8404 | SPARCL1 | SPARC-like 1 (hevin) (SPARCL1) | DDD |
| 10948 | STARD3 | StAR-related lipid transfer (START) domain containing 3 (STARD3) | DDD |
| 79933 | SYNPO2L | Synaptopodin 2-like (SYNPO2L) | DDD |
| 79041 | TMEM38A | Transmembrane protein 38A (TMEM38A) | DDD |
| 29765 | TMOD4 | Tropomodulin 4 (muscle) (TMOD4) | DDD |
| **7134** | **TNNC1** | **Troponin C type 1 (slow) (TNNC1)** | **DDD** |
| 7125 | TNNC2 | Troponin C type 2 (fast) (TNNC2) | DDD |
| **7135** | **TNNI1** | **Troponin I type 1 (skeletal, slow) (TNNI1)** | **DDD** |
| 7136 | TNNI2 | Troponin I type 2 (skeletal, fast) (TNNI2) | DDD |
| 7137 | TNNI3 | Troponin I type 3 (cardiac) (TNNI3) | DDD |
| 7138 | TNNT1 | Troponin T type 1 (skeletal, slow) (TNNT1) | DDD |
| **7139** | **TNNT2** | **Troponin T type 2 (cardiac) (TNNT2)** | **DDD** |
| 7140 | TNNT3 | Troponin T type 3 (skeletal, fast) (TNNT3) | DDD |
| 54543 | TOMM7 | Translocase of outer mitochondrial membrane 7 homolog (yeast) (TOMM7) | DDD |
| 7168 | TPM1 | Tropomyosin 1 (alpha) (TPM1) | DDD |
| **7169** | **TPM2** | **Tropomyosin 2 (beta) (TPM2)** | **DDD** |
| 7170 | TPM3 | Tropomyosin 3 (TPM3) | DDD |
| 10345 | TRDN | Triadin (TRDN) | DDD |
| 7273 | TTN | Titin (TTN) | DDD |
| 10975 | UQCR | Ubiquinol-cytochrome c reductase, 6.4kDa subunit (UQCR) | DDD |
| 7385 | UQCRC2 | Ubiquinol-cytochrome c reductase core protein II (UQCRC2) | DDD |
| 27089 | UQCRQ | Ubiquinol-cytochrome c reductase, complex III subunit VII, 9.5kDa (UQCRQ) | DDD |
| 129446 | XIRP2 | Xin actin-binding repeat containing 2 (XIRP2) | DDD |
| 7531 | YWHAE | Tyrosine 3-monooxygenase/tryptophan 5-monooxygenase activation protein, epsilon polypeptide (YWHAE) | DDD |
| 64397 | ZFP106 | Zinc finger protein 106 homolog (mouse) (ZFP106) | DDD |
