# Supplementary material for: Systematic Analysis of a Novel Human Renal Glomerulus-Enriched Gene Expression Dataset
Source: PLoS One. 2010 Jul 12;5(7):e11545. doi: 10.1371/journal.pone.0011545 (PMC2902524; doi:10.1371/journal.pone.0011545)
Supplement: Table S11 — Clinical and histological characteristics. Clinical and histological characteristics of patients and biopsies, respectively, with established diabetic nephropathy, focal segmental sclerosis and living donors analyzed by real-time RT-PCR (P) and oligonucleotide array based gene expression profiling (A) (for living donor). * = blood pressure before biopsy [mmHg]. (0.15 MB DOC) [file pone.0011545.s012.doc]

**Table S11**

|  |  | **Gender** | **Age [years]** | **Histology major diagnosis** | **Creatinine [mg/dl]** | **Proteinuria [g/day]** | **Hypertension** | **RR syst. (*)** | **RR diast. (*)** | **DM Type** |
| --- | --- | --- | --- | --- | --- | --- | --- | --- | --- | --- |
| **Focal Segmental Glomerulosclerosis** | | | | | |  |  |  |  |  |
|  | FSGS1 | m | 40 | FSGS | 0.90 | 4.50 | yes | 170 | 100 | no |
|  | FSGS2 | m | 26 | FSGS | 0.96 | 1.65 | no | 130 | 80 | no |
|  | FSGS3 | f | 39 | FSGS | 2.70 | 2.50 | yes | 160 | 105 | no |
|  | FSGS4 | m | 31 | FSGS | 0.80 | 5.50 | no | 125 | 70 | no |
|  | FSGS5 | f | 54 | FSGS | 0.60 | 1.90 | yes | 130 | 80 | no |
|  | FSGS6 | f | 21 | FSGS | 0.70 | 0.60 | yes | 130 | 70 | no |
|  | FSGS7 | m | 53 | FSGS | 1.20 | 7.00 | no | 120 | 80 | no |
|  | FSGS8 | f | 65 | FSGS | 1.30 | 3.00 | yes | na | na | no |
|  | FSGS9 | f | 54 | FSGS | 0.80 | 8.40 | no | na | na | no |
|  | FSGS10 | m | 22 | FSGS | 0.80 | 3.93 | yes | 120 | 80 | no |
|  | FSGS11 | m | 37 | FSGS | 2.00 | 8.80 | yes | 145 | 95 | no |
|  | FSGS12 | m | 23 | FSGS | 0.96 | 3.00 | no | 110 | 81 | no |
|  | FSGS13 | f | 66 | FSGS | 0.77 | 13.00 | no | 115 | 57 | no |
|  | FSGS14 | f | 32 | FSGS | 0.69 | 0.66 | yes | 120 | 75 | no |
|  | FSGS15 | f | 50 | FSGS | 0.79 | 2.00 | yes | 120 | 80 | no |
|  | FSGS16 | f | 63 | FSGS | 1.31 | 3.99 | yes | 160 | 85 | no |
|  | FSGS17 | m | 31 | FSGS | 1.00 | 6.00 | no | 160 | 93 | no |
|  | Mean ± SEM | 8/9 | 41.6 ± 15.5 |  | 1.1 ± 0.5 | 4.5 ± 3.3 |  | 134.3 ± 19.4 | 82.1 ± 12.3 |  |
| **Membranous nephropathy** | | | |  |  |  |  |  |  |  |
|  | MGN1 | m | 68 | MGN | 0.84 | 2.38 | yes | 160 | 90 | no |
|  | MGN2 | f | 75 | MGN | 1.20 | 9.70 | yes | 160 | 100 | no |
|  | MGN3 | f | 78 | MGN | 0.80 | 3.30 | yes | 180 | 90 | no |
|  | MGN4 | f | 25 | MGN | 0.70 | 2.50 | no | 120 | 80 | no |
|  | MGN5 | m | 44 | MGN | 0.80 | 3.76 | yes | 120 | 80 | no |
|  | MGN6 | m | 44 | MGN | 0.99 | 2.80 | yes | 120 | 80 | 2 |
|  | MGN7 | f | 39 | MGN | 0.80 | 1.60 | no | 120 | 70 | no |
|  | MGN8 | m | 50 | MGN | 2.00 | 4.20 | yes | 150 | 90 | no |
|  | MGN9 | f | 17 | MGN | 0.45 | 9.50 | no | na | na | no |
|  | MGN10 | f | 64 | MGN | 1.00 | 1.50 | yes | 150 | 90 | 2 |
|  | MGN11 | f | 58 | MGN | 1.40 | 1.30 | yes | 180 | 85 | no |
|  | MGN12 | m | 36 | MGN | 1.00 | 9.80 | na | na | na | no |
|  | MGN13 | m | 61 | MGN | 1.80 | 6.30 | na | 140 | 90 | no |
|  | MGN14 | m | 85 | MGN | 1.70 | 0.00 | yes | 110 | 60 | no |
|  | MGN15 | m | 49 | MGN | 0.90 | 0.50 | yes | 150 | 84 | no |
|  | MGN16 | m | 67 | MGN | 0.80 | 5.60 | yes | 155 | 90 | no |
|  | MGN17 | f | 48 | MGN | 0.60 | 0.50 | no | 110 | 80 | no |
|  | Mean ± SEM | 9/8 | 53.4 ± 18.6 |  | 1.1 ± 0.4 | 3.8 ± 3.3 |  | 141.7 ± 23.7 | 83.9 ± 9.7 |  |
| **Diabetic nephropathy** | | | |  |  |  |  |  |  |  |
|  | DN1 | m | 61 | DN | 1.10 | 4.30 | yes | 170 | 90 | 2 |
|  | DN2 | m | 64 | DN | 1.20 | 6.00 | yes | 149 | 90 | 2 |
|  | DN3 | m | 49 | DN | 0.80 | 1.30 | yes | 160 | 90 | na |
|  | DN4 | m | 57 | DN | 1.60 | 9.80 | yes | 190 | 90 | 1 |
|  | DN5 | f | 67 | DN | 4.80 | 2.40 | yes | na | na | 2 |
|  | DN6 | f | 59 | DN | 3.22 | 21.00 | yes | 162 | 85 | 2 |
|  | DN7 | m | 63 | DN | 2.13 | 8.60 | yes | 150 | 80 | 2 |
|  | DN8 | m | 57 | DN | 1.63 | 1.41 | yes | na | na | 2 |
|  | DN9 | m | 40 | DN | 1.57 | 1.50 | yes | 145 | 80 | 1 |
|  | DN10 | m | 60 | DN | 2.24 | 1.00 | yes | 210 | 90 | 2 |
|  | DN11 | m | 75 | DN | 1.70 | 6.24 | na | 160 | 88 | 2 |
|  | DN12 | na | na | DN | 1.40 | 10.00 | yes | na | na | 2 |
|  | DN13 | f | 63 | DN | 2.49 | 5.50 | yes | 160 | 100 | 2 |
|  | DN14 | f | 55 | DN | 7.08 | 6.67 | yes | 142 | 68 | 2 |
|  | Mean ± SEM | 9/4 | 59.2 ± 8.5 |  | 2.4 ± 1.7 | 6.1 ± 5.3 |  | 163.5 ± 20.4 | 86.5 ± 8.2 |  |
| **Nephrosclerosis** | | |  |  |  |  |  |  |  |  |
|  | NSC1 | m | 40 | NSC | 1.00 | 1.50 | no | 120 | 80 | no |
|  | NSC2 | f | 40 | NSC | 0.62 | 0.04 | no | 116 | 64 | no |
|  | NSC3 | m | 60 | NSC | 2.47 | 0.24 | yes | 120 | 80 | no |
|  | NSC4 | m | 43 | NSC | 2.18 | na | yes | 150 | 100 | no |
|  | NSC5 | f | 46 | NSC | 1.50 | 3.34 | yes | 150 | 90 | no |
|  | NSC6 | m | 51 | NSC | 2.50 | 0.50 | yes | 140 | 80 | no |
|  | NSC7 | na | na | NSC | 5.00 | 8.20 | yes | na | na | no |
|  | NSC8 | na | na | NSC | 1.10 | 0.44 | no | na | na | no |
|  | NSC9 | na | na | NSC | 1.40 | na | yes | 170 | 80 | no |
|  | NSC10 | m | 75 | NSC | 13.01 | na | yes | 160 | 80 | no |
|  | NSC11 | m | 56 | NSC | 1.09 | na | yes | 190 | 105 | no |
|  | NSC12 | m | 48 | NSC | 3.85 | na | no | 90 | 40 | no |
|  | NSC13 | f | 51 | NSC | 0.78 | na | no | na | na | no |
|  | NSC14 | m | 59 | NSC | 4.56 | na | yes | 138 | 79 | no |
|  | Mean ± SEM | 8/3 | 51.7 ± 10.4 |  | 2.9 ± 3.2 | 2.0 ± 3.0 |  | 140.4 ± 28.7 | 79.8 ± 17.3 |  |
| **Living Donor** | | |  |  |  |  |  |  |  |  |
| A+P | LD1 | f | 35 | LD | <1.1 | <0.2 | no | na | na | no |
| A | LD2 | f | 35 | LD | <1.1 | <0.2 | no | na | na | no |
| A+P | LD3 | f | 55 | LD | <1.1 | <0.2 | no | na | na | no |
| A+P | LD4 | m | 40 | LD | <1.1 | <0.2 | no | na | na | no |
| A+P | LD5 | m | 61 | LD | <1.1 | <0.2 | no | na | na | no |
| A | LD6 | f | 58 | LD | <1.1 | <0.2 | no | na | na | no |
| P | LD7 | m | 27 | LD | <1.1 | <0.2 | no | na | na | no |
| P | LD8 | m | na | LD | <1.1 | <0.2 | no | na | na | no |
| P | LD9 | f | 54 | LD | <1.1 | <0.2 | no | na | na | no |
| P | LD10 | f | 61 | LD | <1.1 | <0.2 | no | na | na | no |
| P | LD11 | f | 56 | LD | <1.1 | <0.2 | no | na | na | no |
| P | LD12 | f | 63 | LD | <1.1 | <0.2 | no | na | na | no |
| P | LD13 | f | 70 | LD | <1.1 | <0.2 | no | na | na | no |
|  | Mean ± SEM | 4/9 | 51.3 ± 13.5 |  | <1.1 | <0.2 |  |  |  |  |
